# The SaniPath Exposure Assessment Tool: A quantitative approach for assessing exposure to fecal contamination through multiple pathways in low resource urban settlements

**DOI:** 10.1371/journal.pone.0234364

**Published:** 2020-06-12

**Authors:** Suraja J. Raj, Yuke Wang, Habib Yakubu, Katharine Robb, Casey Siesel, Jamie Green, Amy Kirby, Wolfgang Mairinger, James Michiel, Clair Null, Eddy Perez, Katherine Roguski, Christine L. Moe

**Affiliations:** Center for Global Safe Water, Sanitation, and Hygiene, Rollins School of Public Health, Emory University, Atlanta, GA, United States of America; Hellenic Agricultural Organization - Demeter, GREECE

## Abstract

Inadequate sanitation can lead to exposure to fecal contamination through multiple environmental pathways and can result in adverse health outcomes. By understanding the relative importance of multiple exposure pathways, sanitation interventions can be tailored to those pathways with greatest potential public health impact. The SaniPath Exposure Assessment Tool allows users to identify and quantify human exposure to fecal contamination in low-resource urban settings through a systematic yet customizable process. The Tool includes: a project management platform; mobile data collection and a data repository; protocols for primary data collection; and automated exposure assessment analysis. The data collection protocols detail the process of conducting behavioral surveys with households, school children, and community groups to quantify contact with fecal exposure pathways and of collecting and analyzing environmental samples for *E*. *coli* as an indicator of fecal contamination. Bayesian analyses are used to estimate the percentage of the population exposed and the mean dose of fecal exposure from microbiological and behavioral data. Fecal exposure from nine pathways (drinking water, bathing water, surface water, ocean water, open drains, floodwater, raw produce, street food, and public or shared toilets) can be compared through a common metric–estimated ingestion of *E*. *coli* units (MPN or CFU) per month. The Tool generates data visualizations and recommendations for interventions designed for both scientific and lay audiences. When piloted in Accra, Ghana, the results of the Tool were comparable with that of an in-depth study conducted in the same neighborhoods and highlighted consumption of raw produce as a dominant exposure pathway. The Tool has been deployed in nine cities to date, and the results are being used by local authorities to design and prioritize programming and policy. The SaniPath Tool is a novel approach to support public-health evidence-based decision-making for urban sanitation policies and investments.

## Introduction

Sanitation services and infrastructure often fail to keep up with rapid urbanization, and as a result, the urban poor are disproportionately exposed to fecal contamination in the environment [1,2]. Open defecation, poorly constructed or maintained sanitation facilities, inadequate drainage infrastructure, insufficient water supply, and poor fecal sludge management all contribute to the urban sanitation crisis. They are exacerbated by poor solid waste management, climate change, and high population density, which in turn can increase exposure to fecal pathogens [1,3,4]. Improved and citywide inclusive urban sanitation is necessary to decrease exposure to fecal contamination and protect public health.

Improved water, sanitation, and hygiene (WASH) is expected to reduce exposure to fecal contamination, which may in turn reduce associated acute enteric illness or other adverse health effects. Several studies have associated poor WASH with a variety of health outcomes including: diarrheal disease, soil-transmitted helminth infections, vector-borne diseases, and environmental enteric dysfunction that could lead to undernutrition, stunting, and poor cognitive development [5]. However, epidemiological studies to understand the public health impact of an intervention for program planning may be limited by self-reported bias, lack of uptake information, underreporting, and under-ascertainment [6]. Exposure assessments can serve as a valuable tool by systematically identifying gaps in sanitation services that result in fecal contamination of the environment and pose a hazard to the population. Because models of the dose-response relationship between fecal pathogens and enteric infection/disease and the effect of immunity are challenging to establish, exposure to fecal contamination can be used to assess the public health impact of poor WASH [7].

Exposure to WASH-related pathogens can occur along multiple exposure pathways, whereby a pathway of exposure starts at the environmental reservoir of fecal contamination (e.g. contaminated water or food) and ends at the point of oral ingestion [8]. The majority of exposure studies focus on one or two exposure pathways to fecal contamination, but blocking a single pathway may not be sufficient to reduce total exposure to a level at which a change in health effects may be observed [9–11]. The SaniPath study, which has been ongoing since 2011, has assessed exposure to fecal contamination along multiple fecal exposure pathways in both the public and private domains [12]. During the initial phase of the SaniPath study (hereafter referred to as the “formative study”) an in-depth assessment of 17 fecal exposure pathways from either public or private spaces was conducted in four low-resource urban neighborhoods in Accra, Ghana. The rationale and data collection methods for the formative study are described in detail in Robb et. al. Briefly, extensive qualitative and quantitative behavioral data, along with microbiological data, from several different fecal exposure pathways were used to develop a novel model to assess the contributions of each pathway to total fecal exposure [13–16]. The results demonstrated that each pathway can contribute differently to total exposure to fecal contamination within a population. For example, certain pathways may contribute more to fecal exposure among adults versus children, or in one neighborhood versus another. Some pathways appear to be dominant and make important and disproportionately larger contributions to total fecal exposure relative to other pathways. Only a reduction of fecal exposure via the dominant pathway(s) can lead to a substantial reduction in the total fecal exposure, which is a necessary intermediate step to achieve improved health outcomes [10].

Information identifying dominant pathways of fecal exposure can guide intervention decisions so that programs are more effectively targeted to reduce total fecal exposure and in turn, adverse health outcomes. Despite this, the use of public health evidence to inform sanitation planning is inconsistent. Furthermore, increasing decentralization of urban sanitation initiatives and the subsequent diversity of actors in the urban sanitation sector result in highly varied sanitation program planning and policy [17]. While the formative study developed a detailed understanding of fecal contamination exposure in one city, such data collection is too resource intensive and slow to be practical for most low-resource contexts. Therefore, we used the findings and experience from the formative study to develop a simplified, reliable, and feasible approach to collect public health evidence to support sanitation decision making in low-resource settings. This manuscript outlines the methods and validation of a standardized and simplified approach for conducting fecal exposure assessments.

The SaniPath Exposure Assessment Tool (hereafter referred to as the “SaniPath Tool” or “the Tool”), was developed by adapting the protocols (behavioral surveys, environmental sampling, and laboratory processing) from the formative study to identify and compare risk of exposure to fecal contamination across multiple exposure pathways associated with inadequate sanitation and fecal sludge management. This approach follows the framework for quantitative microbial risk assessment, with an emphasis on hazard identification, exposure assessment, risk characterization, and risk management [18]. The Tool provides guidance for standardized primary data collection, automates the exposure assessment analysis, and visualizes the results in a way that is accessible and understandable to people with a variety of backgrounds. It enables users to develop a robust evidence base for advocacy and decision making in the WASH sector.

## Methods

The primary goal of the Tool is to allow users to quantify and compare exposure to fecal contamination through multiple environmental pathways for adults and children residing in an urban neighborhood. Exposure is calculated as the estimated ingestion of fecal contamination (measured by the fecal indicator, *E*. *coli*) and quantified as the amount of *E*. *coli* units ingested per month for each pathway. The primary users of the Tool are local municipal governments, water and sanitation utilities, development banks, non-profits, or other organizations working in sanitation at a local level who have access to basic laboratory facilities with necessary supplies for detecting *E*. *coli* in environmental samples, experience conducting surveys, and the ability to disseminate results to the local community and stakeholders. Users should also have identified priority communities where public health data could inform decision making and consider holding a meeting of WASH and urban development stakeholders to better align with sector priorities and leverage ongoing efforts. Typical cost of a deployment of the SaniPath Tool is $2000–4000 USD per neighborhood, depending on the local cost of personnel and supplies.

The Tool focuses exclusively on exposure to fecal contamination in the public domain, rather than the private domain, for adults and children ages 5 to 12 years. We chose this focus because: 1) the public domain is more likely to be affected by public sanitation policies and action; 2) contamination in the public domain is likely to affect private domain contamination; 3) in crowded, low-resource urban areas, the majority of behavior that leads to contact with the environment likely occurs in communal spaces [19,20]; and 4) rapid data collection is more feasible in the public domain versus the private domain. The Tool does not estimate exposures for young children (under 5) who are more likely to spend the majority of their time in the private domain with their mother or caregiver.

Within the public domain, users may collect data along nine environmental fecal exposure pathways: drinking water, bathing water, surface waters, ocean water, flood water, open drains, raw produce, street foods, and public or shared toilets (described further in Table 1). These pathways were selected based on behavioral observations from the formative study, key informant interviews in pilot field sites, feasibility of collecting environmental samples or behavioral data, and applicability of the pathways to a variety of geographical and cultural contexts.

**Table 1:** Pathway types and definitions. The table below provides standard pathway definitions for a SaniPath deployment. Users may choose to adapt definitions to better fit their local contexts (e.g. different type of drinking water rather than municipal drinking water), however, the subsequent implications for interventions should be considered.

Pathway	Definition
Drinking Water	Drinking water is the most commonly consumed municipal water source in a neighborhood. Examples include “legal” or “illegal” municipal sources from pipes within compounds, public standpoints, water kiosks, or water vendors or trucks.
Bathing Water	Bathing water is defined as the water most commonly used for bathing in the neighborhood. Examples of bathing water sources include: municipal water, surface water, or well water. Bathing water may be stored or used straight from the source.
Surface Water	Surface water includes rivers, lakes, and ponds where people may commonly go to fish, swim, wash clothes, or play.
Ocean Water	Ocean water refers to bodies of marine water where people may commonly go to fish, swim, wash clothes, or play.
Open Drain Water	Open drain water is water from an open channel carrying sewage. The open drain may also carry rainwater or floodwater.
Flood Water	Flood water is defined as stagnant water within the neighborhood that remains for at least one hour.
Public / Shared Toilet	A public toilet is accessed by any neighborhood residents. A shared toilet is accessed only by specific households. These toilets are not located within a household.
Raw Produce	Raw produce refers to vegetables that are commonly eaten without cooking. They do not have a shell or inedible peel and grow above ground. Examples include cucumber, tomato, peppers, and lettuce. Fruits are generally not eligible as many have peels that are removed prior to consumption or grow on trees above the irrigation zone.
Street Food	Street food is food prepared and sold by vendors on the street and commonly eaten within the neighborhood.

**Fig 1:**
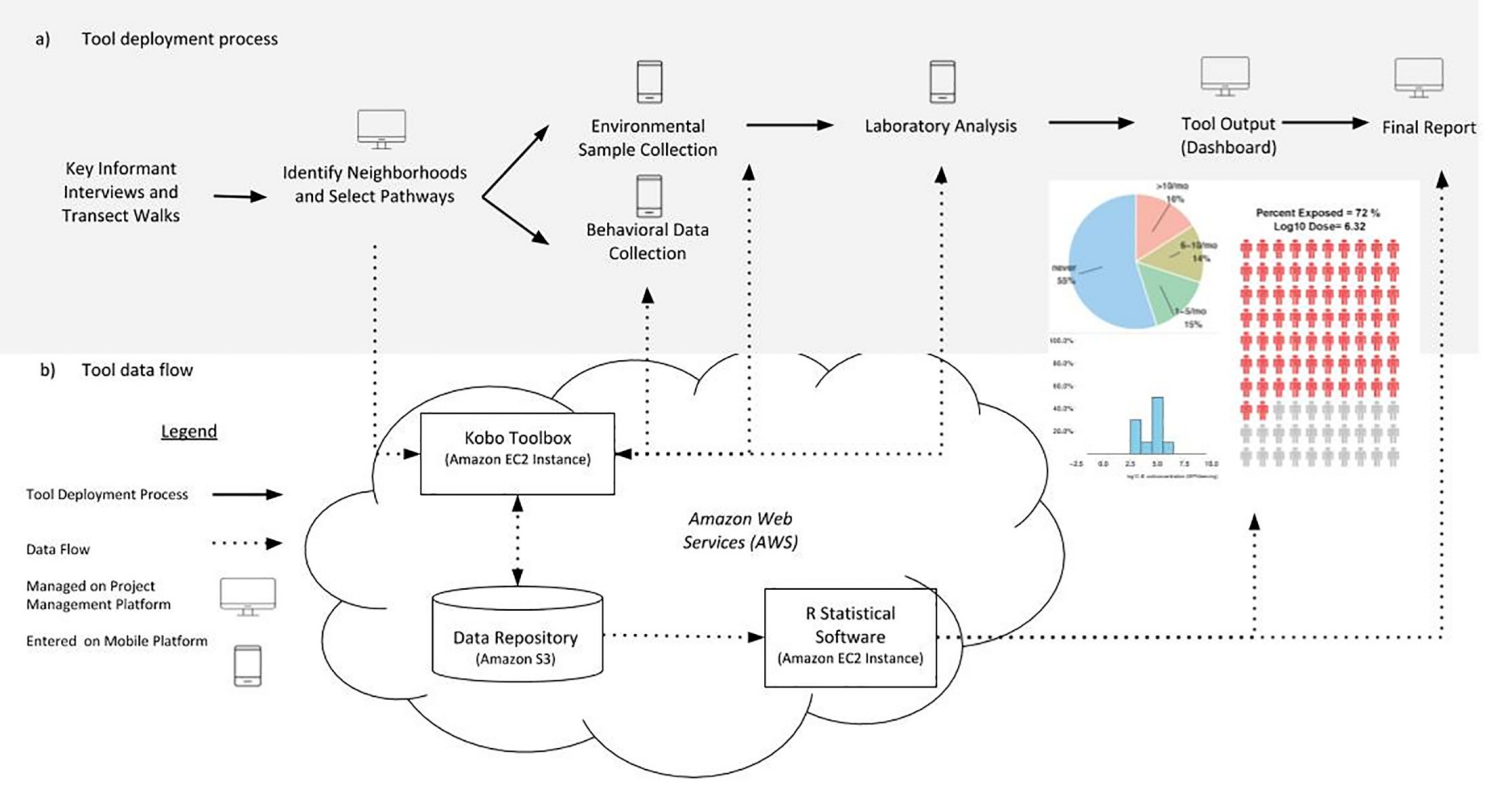
The SaniPath Tool process. a) The top half of the diagram uses solid arrows to illustrate the steps in a SaniPath Tool deployment from preliminary Key Informant Interviews to generating a final report. b) Dashed arrows represent the flow of data through different components of the web platform. EC2 Instances of Kobo Toolbox for mobile data collection and R statistical software for analysis, are hosted on cloud-based Amazon Web Services. A data repository was created using Amazon Simple Storage Service (S3) to store all the data. In some cases, data may flow in both directions (represented by a two-sided arrow). The computer and mobile icons represent whether the data are managed via the project management platform or collected via mobile phones.

The SaniPath Tool’s protocols guide users in collecting data on the frequency of exposure-related behaviors for adults and children and the contamination level of fecal indicator bacteria (*E*. *coli*) for each fecal exposure pathway. Fig 1A illustrates the data collection process, which is detailed further in subsequent sections. We consulted an advisory board of 11 international experts in public health, urban sanitation engineering, city planning, implementation, policy, and communication throughout the SaniPath Tool development process. The SaniPath Tool was created to be user-friendly, systematic, and customizable, and all protocols have been tested in a variety of urban contexts, revised, and refined over a five-year period. All protocols can be accessed at www.sanipath.org [12].

### Key informant interviews and transect walks

Key informant interviews and transect walks are conducted with city officials and community leaders at the beginning of the deployment to: 1) provide information on which pathways are relevant in the setting, 2) select appropriate environmental sampling sites, and 3) provide context for the data collection. Key informants are interviewed about local WASH infrastructure and access, fecal sludge management practices, common WASH behaviors, diet, sanitation policies, and environmental health concerns. Transect walks in target neighborhoods reveal sanitation-related risk factors and identify appropriate environmental sampling locations where local residents are most likely to interact with exposure pathways (e.g. a water pump that services a large cluster of households or a popular street food vendor). Here, a neighborhood is defined as a community with either formally- or informally-recognized boundaries. The information from these interviews and walks is used to customize the SaniPath behavioral surveys and environmental data collection forms.

### Exposure behavior surveys

Three types of survey approaches are used in the SaniPath Tool: household surveys, school surveys, and community surveys; all three types collect identical information on exposure behaviors. The three survey types can be used together or separately based on available resources and the population of interest. Household surveys are administered to the adult who manages WASH (i.e. fetches water for drinking and bathing, cleans, prepares food, etc.) and the households are selected through a systematic random sampling method. School and community surveys are administered through an anonymous voting method to groups of children and adults, respectively, who are recruited via convenience sample. In the school and community surveys, males and females are interviewed in separate groups, where culturally appropriate. We recommend conducting 100 household surveys per neighborhood, and four school and four community surveys with 15–20 participants each per neighborhood. Details on the survey protocols can be found on the SaniPath website [12].

Each survey contains questions regarding the frequency of behaviors that may lead to exposure to fecal contamination. For example, the surveys ask about the frequency of eating raw produce, drinking from water sources, having contact with open drains and flood waters, and using public or shared toilets. In household and community surveys, adults are asked about their behavior as well as that of one of their children aged 5–12. Children interviewed in the school surveys are asked about their own behavior as well as that of the adults in their household. The questions and answer choices have been tested and refined through multiple survey pilots to better quantify the frequency of behaviors in relevant time scales (per month vs. per week) and improve comprehension among respondents.

### Environmental sample collection

The environmental sampling protocols for the SaniPath Tool were designed for a variety of urban contexts. In addition to samples collected from the nine pathways included in the exposure assessment (drinking water, bathing water, surface water, ocean water, flood water, open drains, raw produce, street foods, and public or shared toilets), the sample collection protocol includes the collection of soil from public gathering spaces (e.g. playgrounds) to assess “background” levels of fecal contamination in a neighborhood. We recommend collecting and analyzing a minimum of 10 samples per environmental pathway. This sample size is small enough to be feasible, yet adequate to run the Bayesian analysis and allow the user to compare the magnitude of exposure across pathways within a neighborhood and identify the dominant pathway(s), as well as characterize the variability of individual pathways across neighborhoods.

### Laboratory processing and analysis

*E*. *coli* was chosen as the fecal indicator bacteria because of the simple laboratory methods used for identification and quantification and their widespread use as a measure of fecal contamination [21–24]. The concentration of *E*. *coli* in environmental samples can be measured using membrane filtration and m-ColiBlue24 ^®^ broth media (Hach Company, Loveland CO) or Chromocolt ^®^ Coliform Agar (EMD MilliporeSigma, Burlington, MA), or IDEXX-Colilert-24^®^ and the Quanti-Tray/2000 (IDEXX Laboratories, Westbrook, ME) [25–27]. Public or shared toilet surface swabs, raw produce, street food, and soil samples undergo a processing step prior to analysis for *E*. *coli* using protocols developed for the formative study [12]. Liquid samples do not require a processing step. Other internationally approved methods for quantifying *E*. *coli* can be substituted, but that may affect the sensitivity and reliability of the data. Two to three dilutions are analyzed for each sample in order to get a reliable estimate of concentration. To calculate the concentration of *E*. *coli* in a given sample, a selection step is used to choose dilutions for calculation (averaging) and identify any samples that present conflicting results between dilutions. The dilution protocol and process for calculating concentrations of *E*. *coli* are described in S1 Appendix.

### Statistical analyses and output

**Fig 2:**
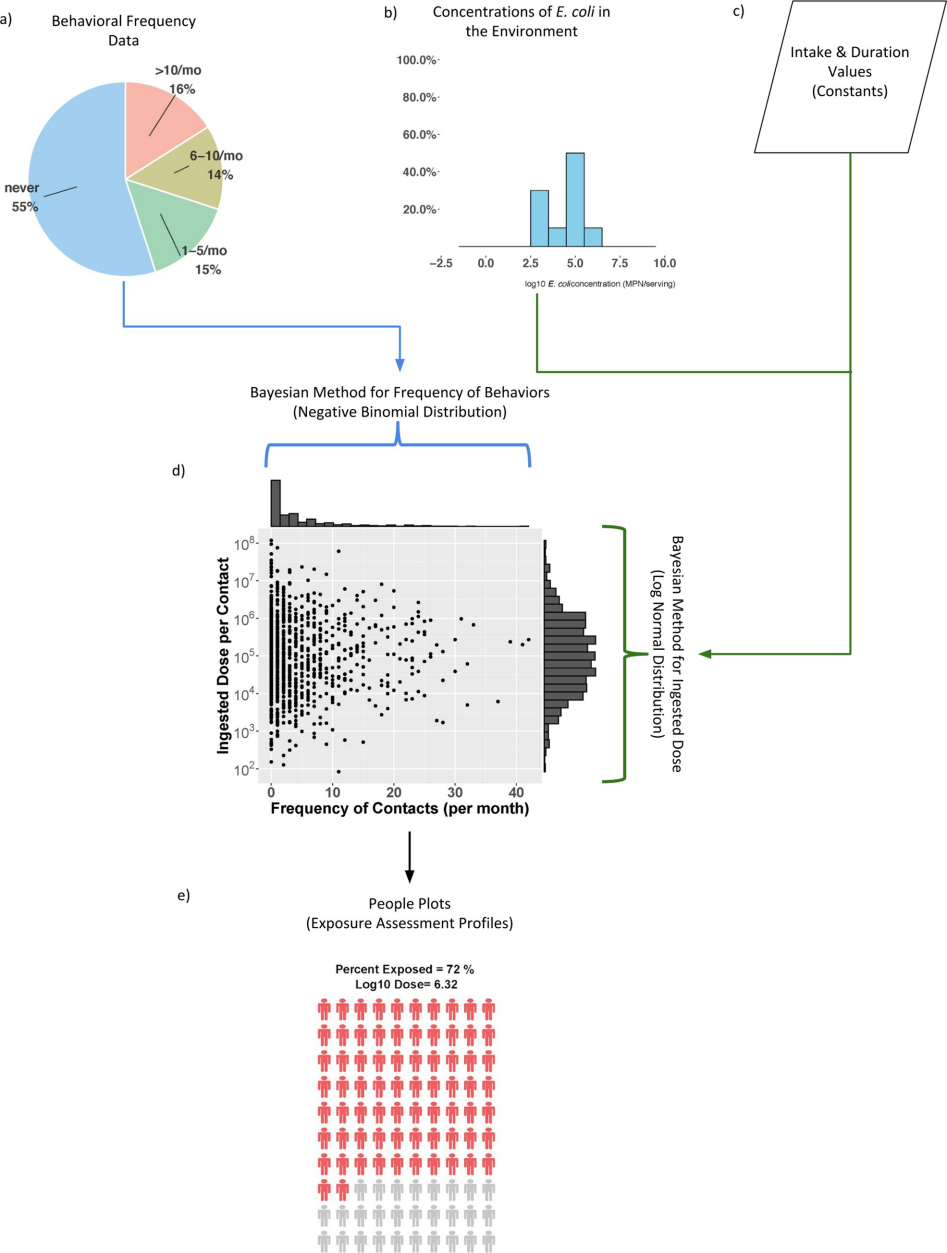
Diagram of the SaniPath Tool analysis methodology and output. a) Frequencies of behaviors associated with exposure for each pathway are represented as a pie charts in the Tool output. During analysis, behavioral survey data are used to generate a distribution of the frequency of contact for each pathway, which is assumed to be negative binomial. b) Concentrations of *E*. *coli* in each environmental pathway are represented in the Tool output as histograms. c) *E*. *coli* intake values and durations of exposure gathered from the existing literature and from the formative study, are included in the model as constants and, along with the concentrations of *E*. *coli*, are used to generate a distribution of the ingested dose per contact (assumed to be log-normal). d) 1,000 iterations of Monte Carlo simulation are run to estimate the percent of the population that is exposed, dose, and calculate exposure, as shown by the exposure assessment profiles or People Plots. e) Each red figure of a person in the People Plot represents one percent of the population (either adults or children) that is exposed to fecal contamination through a specific pathway. The relative darkness of the red color represents the magnitude of the average dose of *E*. *coli* ingested per month. Darker red represents a higher average monthly dose. The grey figures represent the percentage of the population that is not exposed to fecal contamination through this pathway.

The SaniPath Tool uses both behavioral and microbiological data to estimate exposure at a neighborhood level (Fig 2). Our exposure assessment methodology estimates distribution parameters rather than a single point estimate (e.g. the mean) of behavior frequencies and fecal contamination concentrations in the environment. Microbiological and behavioral data collected from the formative study and a pilot of the Tool in Accra, Ghana, were used to inform the assumptions included in the model. Distribution parameters are estimated by Bayesian methods using JAGS [28]. Monte Carlo simulations are used to estimate exposure to fecal contamination through each specific pathway for adults and children based on estimated distribution parameters for fecal contamination levels and frequency of behaviors, along with fixed intake volumes and duration of exposures (informed by the literature and data from the formative study). Further details on the statistical analysis and the assumptions used for the SaniPath Tool exposure assessment model can be found in S2 Appendix.

The exposure assessment uses common metrics for dose, average *E*. *coli* units (CFU or MPN) ingested per month per pathway, and the population ingesting fecal contamination, percent of the population exposed. This allows for comparison of pathways where exposure is through direct ingestion of contamination (drinking water, raw produce, street food) with pathways where exposure occurs through indirect ingestion via hand-to-mouth transfer after hand contact with contaminated surfaces (public toilets) or waters (open drains, surface waters, ocean water, flood water). Once the ingested dose and the percentage of population exposed are estimated for each pathway, the dominant pathway(s) can be identified using a systematic method described in S2 Appendix.

### Tool output

**Fig 3:**
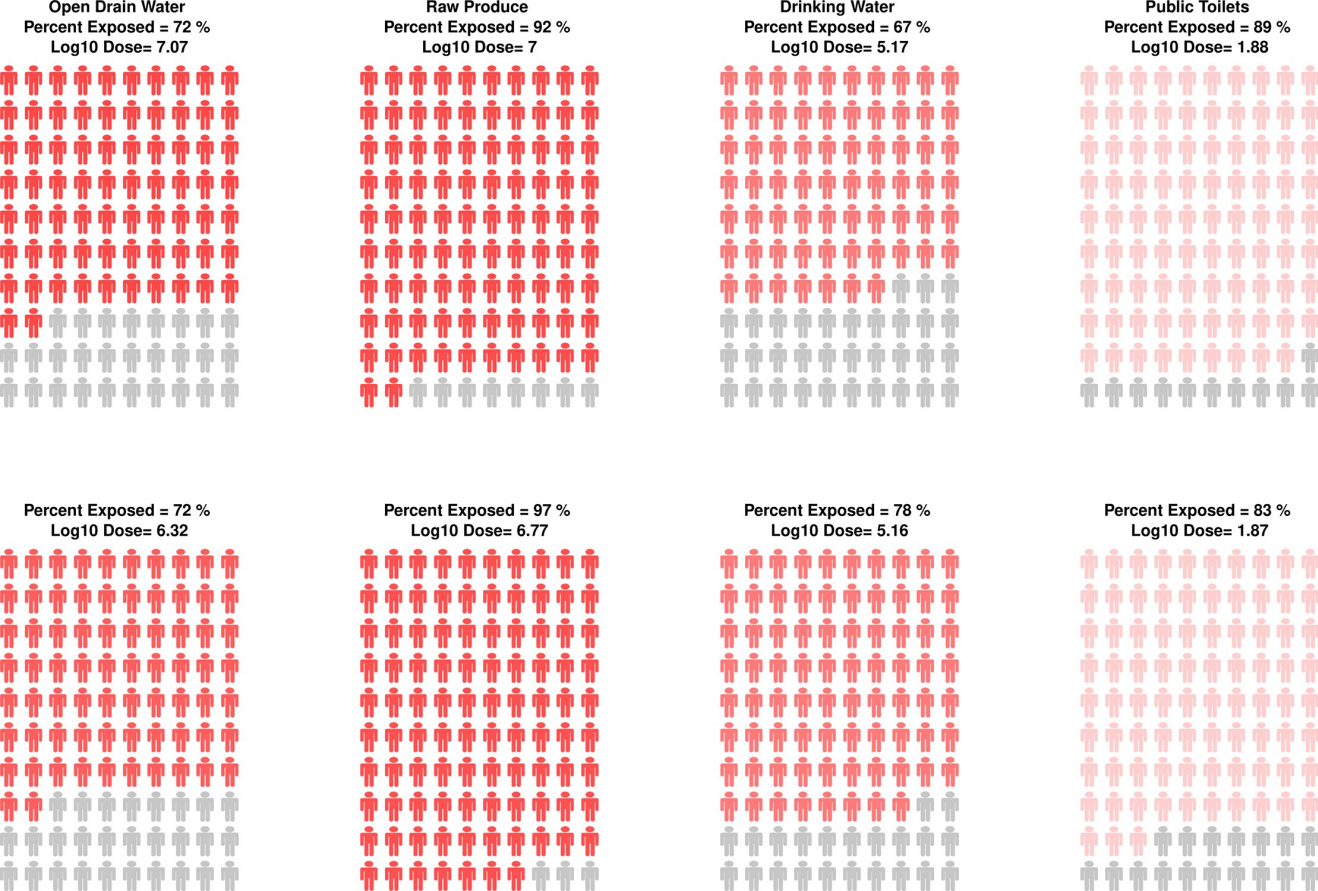
People Plot results for adults from simultaneous deployments of the SaniPath Tool by two data collection teams in Chorkor, Accra, Ghana (2016).

The SaniPath Tool assessment results are presented in three types of outputs: 1) descriptive statistics of exposure behavior frequencies for each pathway and distribution of *E*. *coli* concentrations for each type of environmental sample; 2) exposure assessment profiles; and 3) an automated report with key findings and recommendations for interventions. Descriptive statistics for behavioral frequencies and environmental fecal contamination concentrations, shown as pie charts and histograms, respectively, allow users to understand the driving forces behind exposure (Fig 2A and 2B). The exposure assessment profiles (“People Plots”), generated through the Bayesian analysis and Monte Carlo simulation, illustrate the estimated percent of the population that is exposed and the average monthly dose of *E*. *coli* ingested for those exposed via a specific pathway (Fig 2E). The People Plots are standardized infographics that allows easy visual comparison of exposure across different pathways, neighborhoods, or populations (i.e. adults or children). Fig 3 illustrates how People Plots may be used to compare exposures from multiple pathways within a single neighborhood. Lastly, an automated report describes the SaniPath Tool methods, key findings of the assessment, dominant pathway(s), interpretation of results, and recommendations for interventions based on the results. An example SaniPath Tool report can be accessed on the SaniPath website [12]. These outputs allow non-technical users, such as policy makers and local government officials, to easily view and interpret their results. More advanced users may choose to download the data and conduct additional analyses.

### The SaniPath web platform

To guide users through the deployment process (configuration, training, data collection, and analysis and interpretation), a web-based platform (https://tool.sanipath.org) comprised of sequential modules was developed [12]. The platform has three main components: 1) a project management interface; 2) a mobile data collection platform and data repository; and 3) an analytical dashboard. The platform provides a systematic framework while allowing for a degree of customization based upon information needs and local context. It is built on an integrated system of existing open source technologies that are freely available to the public, includes a customized project management interface, and a workflow for configuring and managing each deployment of the SaniPath Tool. Feedback sessions, based on the principles of human-centered design, were conducted with target users to refine the design of the web platform. The user can customize several settings (e.g. number of pathways) based upon user information needs and local context. Data are collected via downloadable mobile forms through KoBo Toolbox (Harvard Humanitarian Initiative 2018) and uploaded to the server. The analytical dashboard automatically retrieves data from the server and performs the exposure analysis on a daily basis. From the dashboard, the user can view pie charts, histograms, and People Plots and automatically generate a draft final report (Fig 1B).

### Validation studies

Behavioral surveys, environmental sampling, and laboratory analyses methods were evaluated through analysis of data from pilot deployments in Accra, Ghana, as well as analysis of formative study data. The validity and reproducibility of the Tool were assessed using several approaches: 1) comparing results from the Tool to that of the formative study; 2) examining the sampling error in estimated *E*. *coli* concentrations that is associated with the recommended sample size for environmental samples; and 3) comparing Tool results from simultaneous data collection by two different teams of enumerators in one neighborhood.

In 2013, we conducted a pilot of the SaniPath Tool in the same four neighborhoods of Accra, Ghana where the formative study was conducted [14]. The goal was to determine if the Tool could identify the same dominant exposure pathway(s) to fecal contamination as the in-depth formative study. Behavioral surveys and environmental samples were collected across five different pathways: drinking water, ocean water, open drains, raw produce, and public toilets. We validated the Tool’s methodology by comparing the exposure assessment results from the Tool to the results from the formative study. In this paper, we will focus on the validation results from the neighborhood of Shiabu because this neighborhood had not received any significant interventions since the formative study, and the data collection protocols used for the Tool deployment most closely represents the protocols in the current version of the Tool.

We also estimated the sampling error of *E*. *coli* concentrations for different sample types by simulating and resampling 10 samples from the formative study environmental data 10,000 times using R version 3.4.3 (R Core Team 2013). The purpose of this exercise was to examine the variation in the estimates of *E*. *coli* concentration from small samples for different types of environmental samples.

Lastly, in 2016, we examined the reproducibility of the SaniPath Tool results in two simultaneous deployments in Chorkor, Accra, Ghana, a neighborhood that was not previously included in the formative study. Two different groups of trained enumerators collected SaniPath Tool data in Chorkor independently and simultaneously. The dominant pathway(s), percent of population exposed to fecal contamination, and average log_10_ dose of *E*. *coli* ingested per month per pathway were compared between these two parallel deployments.

### Ethical considerations

The study protocol was approved by the Institutional Review Board at Emory University, GA, USA (Protocol number: IRB00051584) and the University of Ghana Noguchi Memorial Institute for Medical Research Institutional Review Board (Protocol number: IRB00001276). Human subject data were collected between July 2013 and October 2013, and March 2016 and July 2016 in Accra, Ghana.

## Results and discussion

The SaniPath Tool quantifies exposure to fecal contamination for people living in urban environments and identifies environmental pathways that may pose the greatest risks to human health. The Tool can generate exposure estimates to inform sanitation policies, interventions, and investments and provides rapid, useful information for advocacy. Validation studies have demonstrated that the Tool protocols can distinguish 1 log_10_ differences between exposure estimates for different environmental pathways and that results are replicable within a study site. The multi-pathway exposure assessment approach of the SaniPath Tool may reveal overlooked hazards, such as fecal contamination of raw produce or street food. In Ghana, the SaniPath Tool results have influenced planning for the inclusion of food safety in the National Urban Sanitation Strategy. In conjunction with other urban sanitation planning and decision-support tools (e.g. Sanitation Safety Plan), the SaniPath Tool can augment our understanding of urban sanitation challenges and potentially improve the impact of WASH programs by providing additional evidence on pathways and geographic areas that should be targeted for intervention.

### Validation

The SaniPath pilot in Accra, Ghana provided critical feedback for refining the survey questions and sampling protocols as well as information on the accuracy of results generated from the SaniPath Tool. Results from Shiabu demonstrated the ability of the Tool to distinguish pathways based on their exposure assessment profiles within a neighborhood and identify dominant pathways of exposure. The SaniPath Tool identified ingestion of raw produce from markets as a dominant pathway of exposure to fecal contamination for both adults and children (80% exposed, average dose 5.94 log_10_ CFU *E*.*coli*/month; 54% exposed, average dose 5.4 log_10_ CFU *E*.*coli*/month, respectively), which is consistent with findings from the formative study detailed in Wang et al. [15]. Over 50% of adults and children reported consuming raw produce at least one time per week in Shiabu. Although the SaniPath Tool pilot and the formative study in Accra used different data collection methodologies and subsequently different data were used for analysis, the similar conclusions (i.e. that consumption of raw produce was a dominant pathway) affirm the Tool’s ability to identify dominant pathways of exposure and provide useful information where an in-depth exposure assessment is not feasible.

We also explored the effect of small sample size on uncertainty in estimates of *E*. *coli* concentration in the environmental samples. While a small sample size may lead to greater uncertainty in estimates of *E*. *coli* concentration, the degree of variation in *E*. *coli* concentration differs between pathways. The results from the formative study showed that variation of *E*. *coli*, as measured by standard deviation, was large for samples of flood waters, public toilets, street food, and raw produce. The result of sampling errors shows that the standard deviation of estimates (i.e. mean) from resampling is small (<0.67) (S1 Table).

Although larger sample sizes are preferable for analysis, practical considerations and resource limitations often make large sample sizes unrealistic. Sample sizes for environmental samples are limited by funding, time needed for laboratory analyses, physical size and geography of a particular neighborhood, and the prevalence of a pathway in a neighborhood. The desire for relatively rapid results demands a balance between time allocated for sample collection and sample size. The Tool’s recommended minimum number of samples ensures that there is sufficient data to inform the exposure model and provide valid outputs. Users should collect more environmental samples where financially and logistically feasible, especially for those samples where variation in contamination may be large (i.e. public or shared toilet surfaces, drain water, flood water, raw produce, and street food).

Results from simultaneous deployments in the Chorkor neighborhood of Accra, Ghana in 2016 demonstrated that the relative ranking of the exposure pathways and characterization of dose and frequency of exposure were the same in both deployments (Fig 3). Ingestion of raw produce was the dominant pathway of fecal exposure in both deployments for adults and children. There was less than a 1 log_10_ difference in estimated dose for each of the pathways, and there was a less than about 10% difference for the estimated percent of the population exposed for each pathway between the two deployments.

The data from Chorkor, Accra demonstrates that results from the SaniPath Tool are reproducible in neighborhoods with a geographic area similar to or less than that of Chorkor (0.735km^2^). While the accuracy of the Tool may not allow us to detect small differences between different pathways or neighborhoods (less than 1 log_10_ average dose), the methods can be used to quantify and compare the relative contribution of these environmental pathways to the total exposure to fecal contamination.

### Strengths and limitations

The SaniPath Exposure Assessment Tool has many advantages for users with limited resources. At this time, we are not aware of other fecal exposure assessment tools that provide guidance for primary data collection and analysis of both behavioral surveys and environmental samples for multiple exposure pathways. Campos et.al describe a methodology to assess sanitary risks that relies on residents’ perceptions of environmental risks [29]. While examining perceptions of risk can be useful, some important hazards associated with poor sanitation or fecal sludge management may not be so obvious, such as fecal contamination of raw produce, and may be unrecognized. The SaniPath Tool’s data collection methods and analytical approach can help reveal important exposure pathways that may not otherwise come to the attention of sanitation experts and municipal authorities. Furthermore, the Tool quantifies and compares exposure along multiple pathways for both adults and children—a departure from traditional exposure assessments that generally examine exposure along a single pathway of interest (most often, drinking water). Considering multiple pathways of exposure provides a more holistic picture of risk and a more nuanced understanding of the magnitude of fecal exposure within an urban environment. Additionally, using data distributions instead of single point estimates allows the model to better quantify exposure by accounting for variability, especially with a small sample size. Lastly, the Tool can be easily adapted to different cultural contexts, employing mobile data collection and providing automated data analyses, visualization, and recommendations even for users without advanced technical knowledge or large financial resources. The unique People Plots provide population-, neighborhood- and pathway- specific information for decision making in an easily understandable infographic format for a variety of audiences. Overall, the Tool enables a relatively rapid and inexpensive assessment of fecal exposure while providing meaningful standardized information for decision-making.

The Tool has some limitations in design and analysis. First, the assessment is cross-sectional and therefore does not capture temporal and seasonal variability in fecal contamination or exposure behaviors. We recommend conducting the assessment during the peak rainy or diarrheal disease season to provide a “worst-case” scenario of exposure. Second, this assessment relies on self-reported behavior, which may be biased due to social desirability that may influence adults and children to either over- or underestimate the frequency of specific behaviors. Comprehensive enumerator training to present themselves and the survey options as neutral, as well as practicing cultural sensitivity throughout the deployment process, can help minimize these effects. There may also be differences across the survey types due to differing populations, survey methodologies, or selection criteria. Therefore, if resources are limited, care should be taken to choose the survey method that may introduce the least bias for the study population of interest (discussed further in S3 Appendix). Third, *E*. *coli* as fecal indicator bacteria do not distinguish between human and animal sources of fecal contamination, and there are some reports of possible environmental sources of *E*. *coli* [30]. While exposure to both human and animal fecal contamination pose human health risks, the intervention strategies to reduce this contamination will be different depending on the source. Future adaptations of the Tool could couple the assessment with methods to differentiate animal and human fecal contamination (e.g. microbial source tracking). Fourth, hand hygiene, food preparation, and other risk-mitigating behaviors (e.g. household water treatment) are not considered in the exposure assessment models at this time. Future refinements of the Tool could incorporate some of these modifiers of risk. Finally, published data from low- or middle- income countries on intake values and duration of activities included in the exposure models for different pathways are limited. However, as new data are available, these assumptions can be updated and improved.

### Value for policy and decision making

The results from the SaniPath Tool can be used for advocacy and to support decision making about urban sanitation policies, investments, and programs. Several countries, including India, Indonesia, and Ghana, have national policies that encourage the use of primary data to support sanitation programming [31–33]. For example, guidance under India’s 2008 National Urban Sanitation Policy (NUSP) and the Swachh Bharat Mission Guidelines include the aggregation of primary data on sanitation-related demands and perceptions, as well as their connections to environmental health, to inform future decisions [33,34]. Deployments of the Tool have also demonstrated that it is possible to collect high-quality microbiology data at scale, with limited resources [35]. The SaniPath Tool enables relatively easy environmental health data collection using standardized processes, thus lowering the barrier to evidence-based decision making and demonstrating the value of public health evidence for sanitation investment planning.

To date, the SaniPath Tool has been deployed in ten cities, and am additional city has committed resources to deploying the SaniPath Tool in the upcoming year—highlighting the increased demand for public health evidence. In Ghana, the results of the SaniPath Tool have highlighted exposure pathways, such as raw produce, that were not previously included in sanitation programming. Dissemination of SaniPath results through local media outlets created demand for more data in low-resources settings; to date, the SaniPath Tool has been deployed in 10 neighborhoods in Accra, Ghana. For local, community-based organizations, the SaniPath Tool has provided valuable evidence that is being used to advocate for the inclusion of wastewater irrigation and urban agriculture in proposals for national policy [36]. In Kumasi, Ghana, the municipal staff from the Kumasi Metropolitan Assembly deployed the Tool in four neighborhoods, and they have acted to address low-hanging issues as well as developed funding proposals for interventions targeted toward dominant pathways identified by SaniPath Tool results. In Lusaka, Zambia the results from the SaniPath Tool Assessments have reinforced the efforts of the local city council to fill shallow wells, contaminated water sources that have been implicated in recurring cholera outbreaks. Still other municipalities where the SaniPath Tool has been used, such as Kampala, Uganda and Dakar, Senegal, have ongoing dissemination efforts to facilitate translation of SaniPath results into action. The SaniPath Tool can not only support existing decision-making practices, but also has the potential to guide future urban sanitation policies.

Decisions about urban sanitation are not made in isolation, and public health evidence is one component of a wider body of evidence needed to inform smart investments. In addition to the SaniPath Tool, several other advocacy and decision-support tools have been developed in the past years for the urban sanitation sector [37–39]. The SaniPath Tool results can complement or be integrated with other tools to further support evidence-based decision making. For example, the Fecal Waste Flow Diagram, or Shit Flows Diagram (SFD), identifies points of failure in the sanitation service chain where fecal sludge enters the environment [40]. Users of the SFD may choose to use the SaniPath Tool as the next step to better understand the public health risks posed by the failures of the sanitation service chain as identified by the SFD analysis. Similarly, data from the SaniPath Tool could feed into the World Health Organization’s Sanitation Safety Plan (SSP), which includes risk identification along the sanitation chain and exposure assessment [41]. The SaniPath Tool best fits in the early stages of the urban sanitation program-planning process and complements other tools designed for selecting appropriate sanitation technologies, assessing cost-effectiveness of sanitation solutions, etc. Informed use of these tools can optimize the impact and sustainability of urban sanitation programs and technologies.

### Future directions

Since the first iteration of the SaniPath Tool, the Tool has been used in partnership with multilateral organizations, NGOs, local governments, and universities in nine different cities: Accra, Ghana (2013, 2016, 2018); Vellore, India (2014); Maputo, Mozambique (2015, 2016); Siem Reap, Cambodia (2016); Dhaka, Bangladesh (2017); Atlanta, USA (2017); Lusaka, Zambia (2018, 2019); Kumasi, Ghana (2018); Kampala, Uganda (2018); and Dakar, Senegal (2019). These deployments have explored the ability of the Tool to evaluate sanitation programs and interventions, characterize fecal exposure in varied cultural and geographical contexts, and explore differences between low- and high- income communities. Future studies will explore how to select a range of representative neighborhoods to characterize fecal exposure for an entire city and compare exposure to fecal contamination across multiple cities to understand broader trends in exposure.

Additionally, the SaniPath Tool methodology can be adapted for other, more specific, applications. For example, the Tool can be adapted to assess exposure to human-specific fecal contamination (using microbial source tracking) through multiple environmental pathways. The Tool can also be adapted to focus on specific pathogens of interest where resources and capacity are available for more complex laboratory analyses. Currently, the SaniPath Tool is being adapted to examine risks of environmental exposure to *Salmonella* Typhi and *Salmonella* Paratyphi A, and *Vibrio cholera*.

Regional SaniPath training hubs are being established at selected institutions that receive training to build capacity in the SaniPath Tool methods. The goal of these training hubs is to make deploying the SaniPath Tool, and microbiological and survey methods in general, more accessible to municipalities and other organizations that would benefit from the evidence and the capacity to collect such data. The first training hub was established in 2018 in West Africa, led by the TREND group in Accra, Ghana with laboratory support from the Water Research Institute (WRI). TREND has led or supported trainings of municipal health and environmental workers in Kumasi, Ghana, Kampala, Uganda, Lusaka, Zambia, and Dakar, Senegal. The training hubs will also play a crucial role in research translation and dissemination to ensure that public health evidence is used to inform sanitation programming and policy.

The Tool is freely and openly accessible to not only those who may want to use it, but also to those who may want to further improve or build upon it. The protocols and source code for the Tool are available online [12]. As it evolves, the Tool will benefit from new knowledge from the WASH and risk assessment communities to provide relevant and increasingly accurate estimates of exposure for decision-making and advocacy.

## Conclusion

The relationships between sanitation, environment, behavior, and health are complex. To effectively address the multiple factors that can influence population health and well-being in low-income urban settings, an understanding of fecal exposure is crucial. The SaniPath Tool provides valuable and reliable data through novel and standardized protocols grounded in rigorous scientific principles. Furthermore, the Tool results can be integrated with and complement other urban sanitation planning tools to further support evidence-based decision making about urban sanitation policies and investments. The Tool can facilitate a paradigm shift in valuing public health evidence as a critical part of sanitation planning and use exposure data to maximize health impact and increase accountability. Additionally, the Tool can decrease the barriers to evidence-based decision-making where such practices are not well established. With the recent focus on citywide inclusive sanitation, urban sanitation planning will require a greater understanding of the environmental, social, cultural, and political factors that affect sanitation quality and access. The SaniPath Tool provides support to the WASH and urban development sectors to make informed decisions and customize urban sanitation solutions to the local contexts to maximize positive impact.

## Supporting information

S1 Appendix Dilution scheme for environmental samples and calculating concentrations of *E*. *coli*. (DOCX)

S2 Appendix Details of statistical analysis and model assumptions. (DOCX)

S3 Appendix Concordance between survey types in Vellore, India. (DOCX)

S1 Table Variation in *E*. *coli* concentrations in environmental samples. The table depicts results of sampling error analysis using data from the formative study in Accra, Ghana.
(DOCX)

S1 Dataset (ZIP)
