# Supplementary material for: The SaniPath Exposure Assessment Tool: A quantitative approach for assessing exposure to fecal contamination through multiple pathways in low resource urban settlements
Source: PLoS One. 2020 Jun 12;15(6):e0234364. doi: 10.1371/journal.pone.0234364 (PMC7292388; doi:10.1371/journal.pone.0234364)
Supplement: S1 Appendix — (DOCX) [file pone.0234364.s001.docx]

# **S1 Appendix: Dilution scheme for environmental samples and calculating concentrations of *E. coli***

Due to the wide variation in fecal contamination across samples in different contexts, an adjusted dilution protocol was developed to best capture *E. coli* within the quantifiable range of the assay with limited laboratory consumables. Each environmental sample is analyzed for *E. coli* using 2-3 dilutions with 10- or 100-fold increases when a large variation in *E. coli* concentration is expected. Table 1 shows the recommended dilutions for different SaniPath samples.

**Table 1.** Recommended dilutions for quantification of *E. coli*

|  | **Dilution** | | | | |  |
| --- | --- | --- | --- | --- | --- | --- |
| **Sample Type** | **Undiluted (1:1)** | **1:10** | **1:100** | **1:1000** | **1:10,000** | **1:1,000,000** |
| **Drinking Water** | ✓ | ✓ |  |  |  |  |
| **Bathing Water** | ✓ | ✓ |  |  |  |  |
| **Surface Water** |  | ✓ | ✓ |  |  |  |
| **Ocean Water** |  | ✓ | ✓ |  |  |  |
| **Drain Water** |  |  |  |  | ✓ | ✓ |
| **Flood Water** |  |  | ✓ | ✓ | ✓ |  |
| **Raw Produce** |  | ✓ | ✓ | ✓ |  |  |
| **Street Food** |  | ✓ | ✓ | ✓ |  |  |
| **Public/Shared Toilet Swabs** |  | ✓ |  | ✓ |  |  |
| **Soil** |  |  | ✓ | ✓ |  |  |

The equivalent volume is defined as the volume of raw sample in the diluted sample that enters the assay. For example, a 1:100 dilution refers to 1 unit of raw sample diluted 100 times (with 99 units of PBS). When 100mL of the diluted sample is tested by membrane filtration or IDEXX, the equivalent volume is 1mL. The equivalent volume for dilution $k$ is denoted as $V_{k}$. When the membrane filtration method is used one of three outcomes can occur: (1) the count, as the number of colony forming units (CFUs), can be between 0 -200 CFUs; (2) too numerous to count (TNTC), that is to say a count greater than 200 CFUs; and (3) too dirty to count (TDTC). The count for dilution $k$ is denoted as $N_{k}$. When the IDEXX method is used, the resulting count can be between < 1 to > 2419.6 Most Probable Numbers (MPN), estimated from the numbers of big wells and small wells that fluoresce under UV light. The MPN for dilution $k$ is denoted as $M_{k}$.

Results from all dilutions analyzed for a single sample are selected and used to calculate the *E. coli* concentration of the sample. During the selection step, the results deemed unlikely and/or conflicting are excluded. This selection step for identifying questionable data differs for membrane filtration and IDEXX due to variations in the expected range of *E. coli* counts.

**Membrane Filtration**

Measurements of *E. coli* across dilutions for a single sample analyzed using membrane filtration are prioritized using the following ranking: 1) 10-200, 2) 1-10, 3) 0 or TNTC. For example, if at least one membrane filtration plate has a reading of 10-200 CFUs, only those plates with 10-200 CFUs are selected for the next calculation step. For censored data, which includes plates with readings of 0 or TNTC, 0 is replaced with 0.5 CFU and TNTC is replaced with 200 CFU for the following calculation step. The dilutions selected are denoted as $S$ and the number of dilutions selected is denoted as $n_{s}$.

The concentration of E. coli for sample $a$:

$$C_{a} = \frac{\sum_{k\in S}^{S} \frac{N_{k}}{V_{k}}}{n_{s}}\cdot d_{sample} (1)$$

Where the $d_{sample}$ is the factor for back calculation to the concentration of the raw sample based on its sample type (Table 2).

**IDEXX**

Measurements of *E. coli* across dilutions for a single sample analyzed using the IDEXX method are prioritized using the following ranking: 1) 1-200 MPN, 2) 200-2419.6 MPN, 3) “<1” or “>2419.6” MPN. For example, if you have a tray or trays with a reading of 1-200 MPN, only those plates with 1-200 MPN are selected for the next calculation step, and so on. Censored data with a reading of <1 MPN is replaced with 0.5 MPN and readings of >2419.6 MPN are replaced with 2419.6 MPN for calculation step. The selected dilutions are denoted as $S$ and the number of dilutions selected is denoted as $n_{s}$.

The concentration of E. coli for sample $a$:

$$C_{a} = \frac{\sum_{k\in S}^{S} \frac{M_{k}}{V_{k}}}{n_{s}}\cdot d_{sample} (2)$$

Where the $d_{sample}$ is the factor for back calculation to the concentration of the raw sample based on its sample type.

**Table 2.** Back calculation factors of *E. coli* concentration by environmental sample type

| Sample Type | Back Calculation Factor |
| --- | --- |
| Drinking Water | 100 |
| Bathing Water | 100 |
| Surface Water | 100 |
| Ocean Water | 100 |
| Drain Water | 100 |
| Flood Water | 100 |
| Raw Produce | 500 |
| Street Food | Street Food weight/10g |
| Public or Shared Toilet Swabs | 14 |
| Soil | 2 |
