# Supplementary material for: The SaniPath Exposure Assessment Tool: A quantitative approach for assessing exposure to fecal contamination through multiple pathways in low resource urban settlements
Source: PLoS One. 2020 Jun 12;15(6):e0234364. doi: 10.1371/journal.pone.0234364 (PMC7292388; doi:10.1371/journal.pone.0234364)
Supplement: S2 Appendix — (DOCX) [file pone.0234364.s002.docx]

# **S2 Appendix: Details of statistical analysis and model assumptions**

**Exposure Assessment**

The concentration of *E. coli* in a specific sample type is assumed to follow a lognormal distribution, while the frequency of behavior that may lead to exposure is assumed to follow a negative binomial distribution. The parameters associated with the distribution of concentrations vary by sample type and neighborhood, and the parameters associated with the distribution of behavior frequencies vary by age group (adults/children), pathway, and neighborhood.

1,000 iterations of Monte Carlo simulation are conducted based on Bayesian estimates of distribution parameters related to concentrations of *E. coli* in the environmental samples and frequencies of behavior along with intake volumes and durations of exposure (informed by the literature and data from the formative study). They generate estimates of exposure to fecal contamination through a specific exposure pathway for adults or children. The intake volumes and durations of exposure (Table 1) are assumed to be fixed in the model due to limited information in the literature about their variation.

**Table 1.** Assumptions and factors used in the SaniPath Tool exposure model

| **Pathway** | **Age Group** | **Intake Volume (mL) or Percent Consumed** | **Intake Time**  **(minutes unless specified)** | **Duration of Event (minutes)** | **mL/Event or percent consumed** | **Multiplication Factor** | **Unit for Average CFU/MPN of E. coli** |
| --- | --- | --- | --- | --- | --- | --- | --- |
| **Drinking Water** | Adults | 1043^1^ | Day | - | 1043 | 10.43 | Per 100mL |
|  | Children | 414^1^ | Day | - | 414 | 4.14 |  |
| **Bathing Water** | Adults | 17.6^2^ | 60 | 17^3^ | 4.987 | 0.0499 | Per 100mL |
|  | Children | 28.5^2^ | 60 | 21^3^ | 9.975 | 0.09975 |  |
| **Surface Water** | Adults | 3.7^4^ | 60 | 60^2^ | 3.700 | 0.0370 | Per 100ml |
|  | Children | 49^5^ | 60 | 25^2^ | 20.417 | 0.2042 |  |
| **Ocean Water** | Adults | 3.7^4^ | 60 | 25^2^ | 1.542 | 0.0154 | Per 100mL |
|  | Children | 49^5^ | 60 | 25^2^ | 20.417 | 0.2042 |  |
| **Drain Water** | Adults | 0.06^2^ | Event | - | 0.06 | 0.0006 | Per 100mL |
|  | Children | 1^2^ | Event | - | 1.0 | 0.01 |  |
| **Flood Water** | Adults | 0.06^2^ | Event | - | 0.06 | 0.0006 | Per 100mL |
|  | Children | 1^2^ | Event | - | 1.0 | 0.01 |  |
| **Raw Produce** | Adults | 100%^6^ | Event | - | 100% | 1.0 | Per serving |
|  | Children | 50%^6^ | Event | - | 50% | 0.5 |  |
| **Street Food** | Adults | 100%^6^ | Event | - | 100% | 1.0 | Per serving |
|  | Children | 50%^6^ | Event | - | 50% | 0.5 |  |
| **Public/ Shared Toilets** | Adults | 3.40%^2^ | Event | - | 3.40% | 0.034 | Per swab |
|  | Children | 3.40%^2^ | Event | - | 3.40% | 0.034 |  |

Table 1 provides the values that are used to calculate the intake volume per event for each exposure pathway. Separate values are used for adults and children based on different estimates of model parameters. Model parameters for intake volume, intake time, and duration of event are combined for adults and children for the bathing water, surface water, and ocean water pathways to calculate the expected volume of water ingested per event using the following formula:

$$\frac{mL}{event}= \frac{Intake Volume}{Intake Time} \times Duration of Event ( 1)$$

Volume of water ingested per event for drinking water is calculated using an intake time-scale of day, while that of drain water and flood water is calculated using intake time-scales of event, therefore, the volume ingested per event is equal to the intake volume estimate. Produce, street food, and public/shared toilet pathways are measured on the unit of percent consumed per event and are directly equivalent to the estimate of percent consumed. The intake volume or percent consumed per event is divided by 100 to attain the multiplication factor for each exposure pathway (excluding produce, street food, and public or shared toilets). The dose of exposure, estimated as CFU or MPN ingested per event, is calculated by multiplying the average *E. coli* concentration from environmental samples in the appropriate units of CFU or MPN per 100mL, per serving, or per swab, by the multiplication factor for the relevant exposure pathway.

**Determining the Dominant Pathway(s)**

To determine the dominant pathway(s) of exposure, the dose and percent of the population exposed are multiplied to calculate "exposure", which is log-transformed and denoted as $E$. All pathways with an $E$ larger than 10 (high risk) or that fall within a log 1 range around the maximum $E$ value will be considered dominant pathways (e.g. $E_{max}$= 5, therefore dominant pathways have $E\in[4, 5]$). However, if $E$ is below 1 (low risk) for all pathways, then there is no single dominant pathway. Standards for exposure are not available in the literature for many environmental pathways, therefore, the cutoffs for dominance are set at 1 (low risk) and 10 (high risk) based on our formative and pilot studies of the Tool in Accra. The cutoffs may be revised in future versions of the Tool as the literature evolves and more information is gathered.

1. U.S. EPA. Exposure Factors Handbook 2011 Edition (Final Report). 2011.

2. SaniPath. SaniPath Phase 1 Data.

3. U.S. EPA. *Descriptive Statistics from a Detailed Analysis of the National Human Activity Pattern Survey (NHAPS) Responses*. Washington, DC; 1996. doi:EPA/600/R-96/148

4. Dorevitch S, Panthi S, Huang Y, et al. Water ingestion during water recreation. *Water Res*. 2011;45(5):2020-2028. doi:10.1016/j.watres.2010.12.006

5. Dufour AP, Evans O, Behymer TD, Cantú R. Water ingestion during swimming activities in a pool: a pilot study. *J Water Health*. 2006;4(4):425-430. http://www.ncbi.nlm.nih.gov/pubmed/17176813. Accessed October 16, 2019.

6. Percent consumed estimate is based on serving size of produce or street food that an adult could be expected to eat. For child estimates, the serving size is estimated to be half that of the adult portion.
