# Supplementary material for: The SaniPath Exposure Assessment Tool: A quantitative approach for assessing exposure to fecal contamination through multiple pathways in low resource urban settlements
Source: PLoS One. 2020 Jun 12;15(6):e0234364. doi: 10.1371/journal.pone.0234364 (PMC7292388; doi:10.1371/journal.pone.0234364)
Supplement: S3 Appendix — (DOCX) [file pone.0234364.s003.docx]

# **S4 Appendix: Concordance between survey types in Vellore, India**

We conducted a second pilot in two neighborhoods in Vellore, India (Old Town and Chinna Allapuram). Details of the neighborhoods are described in John et. Al.^1^ Data from Vellore were analyzed to test the independence between responses of frequency of behavior and survey type. Chi-Square tests and Fisher's exact tests, wherever appropriate, were used and analysis was performed in SAS 9.3 (SAS Institute, Cary NC).

Survey responses from household, school, and community surveys were compared and results showed challenges with consistency of results between the survey types. In both neighborhoods, responses of frequency of behavior and survey type were not independent. The reported frequencies of contact with contamination were significantly different (p < 0.05) when the three different survey types were compared to each other (Table 1). Of the ten questions asked in both neighborhoods about behavioral frequencies, responses to eight questions about adult behaviors were significantly different between both household and school surveys and community and household surveys. Furthermore, responses to six questions were significantly different between community and school surveys. Similar significant differences were found for questions about child behavior across surveys.

Several factors could have contributed to inconsistencies between the three survey methodologies outlined in the Tool. Firstly, the three survey types were administered to different populations (adults in charge of household WASH, adult men and women, orchildren aged 10-12). The variation in types of respondents could have resulted in an information bias. For example, a child may not be aware of all their parents’ behaviors as they spend most of their days at school and vice versa. Furthermore, there were different selection biases for these three types of surveys. In this particularly study site, 25 of the 100 households for the household survey were pre-selected based on the results of a hygiene survey conducted prior to the SaniPath Tool deployment. The remaining 75 households were selected as per the SaniPath Tool’s recommendations for systematic random sampling, whereby every eighth household in a neighborhood was selected for household surveys and of that only those that meet the inclusion criteria of having an eligible child aged 5-12 years were surveyed. In contrast, a convenience sample was used for school or community surveys. Lastly, there was a difference in the different survey methods. While the household surveys consisted of one-on-one interviews with a household member, both the school and community surveys were administered in group settings and employed an anonymous voting method to elicit responses. The instructions and questions were standardized for all three survey types, but there may have been some variation due to how the survey was administered by the field enumerator and the influence of having others present while voting, despite efforts to anonymize the survey and limit discussion about responses.

Despite the significant difference in responses between the three survey types, the need for simple survey methods that can provide relatively accurate data surpasses the limitations of the three methods. Ultimately for the Vellore pilot, we combined the results of all three survey types for all analysis for a given pathway (now the default setting in the Tool). We recommend users of the SaniPath Tool administer all three survey types to reduce bias from any single survey and reap the benefits of more data. However, if resources are limited, we recommend choosing which surveys to administer based on the primary population of interest. Ongoing analysis across multiple cities that have deployed the SaniPath Tool may help us further understand the differences across the three survey types.

^1^ John SM, Thomas RJ, Kaki S, et al. Establishment of the MAL-ED Birth Cohort Study Site in Vellore , Southern India. 2018;59(August):295-299. doi:10.1093/cid/ciu390

**Table 1.** Comparisons of survey responses by pathway, age, and survey type in Accra, Ghana (** indicates Chi-Square test, others are Fisher’s Exact*)

| **Neighborhood** | **Pathway** | **Age** | **Answer Options** | **Household** | **School** | **P- Value** | **Community** | **Household** | **Test Value** | **Community** | **School** | **Test Value** |
| --- | --- | --- | --- | --- | --- | --- | --- | --- | --- | --- | --- | --- |
| Old Town | Open Drain Water | Adults | More than 10 times every month | 13 (13%) | 1 (2.44%) | 0.0044 | 26 (41.94%) | 13 (13%) | <.0001 | 26 (41.94%) | 1 (2.44%) | <.0001 |
|  |  |  | 6-10 times every month | 13 (13%) | 5 (12.2%) |  | 4 (6.45%) | 13 (13%) |  | 4 (6.45%) | 5 (12.2%) |  |
|  |  |  | 1-5 times every month | 52 (52%) | 14 (34.15%) |  | 7 (11.29%) | 52 (52%) |  | 7 (11.29%) | 14 (34.15%) |  |
|  |  |  | Never | 22 (22%) | 21 (51.22%) |  | 25 (40.32%) | 22 (22%) |  | 25 (40.32%) | 21 (51.22%) |  |
| Old Town | Open Drain Water | Children | More than 10 times every month | 15 (15.46%) | 10 (11.63%) | <.0001* | 9 (47.37%) | 15 (15.46) | 0.0014 | 9 (47.37%) | 10 (11.63%) | 0.0022 |
|  |  |  | 6-10 times every month | 17 (17.53%) | 30 (34.88%) |  | 3 (15.79%) | 17 (17.53%) |  | 3 (15.79%) | 30 (34.88%) |  |
|  |  |  | 1-5 times every month | 54 (55.67%) | 30 (34.88%) |  | 3 (15.79%) | 54 (55.67%) |  | 3 (15.79%) | 30 (34.88%) |  |
|  |  |  | Never | 11 (11.34%) | 38 (44.19%) |  | 4 (21.05%) | 11 (11.34%) |  | 4 (21.05%) | 38 (44.19%) |  |
| Chinna Allapuram | Open Drain Water | Adult | More than 10 times every month | 3 (3%) | 3 (5.26%) | 0.0005 | 10 (20%) | 3 (3%) | 0.001 | 10 (20%) | 3 (5.26%) | 0.017 |
|  |  |  | 6-10 times every month | 4 (4%) | 7 (12.88%) |  | 1 (2%) | 4 (4%) |  | 1 (2%) | 7 (12.88%) |  |
|  |  |  | 1-5 times every month | 48 (48%) | 10 (17.54%) |  | 13 (26%) | 48 (48%) |  | 13 (26%) | 10 (17.54%) |  |
|  |  |  | Never | 45 (45%) | 37 (64.91%) |  | 26 (52%) | 45 (45%) |  | 26 (52%) | 37 (64.91%) |  |
| Chinna Allapuram | Open Drain Water | Children | More than 10 times every month | 7 (7.61%) | 6 (8.45%) | 0.0312* | 4 (36.36%) | 7 (7.61%) | 0.0646 | 4 (36.36%) | 6 (8.45%) | 0.0464 |
|  |  |  | 6-10 times every month | 7 (7.61%) | 10 (14.08%) |  | 0 (0%) | 7 (7.61%) |  | 0 (0%) | 10 (14.08%) |  |
|  |  |  | 1-5 times every month | 48 (52.17%) | 21 (29.58%) |  | 3 (27.27%) | 48 (52.17%) |  | 3 (27.27%) | 21 (29.58%) |  |
|  |  |  | Never | 30 (32.61%) | 34 (47.89%) |  | 11 (10.68%) | 30 (32.61%) |  | 11 (10.68%) | 34 (47.89%) |  |
| Old Town | Flood Water | Adults | More than 10 times every month | 26 (26%) | 9 (16.07%) | 0.0169* | 29 (46.77%) | 26 (26%) | 0.0002* | 29 (46.77%) | 9 (16.07%) | 0.0019* |
|  |  |  | 6-10 times every month | 14 (14%) | 5 (8.93%) |  | 5 (8.06%) | 14 (14%) |  | 5 (8.06%) | 5 (8.93%) |  |
|  |  |  | 1-5 times every month | 42 (42%) | 19 (33.93%) |  | 8 (12.9%) | 42 (42%) |  | 8 (12.9%) | 19 (33.93%) |  |
|  |  |  | Never | 18 (18%) | 23 (41.07%) |  | 20 (32.26%) | 18 (18%) |  | 20 (32.26%) | 23 (41.07%) |  |
| Old Town | Flood Water | Children | More than 10 times every month | 15 (15.96%) | 10 (12.5%) | 0.4537* | 8 (66.67%) | 15 (15.96%) | 0.001 | 8 (66.67%) | 10 (12.5%) | 0.0004 |
|  |  |  | 6-10 times every month | 13 (13.83%) | 8 (10%) |  | 1 (8.33%) | 13 (13.83%) |  | 1 (8.33%) | 8 (10%) |  |
|  |  |  | 1-5 times every month | 48 (51.06%) | 39 (48.75%) |  | 1 (8.33%) | 48 (51.06%) |  | 1 (8.33%) | 39 (48.75%) |  |
|  |  |  | Never | 18 (19.15%) | 23 (28.75%) |  | 2 (16.67%) | 18 (19.15%) |  | 2 (16.67%) | 23 (28.75%) |  |
| Chinna Allapuram | Flood Water | Adults | More than 10 times every month | 12 (12%) | 8 (16.33%) | 0.4325 | 12 (24%) | 12 (12%) | 0.2840* | 12 (24%) | 8 (16.33%) | 0.3964 |
|  |  |  | 6-10 times every month | 18 (18%) | 4 (8.16%) |  | 8 (16%) | 18 (18%) |  | 8 (16%) | 4 (8.16%) |  |
|  |  |  | 1-5 times every month | 29 (29%) | 15 (30.61%) |  | 14 (28%) | 29 (29%) |  | 14 (28%) | 15 (30.61%) |  |
|  |  |  | Never | 41 (41%) | 22 (44.9%) |  | 16 (32%) | 41 (41%) |  | 16 (32%) | 22 (44.9%) |  |
| Chinna Allapuram | Flood Water | Children | More than 10 times every month | 14 (14.74%) | 18 (25.35%) | 0.0605* | 3 (25%) | 14 (14.74%) | 0.2538 | 3 (25%) | 18 (25.35%) | 0.9442 |
|  |  |  | 6-10 times every month | 11 (11.58%) | 9 (12.68%) |  | 2 (16.67%) | 11 (11.58%) |  | 2 (16.67%) | 9 (12.68%) |  |
|  |  |  | 1-5 times every month | 29 (30.53%) | 27 (38.03%) |  | 5 (41.67%) | 29 (30.53%) |  | 5 (41.67%) | 27 (38.03%) |  |
|  |  |  | Never | 41 (43.16%) | 17 (23.94%) |  | 2 (16.67%) | 41 (43.16%) |  | 2 (16.67%) | 17 (23.94%) |  |
| Old Town | Drinking Water | Adults | Drink every day | 69 (69.7%) | 57 (81.43%) | 0.2008 | 35 (79.55%) | 69 (69.7%) | 0.2793 | 35 (79.55%) | 57 (81.43%) | 0.0947 |
|  |  |  | Drink 4-6 days a week | 7 (7.07%) | 5 (7.14%) |  | 0 (0%) | 7 (7.07%) |  | 0 (0%) | 5 (7.14%) |  |
|  |  |  | Drink 1-3 days a week | 10 (10.1%) | 2 (2.86%) |  | 5 (11.36%) | 10 (10.1%) |  | 5 (11.36%) | 2 (2.86%) |  |
|  |  |  | Never drink | 13 (13.13%) | 6 (8.57%) |  | 4 (9.09%) | 13 (13.13%) |  | 4 (9.09%) | 6 (8.57%) |  |
| Old Town | Drinking Water | Children | Drink every day | 69 (69%) | 62 (79.49%) | 0.3939 | 19 (79.17%) | 69 (69%) | 0.0042 | 19 (79.17%) | 62 (79.49%) | 0.0346 |
|  |  |  | Drink 4-6 days a week | 5 (5%) | 4 (5.13%) |  | 5 (20.83%) | 5 (5%) |  | 5 (20.83%) | 4 (5.13%) |  |
|  |  |  | Drink 1-3 days a week | 10 (10%) | 4 (5.13%) |  | 0 (0%) | 10 (10%) |  | 0 (0%) | 4 (5.13%) |  |
|  |  |  | Never drink | 16 (16%) | 8 (10.26%) |  | 0 (0%) | 16 (16%) |  | 0 (0%) | 8 (10.26%) |  |
| Chinna Allapuram | Drinking Water | Adults | Drink every day | 82 (84.54%) | 37 (64.91%) | 0.0005 | 30 (69.77%) | 82 (84.54%) | 0.0004 | 30 (69.77%) | 37 (64.91%) | 0.5683 |
|  |  |  | Drink 4-6 days a week | 0 (0%) | 6 (10.53%) |  | 3 (6.98%) | 0 (0%) |  | 3 (6.98%) | 6 (10.53%) |  |
|  |  |  | Drink 1-3 days a week | 2 (2.06%) | 6 (10.53%) |  | 7 (16.28%) | 2 (2.06%) |  | 7 (16.28%) | 6 (10.53%) |  |
|  |  |  | Never drink | 13 (13.4%) | 8 (14.04%) |  | 3 (6.98%) | 13 (13.4%) |  | 3 (6.98%) | 8 (14.04%) |  |
| Chinna Allapuram | Drinking Water | Children | Drink every day | 83 (83.84%) | 39 (59.09%) | <.0001 | 9 (52.94%) | 83 (83.84%) | 0.0006 | 9 (52.94%) | 39 (59.09%) | 0.2462 |
|  |  |  | Drink 4-6 days a week | 0 (0%) | 13 (19.7%) |  | 1 (5.88%) | 0 (0%) |  | 1 (5.88%) | 13 (19.7%) |  |
|  |  |  | Drink 1-3 days a week | 0 (0%) | 5 (7.58%) |  | 2 (11.76%) | 0 (0%) |  | 2 (11.76%) | 5 (7.58%) |  |
|  |  |  | Never drink | 16 (16.16%) | 9 (13.64%) |  | 5 (29.41%) | 16 (16.16%) |  | 5 (29.41%) | 9 (13.64%) |  |
| Old Town | Public Toilets | Adults | More than 10 times every month | 18 (18%) | 3 (5.36%) | 0.023 | 23 (37.1%) | 18 (18%) | 0.0012* | 23 (37.1%) | 3 (5.36%) | <.0001 |
|  |  |  | 6-10 times every month | 5 (5%) | 7 (12.5%) |  | 6 (9.68%) | 5 (5%) |  | 6 (9.68%) | 7 (12.5%) |  |
|  |  |  | 1-5 times every month | 31 (31%) | 25 (44.64%) |  | 5 (8.06%) | 31 (31%) |  | 5 (8.06%) | 25 (44.64%) |  |
|  |  |  | Never | 46 (46%) | 21 (37.5%) |  | 28 (45.16%) | 46 (46%) |  | 28 (45.16%) | 21 (37.5%) |  |
| Old Town | Public Toilets | Children | More than 10 times every month | 13 (13.68%) | 13 (16.25%) | 0.3788 | 11 (61.11%) | 13 (13.68%) | 0.0002 | 11 (61.11%) | 13 (16.25%) | 0.0017 |
|  |  |  | 6-10 times every month | 15 (15.79%) | 11 (13.75%) |  | 0 (0%) | 15 (15.79%) |  | 0 (0%) | 11 (13.75%) |  |
|  |  |  | 1-5 times every month | 18 (18.95%) | 23 (28.75%) |  | 3 (16.67%) | 18 (18.95%) |  | 3 (16.67%) | 23 (28.75%) |  |
|  |  |  | Never | 49 (51.58%) | 33 (41.25%) |  | 4 (22.22%) | 49 (51.58%) |  | 4 (22.22%) | 33 (41.25%) |  |
| Chinna Allapuram | Public Toilets | Adults | More than 10 times every month | 4 (4%) | 2 (3.23%) | <.0001 | 15 (28.3%) | 4 (4%) | <.0001 | 15 (28.3%) | 2 (3.23%) | <.0001 |
|  |  |  | 6-10 times every month | 4 (4%) | 21 (33.87%) |  | 1 (1.89%) | 4 (4%) |  | 1 (1.89%) | 21 (33.87%) |  |
|  |  |  | 1-5 times every month | 51 (51%) | 7 (11.29%) |  | 12 (22.64%) | 51 (51%) |  | 12 (22.64%) | 7 (11.29%) |  |
|  |  |  | Never | 41 (41%) | 32 (51.61%) |  | 25 (47.17%) | 41 (41%) |  | 25 (47.17%) | 32 (51.61%) |  |
| Chinna Allapuram | Public Toilets | Children | More than 10 times every month | 13 (14.44%) | 6 (8.45%) | 0.0003 | 6 (42.86%) | 13 (14.44%) | 0.0045 | 6 (42.86%) | 6 (8.45%) | 0.0037 |
|  |  |  | 6-10 times every month | 1 (1.11%) | 9 (12.68%) |  | 2 (14.29%) | 1 (1.11%) |  | 2 (14.29%) | 9 (12.68%) |  |
|  |  |  | 1-5 times every month | 45 (50%) | 19 (26.76%) |  | 4 (28.57%) | 45 (50%) |  | 4 (28.57%) | 19 (26.76%) |  |
|  |  |  | Never | 31 (34.44%) | 37 (52.11%) |  | 2 (14.29%) | 31 (34.44%) |  | 2 (14.29%) | 37 (52.11%) |  |
| Old Town | Raw Produce | Adults | More than 10 times every month | 3 (3%) | 19 (35.19%) | <.0001 | 14 (22.22%) | 3 (3%) | <.0001 | 14 (22.22%) | 19 (35.19%) | 0.1373 |
|  |  |  | 6-10 times every month | 6 (6%) | 13 (24.07%) |  | 10 (15.87%) | 6 (6%) |  | 10 (15.87%) | 13 (24.07%) |  |
|  |  |  | 1-5 times every month | 59 (59%) | 3 (5.56%) |  | 8 (12.7%) | 59 (59%) |  | 8 (12.7%) | 3 (5.56%) |  |
|  |  |  | Never | 32 (32%) | 19 (35.19%) |  | 31 (49.21%) | 32 (32%) |  | 31 (49.21%) | 19 (35.19%) |  |
| Old Town | Raw Produce | Children | More than 10 times every month | 17 (17%) | 20 (25%) | 0.0212* | 6 (35.29%) | 17 (17%) | 0.247 | 6 (35.29%) | 20 (25%) | 0.7035 |
|  |  |  | 6-10 times every month | 10 (10%) | 9 (11.25%) |  | 2 (11.76%) | 10 (10%) |  | 2 (11.76%) | 9 (11.25%) |  |
|  |  |  | 1-5 times every month | 43 (43%) | 17 (21.25%) |  | 4 (23.53%) | 43 (43%) |  | 4 (23.53%) | 17 (21.25%) |  |
|  |  |  | Never | 30 (30%) | 34 (42.5%) |  | 5 (29.41%) | 30 (30%) |  | 5 (29.41%) | 34 (42.5%) |  |
| Chinna Allapuram | Raw Produce | Adults | More than 10 times every month | 1 (1%) | 4 (8%) | 0.0001 | 3 (5.77%) | 1 (1%) | 0.0015 | 3 (5.77%) | 4 (8%) | <.0001 |
|  |  |  | 6-10 times every month | 3 (3%) | 6 (12%) |  | 10 (19.23%) | 3 (3%) |  | 10 (19.23%) | 6 (12%) |  |
|  |  |  | 1-5 times every month | 40 (40%) | 6 (12%) |  | 15 (28.85%) | 40 (40%) |  | 15 (28.85%) | 6 (12%) |  |
|  |  |  | Never | 56 (56%) | 34 (68%) |  | 24 (46.15%) | 56 (56%) |  | 24 (46.15%) | 34 (68%) |  |
| Chinna Allapuram | Raw Produce | Children | More than 10 times every month | 6 (6.06%) | 7 (9.86%) | 0.0089* | 0 (0%) | 6 (6.06%) | 0.2300 | 0 (0%) | 7 (9.86%) | 0.0037 |
|  |  |  | 6-10 times every month | 5 (5.05%) | 10 (14.08%) |  | 2 (18.18%) | 5 (5.05%) |  | 2 (18.18%) | 10 (14.08%) |  |
|  |  |  | 1-5 times every month | 36 (36.36%) | 11 (15.49%) |  | 2 (18.18%) | 36 (36.36%) |  | 2 (18.18%) | 11 (15.49%) |  |
|  |  |  | Never | 52 (52.53%) | 43 (60.56%) |  | 7 (63.64%) | 52 (52.53%) |  | 7 (63.64%) | 43 (60.56%) |  |
