# Supplementary material for: The SaniPath Exposure Assessment Tool: A quantitative approach for assessing exposure to fecal contamination through multiple pathways in low resource urban settlements
Source: PLoS One. 2020 Jun 12;15(6):e0234364. doi: 10.1371/journal.pone.0234364 (PMC7292388; doi:10.1371/journal.pone.0234364)
Supplement: S1 Table — The table depicts results of sampling error analysis using data from the formative study in Accra, Ghana. (DOCX) [file pone.0234364.s004.docx]

# **S3 Table: Variation in *E. coli* concentrations in environmental samples**

Results of sampling error analysis using data from the formative study in Accra, Ghana

| **Neighborhood** | **Pathway** | **Sample Size** | **Mean of Log10 *E. coli* Concentration** | **Standard Deviation of Log10 *E. coli* Concentration** | **Times of sampling** | ***Standard Deviation of Log10 *E. coli* Concentration sample means** |
| --- | --- | --- | --- | --- | --- | --- |
| **Alajo** | Open Drain | 25 | 8.41 | 0.79 | 10,000 | 0.25 |
|  | Drinking Water | 34 | -0.26 | 0.17 | 10,000 | 0.05 |
|  | Public Toilet Swabs | 10 | 2.78 | 1.43 | 10,000 | 0.45 |
|  | Raw Produce | 27 | 2.90 | 1.43 | 10,000 | 0.45 |
|  | Soil | 50 | 2.09 | 1.44 | 10,000 | 0.46 |
| **Bukom** | Open Drain | 19 | 8.95 | 0.98 | 10,000 | 0.31 |
|  | Drinking Water | 23 | -0.12 | 0.38 | 10,000 | 0.12 |
|  | Public Toilet Swab | 24 | 2.01 | 1.35 | 10,000 | 0.43 |
|  | Ocean Water | 19 | 4.20 | 0.74 | 10,000 | 0.23 |
|  | Raw Produce | 19 | 3.19 | 1.53 | 10,000 | 0.48 |
|  | Soil | 27 | 2.57 | 2.11 | 10,000 | 0.67 |
| **Old Fadama** | Open Drain | 22 | 8.12 | 0.87 | 10,000 | 0.27 |
|  | Drinking Water | 31 | 0.34 | 0.95 | 10,000 | 0.30 |
|  | Public Toilet Swabs | 15 | 2.60 | 1.42 | 10,000 | 0.45 |
|  | Raw Produce | 24 | 2.52 | 1.73 | 10,000 | 0.55 |
|  | Soil | 58 | 3.34 | 1.16 | 10,000 | 0.37 |
| **Shiabu** | Open Drain | 24 | 8.75 | 1.27 | 10,000 | 0.40 |
|  | Drinking Water | 29 | -0.17 | 0.45 | 10,000 | 0.14 |
|  | Public Toilet Swabs | 16 | 1.54 | 1.26 | 10,000 | 0.40 |
|  | Ocean Water | 18 | 3.73 | 0.92 | 10,000 | 0.29 |
|  | Raw Produce | 20 | 2.72 | 1.29 | 10,000 | 0.41 |
|  | Soil | 50 | 2.20 | 1.41 | 10,000 | 0.44 |
