# Supplementary material for: The SaniPath Exposure Assessment Tool: A quantitative approach for assessing exposure to fecal contamination through multiple pathways in low resource urban settlements
Source: PLoS One. 2020 Jun 12;15(6):e0234364. doi: 10.1371/journal.pone.0234364 (PMC7292388; doi:10.1371/journal.pone.0234364)
Supplement: S1 Dataset — (ZIP) [file pone.0234364.s005.zip › S1 Dataset/Shiabu Codebook.docx]

| Form | Variable | FRC | Description | Codes |
| --- | --- | --- | --- | --- |
|  | sampeid | F | Sample ID number |  |
|  | samtype | C | Sample type | 1=Drain Water  2=Sand  3=Soil  4=Sediment  5=Swab  6=Ocean water  7=Piped Water  8=Produce |
|  | ev_dd | F | Sample collection date |  |
|  | ev_th | F | Sample collection time |  |
|  | ev_lat | F | Collection point latitude |  |
|  | ev_long | F | Collection point longitude |  |
|  | ev_way | F | Collection waypoint |  |
|  | neighbor | F | neighborhood | 1=Alajo  2=Bukom  3=Old Fadama  4=Shaibu |
|  | ev_notes | F | Notes |  |
| Laboratory | ec_sd | F | Sample processing date |  |
| Laboratory | ec_st | F | Sample processing time |  |
| Laboratory | ec_isd | F | Date placed in incubator |  |
| Laboratory | ec_ist | F | Time placed in incubator |  |
| Laboratory | ec_ied | F | Date removed from incubator |  |
| Laboratory | ec_iet | F | Time removed from incubator |  |
| Laboratory | ec_dil1 | F | Dilution 1 ratio | 1:1  1:10  1:100  1:1000 |
| Laboratory | ec_ecnt1 | F | E. coli count on plate 1 | 998=too dirty to count (TDTC)  999=too numerous to count (TNTC) |
| Laboratory | ec_dil2 | F | Dilution 2 ratio | 1:1  1:10  1:100  1:1000 |
| Laboratory | ec_ecnt2 | F | E. coli count on plate 2 | 998=too dirty to count (TDTC)  999=too numerous to count (TNTC) |
| Laboratory | ec_blank | F | E. coli count on negative control |  |
| Laboratory | dil_one | C | Sample volume plate 1 |  |
| Laboratory | dil_two | C | Sample volume plate 2 |  |
| Laboratory | ec_select | C | Plate selection | 1=use plate 1, neither missing  2=use plate 1, plate 1 zero, plate 2 missing  3=use plate 1, plate 1 TNTC/TDTC, plate 2 missing  4=use plate 2, neither missing  5=use plate 2, plate 2 zero, plate 1 missing  6=use plate 2, plate 2 TNTC/TDTC, plate 1 missing  7=use both, both plates zeros  8=use both, both plates TNTC/TDTC  9=use both, both plates quantifiable |
| Laboratory | ec_conc | C | E coli concentration | Sample types 1,6,7: per 100 mL  Sample types 2,3,4: per gram  Sample type 5: per swab (100 cm^2^)  Sample type 8: per item |
| Laboratory | ec_denom | C | Denominator multiplier |  |
| Drain/Flood | se_sr | F | Type (drain or flood) |  |
| Drain/Flood | se_dhh | F | Drain within 3 m of HH |  |
| Drain/Flood | se_d9hh | F | Drain within 9 m of HH |  |
| Drain/Flood | se_lit | F | Drain water exposed to sunlight |  |
| Drain/Flood | se_f | F | Drain near prepared food |  |
| Drain/Flood | se_fec | F | Drain within 3 m of feces |  |
| Drain/Flood | se_lat | F | Drain within 30 m of latrine or defecation area |  |
| Particulate | pa_lit | F | Particulate sample exposed to sunlight |  |
| Particulate | pa_fec | F | Particulate sample within 3 m of feces |  |
| Particulate | pa_lat | F | Particulate sample within 30 m of latrine or defecation area |  |
| Particulate | pa_type | F | Particulate type | 1=Sediment  2=Soil  3=Sand |
| Particulate | pa_loc | F | Sample collection location |  |
| Particulate | pa_wgt | F | Weight of particulate sample |  |
| Swab | sw_ntype | F | Type of object swabbed | 1= Plastic Container  2= Toilet Seat  3= Latrine Floor  4= Latrine Door Handle  5= Latrine Wall  6= Anal Cleansing Container  7= Latrine Door |
| Swab | sw_to | F | Other type of object swabbed |  |
| Swab | la_type | F | Type of Latrine | 1=traditional pit latrine  2=VIP/KVIP  3=bucket/pan  4=pour flush  5=flush toilet  6=mixed typology  7=other |
| Swab | la_typeo | F | Other type of latrine |  |
| Swab | la_floor | F | Latrine flooring type | 1=wood/natural material  2=cement  3=dirt/unfinished  4=porcelain/tile  5=other |
| Swab | la_flo | F | Other type of latrine flooring |  |
| Swab | la_nstall | F | Number of stalls in latrine block |  |
| Swab | la_vfec | F | Visible feces score for entire latrine block | 1=visible feces in no stalls  2=visible feces in few stalls  3=visible feces in roughly half of stalls  4=visible feces in most stalls  5=visible feces in every stall |
| Swab | la_hw | F | Hand washing station present in latrine | 1=Yes  2=No |
| Swab | la_nuser | F | Daily number of latrine users |  |
| Ocean/Piped/Surface | lw_type | F | Water sample type | 1=Piped water  2=Ocean water  3=Surface water |
| Ocean/Piped/Surface | lo_where | F | Ocean sample location | 1=Open water  2=Inland  3=Other |
| Ocean/Piped/Surface | lo_ot | F | Ocean sample location other |  |
| Ocean/Piped/Surface | ld_source | F | Drinking water source | 1=Public tap  2=Compound/private tap  3=Hand-dug well  4=Tube well/borehole  5=Tanker truck  6=other |
| Ocean/Piped/Surface | ld_hsot | F | Drinking water source other |  |
| Ocean/Piped/Surface | lw_trs | F | Water sample within 3 m of trash |  |
| Ocean/Piped/Surface | lw_fec | F | Water sample within 3 m of feces |  |
| Ocean/Piped/Surface | lw_lat | F | Water sample within 30 m of latrine or defecation area |  |
| Ocean/Piped/Surface | ld_clf | F | Free chlorine residual level (ppm) |  |
| Ocean/Piped/Surface | ld_clt | F | Total chlorine residual level (ppm) |  |
| Produce | fo_ftype | F | Produce type | 1=lettuce  2=tomatoes  3=alasa  4=oranges  5=apples  6=mango  7=spring onion  8=cabbage  9=other |
| Produce | fo_num | F | Number of produce collected |  |
| Produce | fo_fec | F | Produce within 3 m of feces |  |
| Produce | fo_lat | F | Produce within 30 m of latrine or defecation area |  |
| Produce | fo_fly | F | Flies on food |  |
| Produce | fo_sw | F | Produce within 3 m of sewage outfall or open drain |  |
