# Supplementary material for: The SaniPath Exposure Assessment Tool: A quantitative approach for assessing exposure to fecal contamination through multiple pathways in low resource urban settlements
Source: PLoS One. 2020 Jun 12;15(6):e0234364. doi: 10.1371/journal.pone.0234364 (PMC7292388; doi:10.1371/journal.pone.0234364)
Supplement: S1 Dataset — (ZIP) [file pone.0234364.s005.zip › S1 Dataset/Formative Environmental Codebook.doc]

| **Form** | **Variable (Stata)** | **Variable (SAS)** | **NS** | **FRC** | **Description** | **Codes** |
| --- | --- | --- | --- | --- | --- | --- |
|  | locid | locid | n | F | Location ID |  |
|  | locid_lat | locid_la | n | C | Location ID cleaned for latrines |  |
|  | neighb | neighb | n | C | Neighborhood | 1= Alajo  2=Bukom  3=Old Fadama  4=Shiabu |
|  | dom | dom | n | C | Domain | 1=Market  2=HH  3=Public latrine  4=School  5=Sewage  6=Ocean/beach  7=Public drinking  8=Farm  9=Public drain  10=Vendor  11=Open defecation  12=Trash dump  13=Nursery  14=HH survey  15=Flood |
|  | domnum | domnum | n | C | Domain number |  |
|  | obs | obs | n | C | Observation number for that location |  |
|  | barcode | barcode | n | F | Barcode |  |
|  | samtype | samtype | n | C | Sample type | 1=Septage  2=Particulate  3=Large water  4=Small drinking water  5=Small other/environmental water  6=Food  7=Flies  8=Handrinse  9=Swabs |
|  | environ | environ | n | C | Environment | 1=Market  2=HH  3=Public latrine  4=School  5=Sewage  6=Ocean/beach  8=Public drinking  9=Irrigation  10=Public drain  11=Street vendor  12=Public domain  13=Nursery  14=Flood  15=Farms |
|  | samid | samid | n | C | Sample number for that sample type in that environment |  |
|  | neighbor | neighbor | n | R | Neighborhood | 1=Alajo  2=Bukom  3=Old Fadama  4=Shiabu |
|  | ec_samtype | ec_samty | n | C | Another sample type categorization | 1=Septage  2=Particulate  3=Ocean Water (large)  4=Piped Water (large)  5=Other Large Water  6=Sachet  7=Other Stored Water (small)  8=Drain Water  9=Other Small Environmental Water (irrigation and flood)  10=Food  11=Handrinse  12=Swabs  13=Flies |
|  | ev_loc | ev_loc | n | F | Location description |  |
|  | ev_lat | ev_lat | n | C | Latitude (DD) |  |
|  | ev_long | ev_long | n | C | Longitude (DD) |  |
|  | ev_notes | ev_notes | n | F | Notes |  |
|  | ev_dd | ev_dd | s | F | Date |  |
|  | ev_th | ev_th | s | F | Time |  |
| Adenovirus | ad_dateq | ad_dateq | s | F | Date QuantiFast was run |  |
| Adenovirus | ad_qfam | ad_qfam | n | F | QuantiFast FAM |  |
| Adenovirus | ad_qfamd | ad_qfamd | n | F | QuantiFast FAM duplicate run |  |
| Adenovirus | ad_qvic | ad_qvic | n | F | QuantiFast VIC |  |
| Adenovirus | ad_qvicd | ad_qvicd | n | F | QuantiFast VIC duplicate run |  |
| Adenovirus | ad_qintr | ad_qintr | n | C | Interpretation |  |
| Adenovirus | ad_dateo | ad_dateo | s | F | Date OneStep was run |  |
| Adenovirus | ad_ofam5 | ad_ofam3 | n | F | OneStep FAM(5uL) |  |
| Adenovirus | ad_ofam5d | ad_ofam4 | n | F | OneStep FAM(5uL) duplicate run |  |
| Adenovirus | ad_o5qty | ad_o5qty | n | F | OneStep FAM(5uL) quantity (GEC) |  |
| Adenovirus | ad_o5qtyd | ad_o5qt2 | n | F | OneStep FAM(5uL) quantity (GEC) duplicate run |  |
| Adenovirus | ad_o5log | ad_o5log | n | C | OneStep FAM(5uL) log quantity |  |
| Adenovirus | ad_o5logd | ad_o5lo2 | n | C | OneStep FAM(5uL) log quantity duplicate run |  |
| Adenovirus | ad_ofam1 | ad_ofam1 | n | F | OneStep FAM(1:5) |  |
| Adenovirus | ad_ofam1d | ad_ofam2 | n | F | OneStep FAM(1:5) duplicate run |  |
| Adenovirus | ad_o1qty | ad_o1qty | n | F | OneStep FAM(1:5) quantity (GEC) |  |
| Adenovirus | ad_o1qtyd | ad_o1qt2 | n | F | OneStep FAM(1:5) quantity (GEC) duplicate run |  |
| Adenovirus | ad_o1log | ad_o1log | n | C | OneStep FAM(1:5) log quantity |  |
| Adenovirus | ad_o1logd | ad_o1lo2 | n | C | OneStep FAM(1:5) log quantity duplicate run |  |
| Adenovirus | ad_fintr | ad_fintr | n | C | One Step Interpretation |  |
| Adenovirus | ad_oqty | ad_oqty | n | C | One Step quantity |  |
| Adenovirus | ad_oqtyd | ad_oqtyd | n | C | One Step quantity duplicate run |  |
| Adenovirus | ad_ofam | ad_ofam | n | C | One Step FAM |  |
| Adenovirus | ad_ofamd | ad_ofamd | n | C | One Step FAM duplicate run |  |
| Adenovirus | ad_pos | ad_pos | n | C | Sample positive for adenovirus | 1=Positive  2=Negative  3=Inhibition Positive  4=Inhibition Negative |
| Adenovirus | ad_denom | ad_denom | n | C | Denominator, equivalent volume |  |
| Adenovirus | ad_score | ad_score | s | C | reliability score (not ranked) | A1= Ct1>=41, Ct2=no signal  A2= Ct1=no signal, Ct2>=41  B= Ct1<41 and Ct2=no signal OR Ct1=no signal and Ct2<41  C= Ct difference<=4  D= Ct difference>4  E= Ct1=no signal, Ct2=no signal |
| Adenovirus | ad_conc | ad_conc | n | C | Concentration |  |
| Adenovirus | ad_logconc | ad_logco | n | C | Log 10 Concentration |  |
| Beach Conditions | bc_long | bc_long | n | C | Longitude (DD) |  |
| Beach Conditions | bc_lat | bc_lat | n | C | Latitude (DD) |  |
| Beach Conditions | bc_st | bc_st | s | F | Start time for observation |  |
| Beach Conditions | bc_et | bc_et | s | F | End time for observation |  |
| Beach Conditions | bc_date | bc_date | s | F | Date |  |
| Beach Conditions | bc_5yr1 | bc_5yr1 | n | F | Child 1 is under 5 years | 1=Yes  0=No |
| Beach Conditions | bc_12yr1 | bc_12yr1 | n | F | Child 1 is 5-12 years old | 1=Yes  0=No |
| Beach Conditions | bc_st1 | bc_st1 | s | F | Child 1 start time in water |  |
| Beach Conditions | bc_et1 | bc_et1 | s | F | Child 1 end time in water |  |
| Beach Conditions | bc_head1 | bc_head1 | n | F | Child 1 had their head under water | 1=Yes  0=No |
| Beach Conditions | bc_kids1 | bc_kids1 | n | F | Child 1 had contact with another child | 1=Yes  0=No |
| Beach Conditions | bc_sand1 | bc_sand1 | n | F | Child 1 contacted the sand with their hands | 1=Yes  0=No |
| Beach Conditions | bc_toy1 | bc_toy1 | n | F | Child 1 had a toy that contacted the sand | 1=Yes  0=No |
| Beach Conditions | bc_food1 | bc_food1 | n | F | Child 1 had food that contacted the sand | 1=Yes  0=No |
| Beach Conditions | bc_5yr2 | bc_5yr2 | n | F | Child 1 is under 5 years | 1=Yes  0=No |
| Beach Conditions | bc_12yr2 | bc_12yr2 | n | F | Child 1 is 5-12 years old | 1=Yes  0=No |
| Beach Conditions | bc_st2 | bc_st2 | s | F | Child 1 start time in water |  |
| Beach Conditions | bc_et2 | bc_et2 | s | F | Child 1 end time in water |  |
| Beach Conditions | bc_head2 | bc_head2 | n | F | Child 1 had their head under water | 1=Yes  0=No |
| Beach Conditions | bc_kids2 | bc_kids2 | n | F | Child 1 had contact with another child | 1=Yes  0=No |
| Beach Conditions | bc_sand2 | bc_sand2 | n | F | Child 1 contacted the sand with their hands | 1=Yes  0=No |
| Beach Conditions | bc_toy2 | bc_toy2 | n | F | Child 1 had a toy that contacted the sand | 1=Yes  0=No |
| Beach Conditions | bc_food2 | bc_food2 | n | F | Child 1 had food that contacted the sand | 1=Yes  0=No |
| Beach Conditions | bc_5yr3 | bc_5yr3 | n | F | Child 1 is under 5 years | 1=Yes  0=No |
| Beach Conditions | bc_12yr3 | bc_12yr3 | n | F | Child 1 is 5-12 years old | 1=Yes  0=No |
| Beach Conditions | bc_st3 | bc_st3 | s | F | Child 1 start time in water |  |
| Beach Conditions | bc_et3 | bc_et3 | s | F | Child 1 end time in water |  |
| Beach Conditions | bc_head3 | bc_head3 | n | F | Child 1 had their head under water | 1=Yes  0=No |
| Beach Conditions | bc_kids3 | bc_kids3 | n | F | Child 1 had contact with another child | 1=Yes  0=No |
| Beach Conditions | bc_sand3 | bc_sand3 | n | F | Child 1 contacted the sand with their hands | 1=Yes  0=No |
| Beach Conditions | bc_toy3 | bc_toy3 | n | F | Child 1 had a toy that contacted the sand | 1=Yes  0=No |
| Beach Conditions | bc_food3 | bc_food3 | n | F | Child 1 had food that contacted the sand | 1=Yes  0=No |
| Beach Conditions | bc_adw | bc_adw | n | F | Number of adults defecating in the water |  |
| Beach Conditions | bc_adb | bc_adb | n | F | Number of adults defecating on the beach |  |
| Beach Conditions | bc_12iw | bc_12iw | n | F | Number of children 5-12 years in the water |  |
| Beach Conditions | bc_12eat | bc_12eat | n | F | Number of children 5-12 years eating |  |
| Beach Conditions | bc_12dw | bc_12dw | n | F | Number of children 5-12 years defecating in the water |  |
| Beach Conditions | bc_12db | bc_12db | n | F | Number of children 5-12 years defecating on the beach |  |
| Beach Conditions | bc_5iw | bc_5iw | n | F | Number of children under 5 in the water |  |
| Beach Conditions | bc_5eat | bc_5eat | n | F | Number of children under 5 eating |  |
| Beach Conditions | bc_5dw | bc_5dw | n | F | Number of children under 5 defecating in the water |  |
| Beach Conditions | bc_5db | bc_5db | n | F | Number of children under 5 defecating on the beach |  |
| Beach Conditions | bc_anip | bc_anip | n | F | Number of pigs on the beach |  |
| Beach Conditions | bc_anic | bc_anic | n | F | Number of chickens on the beach |  |
| Beach Conditions | bc_anid | bc_anid | n | F | Number of dogs on the beach |  |
| Beach Conditions | bc_anig | bc_anig | n | F | Number of goats on the beach |  |
| Beach Conditions | bc_anio | bc_anio | s | F | Other animals on the beach |  |
| Beach Conditions | bc_latp | bc_latp | n | F | Number of public latrines on the beach |  |
| Beach Conditions | bc_lath | bc_lath | n | F | Number of HH latrines on the beach |  |
| Child Behavior | be_long | be_long | n | C | Longitude (DD) |  |
| Child Behavior | be_lat | be_lat | n | C | Latitude (DD) |  |
| Child Behavior | be_st | be_st | s | F | Start time for observation |  |
| Child Behavior | be_et | be_et | s | F | End time for observation |  |
| Child Behavior | be_date | be_date | s | F | Date |  |
| Child Behavior | cg_cgid | cg_cgid | s | F | Caregiver ID |  |
| Child Behavior | be_cgage | be_cgage | n | F | Caregiver age | 1= <12 years  2= ≥12 years |
| Child Behavior | be_cgsex | be_cgsex | n | F | Sex of caregiver | 1=Male  2=Female |
| Child Behavior | gb_st | gb_st | s | F | Start time for caregiver's behavior |  |
| Child Behavior | gb_hw | gb_hw | n | F | Caregiver handwashes | 1=Yes  0=No |
| Child Behavior | gb_hw1 | gb_hw1 | n | F | Caregiver uses soap when handwashing | 1=With soap  2=Without soap |
| Child Behavior | gb_pf | gb_pf | n | F | Caregiver prepares food | 1=Yes  0=No |
| Child Behavior | gb_sd | gb_sd | n | F | Caregiver disposes of sullage | 1=Yes  0=No |
| Child Behavior | gb_la | gb_la | n | F | Caregiver uses the latrine | 1=Yes  0=No |
| Child Behavior | gb_hm | gb_hm | n | F | Caregiver handles money | 1=Yes  0=No |
| Child Behavior | gb_sw | gb_sw | n | F | Caregiver sweeps | 1=Yes  0=No |
| Child Behavior | be_notes | be_notes | n | F | Notes |  |
| Child Behavior | chid | chid | s | R | Child ID |  |
| Child Behavior | be_cage | be_cage | n | F | Child age | 1= <1 year  2=1-2 years  3= >2 years |
| Child Behavior | be_sex | be_sex | n | F | Sex of child | 1=Male  2=Female |
| Child Behavior | be_mob | be_mob | s | F | Child's mobility | 1=Crawl  2=Walk  3=Sit independently |
| Child Behavior | be_moba | be_moba | n | C | Crawl, from be_mob | 1=Yes  0=No |
| Child Behavior | be_mobb | be_mobb | n | C | Walk, from be_mob | 1=Yes  0=No |
| Child Behavior | be_mobc | be_mobc | n | C | Sit independently, from be_mob | 1=Yes  0=No |
| Child Behavior | be_teeth | be_teeth | n | F | Child is teething | 1=Yes  2=No  3=NA |
| Child Behavior | be_area | be_area | n | F | Child location | 1=Unimproved/dirt  2=Improved/floor  3=Off the ground  4=In SWATA  5=In drain  6=Out of view |
| Child Behavior | be_hands | be_hands | n | F | Child is touching the locations with their hands | 1=Yes  0=No |
| Child Behavior | be_shoes | be_shoes | n | F | Child is wearing shoes | 1=Yes  2=No  3=NA |
| Child Behavior | be_stc | be_stc | s | F | Start time for child's behavior |  |
| Child Behavior | be_play | be_play | n | F | Child is playing or sitting | 1=Yes  0=No |
| Child Behavior | be_sleep | be_sleep | n | F | Child is sleeping | 1=Yes  0=No |
| Child Behavior | be_chw | be_chw | n | F | Child handwashes | 1=Yes  0=No |
| Child Behavior | be_chw1 | be_chw1 | n | F | Child handwashes with soap | 1=With soap  2=Without soap |
| Child Behavior | be_cbt | be_cbt | n | F | Child is taking a bath | 1=Yes  0=No |
| Child Behavior | be_cbs | be_cbs | n | F | Child is taking a bath with soap | 1=With soap  2=Without soap |
| Child Behavior | be_cdf | be_cdf | n | F | Child defecates | 1=Yes  0=No |
| Child Behavior | be_cdf1 | be_cdf1 | n | F | Location where child defecates |  |
| Child Behavior | be_cdf2 | be_cdf2 | n | F | Child's bottom is cleaned after he/she defecates | 1=Cleaned  2=Not cleaned |
| Child Behavior | be_cdf3 | be_cdf3 | n | F | Location where child's feces are disposed of | 1=Left on ground  2=Drain  3=Trash  4=Nothing  5=Latrine |
| Child Behavior | be_ce | be_ce | n | F | Child is eating | 1=Yes  0=No |
| Child Behavior | be_ce1 | be_ce1 | n | F | Type of food child is eating | 1=Prepared at home  2=Purchased food  3=Raw produce  4=DK |
| Child Behavior | be_ce2 | be_ce2 | n | F | How child is eating | 1=Cutlery  2=Hands |
| Child Behavior | be_cchld | be_cchld | n | F | Number of times child contacts another child |  |
| Child Behavior | be_cadlt | be_cadlt | n | F | Number of times child has contact with an adult |  |
| Child Behavior | be_fmth | be_fmth | n | F | Number of times child places fingers in their mouth |  |
| Child Behavior | be_omth | be_omth | n | F | Number of times child places objects in their mouth |  |
| Child Behavior | be_omthl | be_omthl | s | F | List of objects child places in their mouth |  |
| Cryptosporidium | cr_dateq | cr_dateq | s | F | Date QuantiFast was run |  |
| Cryptosporidium | cr_qfam | cr_qfam | n | F | QuantiFast FAM |  |
| Cryptosporidium | cr_qvic | cr_qvic | n | F | QuantiFast VIC |  |
| Cryptosporidium | cr_qintr | cr_qintr | s | C | Interpretation |  |
| Cryptosporidium | cr_dateo | cr_dateo | s | F | Date OneStep was run |  |
| Cryptosporidium | cr_ofam5 | cr_ofam5 | n | F | OneStep FAM(5uL) |  |
| Cryptosporidium | cr_o5qty | cr_o5qty | n | F | OneStep FAM(5uL) quantity (GEC) |  |
| Cryptosporidium | cr_o5log | cr_o5log | n | C | OneStep FAM(5uL) log quantity |  |
| Cryptosporidium | cr_ofam1 | cr_ofam1 | n | F | OneStep FAM(1:5) |  |
| Cryptosporidium | cr_o1qty | cr_o1qty | n | F | OneStep FAM(1:5) quantity (GEC) |  |
| Cryptosporidium | cr_o1log | cr_o1log | n | C | OneStep FAM(1:5) log quantity |  |
| Cryptosporidium | cr_fintr | cr_fintr | s | C | Final interpretation |  |
| Drain Conditions | dc_date | dc_date | s | F | Date |  |
| Drain Conditions | dc_st | dc_st | s | F | Observation start time |  |
| Drain Conditions | dc_et | dc_et | s | F | Observation end time |  |
| Drain Conditions | dc_lat1 | dc_lat1 | n | C | GPS latitude for drain 1 |  |
| Drain Conditions | dc_long1 | dc_long1 | n | C | GPS longitude for drain 1 |  |
| Drain Conditions | dc_a1 | dc_a1 | n | F | Drain construction type for drain 1 | 1=Planned/cemented  2=Unplanned/dirt ditch |
| Drain Conditions | dc_b1 | dc_b1 | s | F | Environment around drain (within 2m radius) for drain 1 | 1=Schools (nursery and primary)  2=Households  3=Businesses  4=Market/food vendors  5=Urban agriculture sites  6=Latrine/bathing facilities  7=Other |
| Drain Conditions | dc_b1a | dc_b1a | n | C | Schools, from dc_b1 | 1=Yes  0=No |
| Drain Conditions | dc_b1b | dc_b1b | n | C | Households, from dc_b1 | 1=Yes  0=No |
| Drain Conditions | dc_b1c | dc_b1c | n | C | Businesses, from dc_b1 | 1=Yes  0=No |
| Drain Conditions | dc_b1d | dc_b1d | n | C | Market/food vendors, from dc_b1 | 1=Yes  0=No |
| Drain Conditions | dc_b1e | dc_b1e | n | C | Urban agriculture sites, from dc_b1 | 1=Yes  0=No |
| Drain Conditions | dc_b1f | dc_b1f | n | C | Latrine/bathing facilities, from dc_b1 | 1=Yes  0=No |
| Drain Conditions | dc_c1 | dc_c1 | n | F | Water level of the drain at observation time for drain 1 | 1=Low  2=Medium  3=High |
| Drain Conditions | dc_d1 | dc_d1 | n | F | Water in the drain is stagnant or moving for drain 1 | 1=Stagnant  2=Moving |
| Drain Conditions | dc_e1 | dc_e1 | n | F | Number of children under 5 inside the drain for drain 1 |  |
| Drain Conditions | dc_f1 | dc_f1 | n | F | Number of children 5-12 inside the drain for drain 1 |  |
| Drain Conditions | dc_g1 | dc_g1 | n | F | Number of adults (12+) seen defecating into/inside the drain for drain 1 |  |
| Drain Conditions | dc_h1 | dc_h1 | n | F | Number of children under 5 seen defecating into/inside the drain for drain 1 |  |
| Drain Conditions | dc_i1 | dc_i1 | n | F | Number of children 5-12 seen defecating into/inside the drain for drain 1 |  |
| Drain Conditions | dc_j1 | dc_j1 | n | F | Number dumping into drain (sullage, potty contents, ploy bags) for drain 1 |  |
| Drain Conditions | dc_lat2 | dc_lat2 | n | C | GPS latitude for drain 2 |  |
| Drain Conditions | dc_long2 | dc_long2 | n | C | GPS longitude for drain 2 |  |
| Drain Conditions | dc_a2 | dc_a2 | n | F | Drain construction type for drain 2 | 1=Planned/cemented  2=Unplanned/dirt ditch |
| Drain Conditions | dc_b2 | dc_b2 | s | F | Environment around drain (within 2m radius) for drain 2 | 1=Schools (nursery and primary)  2=Households  3=Businesses  4=Market/food vendors  5=Urban agriculture sites  6=Latrine/bathing facilities  7=Other |
| Drain Conditions | dc_b2a | dc_b2a | n | C | Schools, from dc_b2 | 1=Yes  0=No |
| Drain Conditions | dc_b2b | dc_b2b | n | C | Households, from dc_b2 | 1=Yes  0=No |
| Drain Conditions | dc_b2c | dc_b2c | n | C | Businesses, from dc_b2 | 1=Yes  0=No |
| Drain Conditions | dc_b2d | dc_b2d | n | C | Market/food vendors, from dc_b2 | 1=Yes  0=No |
| Drain Conditions | dc_b2e | dc_b2e | n | C | Urban agriculture sites, from dc_b2 | 1=Yes  0=No |
| Drain Conditions | dc_b2f | dc_b2f | n | C | Latrine/bathing facilities, from dc_b2 | 1=Yes  0=No |
| Drain Conditions | dc_c2 | dc_c2 | n | F | Water level of the drain at observation time for drain 2 | 1=Low  2=Medium  3=High |
| Drain Conditions | dc_d2 | dc_d2 | n | F | Water in the drain is stagnant or moving for drain 2 | 1=Stagnant  2=Moving |
| Drain Conditions | dc_e2 | dc_e2 | n | F | Number of children under 5 inside the drain for drain 2 |  |
| Drain Conditions | dc_f2 | dc_f2 | n | F | Number of children 5-12 inside the drain for drain 2 |  |
| Drain Conditions | dc_g2 | dc_g2 | n | F | Number of adults (12+) seen defecating into/inside the drain for drain 2 |  |
| Drain Conditions | dc_h2 | dc_h2 | n | F | Number of children under 5 seen defecating into/inside the drain for drain 2 |  |
| Drain Conditions | dc_i2 | dc_i2 | n | F | Number of children 5-12 seen defecating into/inside the drain for drain 2 |  |
| Drain Conditions | dc_j2 | dc_j2 | n | F | Number dumping into drain (sullage, potty contents, ploy bags) for drain 2 |  |
| Drain Conditions | dc_lat3 | dc_lat3 | n | C | GPS latitude for drain 3 |  |
| Drain Conditions | dc_long3 | dc_long3 | n | C | GPS longitude for drain 3 |  |
| Drain Conditions | dc_a3 | dc_a3 | n | F | Drain construction type for drain 3 | 1=Planned/cemented  2=Unplanned/dirt ditch |
| Drain Conditions | dc_b3 | dc_b3 | s | F | Environment around drain (within 2m radius) for drain 3 | 1=Schools (nursery and primary)  2=Households  3=Businesses  4=Market/food vendors  5=Urban agriculture sites  6=Latrine/bathing facilities  7=Other |
| Drain Conditions | dc_b3a | dc_b3a | n | C | Schools, from dc_b3 | 1=Yes  0=No |
| Drain Conditions | dc_b3b | dc_b3b | n | C | Households, from dc_b3 | 1=Yes  0=No |
| Drain Conditions | dc_b3c | dc_b3c | n | C | Businesses, from dc_b3 | 1=Yes  0=No |
| Drain Conditions | dc_b3d | dc_b3d | n | C | Market/food vendors, from dc_b3 | 1=Yes  0=No |
| Drain Conditions | dc_b3e | dc_b3e | n | C | Urban agriculture sites, from dc_b3 | 1=Yes  0=No |
| Drain Conditions | dc_b3f | dc_b3f | n | C | Latrine/bathing facilities, from dc_b3 | 1=Yes  0=No |
| Drain Conditions | dc_c3 | dc_c3 | n | F | Water level of the drain at observation time for drain 3 | 1=Low  2=Medium  3=High |
| Drain Conditions | dc_d3 | dc_d3 | n | F | Water in the drain is stagnant or moving for drain 3 | 1=Stagnant  2=Moving |
| Drain Conditions | dc_e3 | dc_e3 | n | F | Number of children under 5 inside the drain for drain 3 |  |
| Drain Conditions | dc_f3 | dc_f3 | n | F | Number of children 5-12 inside the drain for drain 3 |  |
| Drain Conditions | dc_g3 | dc_g3 | n | F | Number of adults (12+) seen defecating into/inside the drain for drain 3 |  |
| Drain Conditions | dc_h3 | dc_h3 | n | F | Number of children under 5 seen defecating into/inside the drain for drain 3 |  |
| Drain Conditions | dc_i3 | dc_i3 | n | F | Number of children 5-12 seen defecating into/inside the drain for drain 3 |  |
| Drain Conditions | dc_j3 | dc_j3 | n | F | Number dumping into drain (sullage, potty contents, ploy bags) for drain 3 |  |
| Drain Conditions | dc_notes | dc_notes | s | F | Notes |  |
| Drain Tool | d_date | d_date | s | F | Date |  |
| Drain Tool | d_lat | d_lat | n | F | Latitude (DD) |  |
| Drain Tool | d_long | d_long | n | F | Longitude (DD) |  |
| Drain Tool | d_neigh | d_neigh | n | F | Neighborhood | 1= Alajo  2=Bukom  3=Old Fadama  4=Shiabu |
| Drain Tool | d_time | d_time | s | F | Time |  |
| Drain Tool | d_size | d_size | n | F | Drain size | 1=Small  2=Medium  3=Large  4=Extra large |
| Drain Tool | d_st1 | d_st1 | n | F | Drain located on one side of the street | 1=Yes  0=No |
| Drain Tool | d_st2 | d_st2 | n | F | Drain located on both sides of the street | 1=Yes  0=No |
| Drain Tool | d_bt | d_bt | n | F | Drain located between buildings | 1=Yes  0=No |
| Drain Tool | d_sea | d_sea | n | F | Drain located terminal to the sea | 1=Yes  0=No |
| Drain Tool | d_out | d_out | n | F | Drain located terminal outside the neighborhood | 1=Yes  0=No |
| Drain Tool | d_bran | d_bran | n | F | Branching point | 1=Yes  0=No |
| Drain Tool | d_merg | d_merg | n | F | Merging point | 1=Yes  0=No |
| Drain Tool | d_rc | d_rc | n | F | Round corner | 1=Yes  0=No |
| Drain Tool | d_90c | d_90c | n | F | 90° corner | 1=Yes  0=No |
| Drain Tool | d_school | d_school | n | F | Schools around drain (within 2m radius) | 1=Yes  0=No |
| Drain Tool | d_home | d_home | n | F | Homes around drain (within 2m radius) | 1=Yes  0=No |
| Drain Tool | d_bus | d_bus | n | F | Businesses around drain (within 2m radius) | 1=Yes  0=No |
| Drain Tool | d_ag | d_ag | n | F | Agriculture around drain (within 2m radius) | 1=Yes  0=No |
| Drain Tool | d_ven | d_ven | n | F | Vendor stand around drain (within 2m radius) | 1=Yes  0=No |
| Drain Tool | d_pipe | d_pipe | n | F | Water pipes around drain (within 2m radius) | 1=Yes  0=No |
| Drain Tool | d_latr | d_latr | n | F | Latrine around drain (within 2m radius) | 1=Yes  0=No |
| Drain Tool | d_bath | d_bath | n | F | Bathing facility around drain (within 2m radius) | 1=Yes  0=No |
| Drain Tool | d_level | d_level | n | F | Water level in drain | 1=Dry  2=Low  3=Medium  4=High  5=Unable to see |
| Drain Tool | d_move | d_move | n | F | Water is stagnant/moving | 1=Stagnant  2=Moving  3=NA |
| Drain Tool | d_direct | d_direct | n | F | If moving, direction of flow | 1=N  2=NE  3=E  4=SE  5=S  6=SW  7=W  8=NW  9=NA |
| Drain Tool | d_mdespt | d_mdespt | s | F | Description of flow |  |
| Drain Tool | d_ncov | d_ncov | n | F | Drain uncovered | 1=Yes  0=No |
| Drain Tool | d_ctycov | d_ctycov | n | F | Covered by city | 1=Yes  0=No |
| Drain Tool | d_ctzcov | d_ctzcov | n | F | Covered by citizens | 1=Yes  0=No |
| Drain Tool | d_uncov | d_uncov | n | F | Covered by unknown source | 1=Yes  0=No |
| Drain Tool | d_ctyscale | d_ctyscale | n | F | Scale of coverage by city | 0=Uncovered  1=Few areas  2=50%  3=75%  4=Almost all  5=Completely covered |
| Drain Tool | d_ctzscale | d_ctzscale | n | F | Scale of coverage by citizens | 0=Uncovered  1=Few areas  2=50%  3=75%  4=Almost all  5=Completely covered |
| Drain Tool | d_constfcty | d_constfcty | n | F | Formal drain construction by city | 1=Yes  0=No |
| Drain Tool | d_constfctz | d_constfctz | n | F | Formal drain construction by citizens | 1=Yes  0=No |
| Drain Tool | d_consteco | d_consteco | n | F | Ecological drain construction | 1=Yes  0=No |
| Drain Tool | d_line | d_line | n | F | Drain is cement/dirt lined | 1=Cement  2=Stones  3=Dirt  4=Mixed |
| E coli and TC | ec_notes | ec_notes | s | F | Notes |  |
| E coli and TC | ec_sd | ec_sd | s | F | Date sample processing began |  |
| E coli and TC | ec_isd | ec_isd | s | F | Date sample is placed in incubator |  |
| E coli and TC | ec_ied | ec_ied | s | F | Date sample is taken from incubator |  |
| E coli and TC | ec_st | ec_st | s | F | Time sample processing began |  |
| E coli and TC | ec_ist | ec_ist | s | F | Time sample is placed in incubator |  |
| E coli and TC | ec_iet | ec_iet | s | F | Time sample is taken from incubator |  |
| E coli and TC | ec_dil1 | ec_dil1 | n | F | Dilution 1 |  |
| E coli and TC | ec_ecnt1 | ec_ecnt1 | n | F | E coli cfu for dilution 1 | 998= Too dirty to count  999=TNTC |
| E coli and TC | ec_d1tc | ec_d1tc | n | F | TC present for dilution 1 | 1=TC present  0=TC not present |
| E coli and TC | ec_dil2 | ec_dil2 | n | F | Dilution 2 |  |
| E coli and TC | ec_ecnt2 | ec_ecnt2 | n | F | E coli cfu for dilution 2 | 998= Too dirty to count  999=TNTC |
| E coli and TC | ec_d2tc | ec_d2tc | n | F | TC present for dilution 2 | 1=TC present  0=TC not present |
| E coli and TC | ec_dil3 | ec_dil3 | n | F | Dilution 3 |  |
| E coli and TC | ec_ecnt3 | ec_ecnt3 | n | F | E coli cfu for dilution 3 | 998= Too dirty to count  999=TNTC |
| E coli and TC | ec_d3tc | ec_d3tc | n | F | TC present for dilution 3 | 1=TC present  0=TC not present |
| E coli and TC | ec_ecnt1b | ec_ecnt4 | n | F | E coli cfu for dilution 1, duplicate | 998= Too dirty to count  999=TNTC |
| E coli and TC | ec_d1tc2 | ec_d1tc2 | n | F | TC present for dilution 1, duplicate | 1=TC present  0=TC not present |
| E coli and TC | ec_ecnt2b | ec_ecnt5 | n | F | E coli cfu for dilution 2, duplicate | 998= Too dirty to count  999=TNTC |
| E coli and TC | ec_d2tc2 | ec_d2tc2 | n | F | TC present for dilution 2, duplicate | 1=TC present  0=TC not present |
| E coli and TC | ec_ecnt3b | ec_ecnt6 | n | F | E coli cfu for dilution 3, duplicate | 998= Too dirty to count  999=TNTC |
| E coli and TC | ec_d3tc2 | ec_d3tc2 | n | F | TC present for dilution 3, duplicate | 1=TC present  0=TC not present |
| E coli and TC | ec_ecntb | ec_ecntb | n | F | E coli cfu for blank | 998= Too dirty to count  999=TNTC |
| E coli and TC | ec_denom | ec_denom | n | C | Denominator, equivalent volume |  |
| E coli and TC | ec_score | ec_score | s | C | reliability score (not ranked) | B= All 3 plates with counts equal to 0  C= All 3 plates with counts over 200  D= Plate 1<20, Plate 2<20 or TNTC/TDTC, Plate 3<20 or TNTC/TDTC  A= all other combinations, where there was E coli data |
| E coli and TC | ec_select | ec_selec | n | C | determination of which plates to use for calculating concentration | 1= plate 1  2= plate 2  3= plate 3  12= plates 1 and 2  23= plates 2 and 3  123= plates 1, 2, and 3 |
| E coli and TC | ec_conc | ec_conc | n | C | Concentration |  |
| E coli and TC | ec_lnconc | ec_lncon | n | C | Log 10 concentration |  |
| E coli and TC | ec_pos | ec_pos | n | C | Sample positive for *E. coli* | 1=Positive  2=Negative |
| Enterococci | en_pocd | en_pocd | s | F | Date sample processing began |  |
| Enterococci | en_incd1 | en_incd1 | s | F | Date sample is placed in incubator |  |
| Enterococci | en_incd2 | en_incd2 | s | F | Date sample is taken from incubator |  |
| Enterococci | en_poct | en_poct | s | F | Time sample processing began |  |
| Enterococci | en_inct1 | en_inct1 | s | F | Time sample is placed in incubator |  |
| Enterococci | en_inct2 | en_inct2 | s | F | Time sample is taken from incubator |  |
| Enterococci | en_dil1 | en_dil1 | n | F | Dilution 1 |  |
| Enterococci | en_cnt1 | en_cnt1 | n | F | Enterococci cfu for dilution 1 | 998= Too dirty to count  999=TNTC |
| Enterococci | en_cnt1b | en_cnt1b | n | F | Enterococci cfu for dilution 1, duplicate | 998= Too dirty to count  999=TNTC |
| Enterococci | en_dil2 | en_dil2 | n | F | Dilution 2 |  |
| Enterococci | en_cnt2 | en_cnt2 | n | F | Enterococci cfu for dilution 2 | 998= Too dirty to count  999=TNTC |
| Enterococci | en_cnt2b | en_cnt2b | n | F | Enterococci cfu for dilution 2, duplicate | 998= Too dirty to count  999=TNTC |
| Enterococci | en_dil3 | en_dil3 | n | F | Dilution 3 |  |
| Enterococci | en_cnt3 | en_cnt3 | n | F | Enterococci cfu for dilution 3 | 998= Too dirty to count  999=TNTC |
| Enterococci | en_cnt3b | en_cnt3b | n | F | Enterococci cfu for dilution 3, duplicate | 998= Too dirty to count  999=TNTC |
| Enterococci | en_cntb | en_cntb | n | F | Enterococci cfu for blank | 998= Too dirty to count  999=TNTC |
| Enterococci | en_denom | en_denom | n | C | Denominator, equivalent volume |  |
| Enterococci | en_score | en_score | s | C | reliability score (not ranked) | B= All 3 plates with counts equal to 0  C= All 3 plates with counts over 200  D= Plate 1<20, Plate 2<20 or TNTC/TDTC, Plate 3<20 or TNTC/TDTC  A= all other combinations, where there was entero data |
| Enterococci | en_select | en_selec | n | C | determination of which plates to use for calculating concentration | 1= plate 1  2= plate 2  3= plate 3  12= plates 1 and 2  23= plates 2 and 3  123= plates 1, 2, and 3 |
| Enterococci | en_conc | en_conc | n | C | Concentration |  |
| Enterococci | en_lnconc | en_lncon | n | C | Log 10 Concentration |  |
| Flies | fl_tst | fl_tst | s | F | Trap set up time |  |
| Flies | fl_tet | fl_tet | s | F | Trap take down time |  |
| Flies | fl_ttt | fl_ttt | n | F | Total trap time |  |
| Flies | fl_loc | fl_loc | n | F | Location of trap | 1=Drain  2=Latrine  3=School  4=Household  5=Market |
| Flies | fl_trap | fl_trap | n | F | Trap number | 1=Negative control |
| Flies | fl_nfl | fl_nfl | n | F | Number of flies |  |
| Flies | fl_ants | fl_ants | n | F | Ants present | 1=Yes  2=No |
| Flies | fl_notes | fl_notes | n | F | Notes |  |
| Flood | fa2_dd | fa2_dd | s | F | Date |  |
| Flood | fa2_t | fa2_t | s | F | Time |  |
| Flood | fa2_lat | fa2_lat | n | C | Latitude (DD) |  |
| Flood | fa2_long | fa2_long | n | C | Longitude (DD) |  |
| Flood | fa2_1 | fa2_1 | n | F | Children are in the area | 1=Yes  2=No |
| Flood | fa2_2 | fa2_2 | n | F | People working to remove water | 1=Yes  2=No |
| Flood | fa2_3 | fa2_3 | n | F | People eating in flood water | 1=Yes  2=No |
| Flood | fa2_4 | fa2_4 | n | F | Businesses open as usual | 1=Yes  2=No |
| Flood | fa2_5 | fa2_5 | s | F | Other activities occurring in flood area |  |
| Flood | fa2_6 | fa2_6 | n | F | Observation of dumping trash in drains | 1=Yes  2=No  3=NA |
| Flood | fa2_7 | fa2_7 | n | F | Observation of dumping trash in flood water | 1=Yes  2=No |
| Flood | fa2_8 | fa2_8 | n | F | Observation of open defecation | 1=Yes  2=No |
| Flood | fa2_notes | fa2_note | s | F | Notes for fa2_2- fa2_8 |  |
| Flood | fb2_1 | fb2_1 | n | F | Impervious surface | 1=All concrete (0% dirt)  2=Mostly concrete (25% dirt)  3=Half concrete (50% dirt)  4=Mostly dirt (75% dirt)  5=All dirt (100% dirt)  6=Sand |
| Flood | fb2_2 | fb2_2 | n | F | Water marks | 1=Yes  2=No |
| Flood | fb2_3 | fb2_3 | n | F | Presence of standing water | 1=Yes  2=No |
| Flood | fb2_4 | fb2_4 | n | F | Preventive factors | 1=Hand dug drains  2=Sand bags  3=Tires  4=Sawdust  5=Elevated houses  6=Formal drain  7=Other  8=None |
| Flood | fb2_4o | fb2_4o | s | F | Other preventive factors |  |
| Flood | fb2_5 | fb2_5 | n | F | Presence of drains | 1=Yes  2=No |
| Flood | fb2_6 | fb2_6 | n | F | Drain obviously clogged | 1=Yes  2=No  3=NA |
| Flood | fb2_notes | fb2_note | s | F | Notes for fb2_1- fb2_6 and fa2_1 |  |
| Flood Before | fb_dd | fb_dd | s | F | Date |  |
| Flood Before | fb_t | fb_t | s | F | Time |  |
| Flood Before | fb_mo | fb_mo | n | F | Observation | 1=Yes  0=No |
| Flood Before | fb_l | fb_l | n | F | Respondent was liaison | 1=Yes  0=No |
| Flood Before | fb_r | fb_r | n | F | Respondent was resident | 1=Yes  0=No |
| Flood Before | fb_1 | fb_1 | n | F | Cause of flooding | 1=Drain overflowing  2=Water cannot reach drain  3=No drain  4=Low lying area  5=Other |
| Flood Before | fb_2 | fb_2 | s | F | Other cause of flooding |  |
| Flood Before | fb_3 | fb_3 | n | F | Why drain overflows | 1=Clogged drain  2=Too much water  3=Other |
| Flood Before | fb_4 | fb_4 | s | F | Other reason drain overflows |  |
| Flood Before | fb_notes | fb_notes | s | F | Notes |  |
| Flood Survey | fa1_dd | fa1_dd | s | F | Date interview was conducted |  |
| Flood Survey | fa1_t | fa1_t | s | F | Time interview was conducted |  |
| Flood Survey | fa1_int | fa1_int | s | F | Respondent |  |
| Flood Survey | fa1_1 | fa1_1 | n | F | Cause of flooding | 1=Overflowing of drain  2=Water can't reach drain  3=No drain  4=Low lying area  5=Other |
| Flood Survey | fa1_2 | fa1_2 | s | F | Other cause of flooding |  |
| Flood Survey | fa1_1notes | fa1_1notes | s | F | Notes for fa1_1 and fa1_2 |  |
| Flood Survey | fa1_3 | fa1_3 | n | F | Respondent tries to prevent flooding in home | 1=Yes  2=No |
| Flood Survey | fa1_3a1 | fa1_3a1 | n | F | How respondent tries to prevent flooding in home | 1=Digging drains  2=Placement of sandbags  3=Placement of tires  4=Placement of sawdust  5=Raise building structures  6=Other |
| Flood Survey | fa1_3ao | fa1_3ao | s | F | Other way respondent tries to prevent flooding in home |  |
| Flood Survey | fa1_3b | fa1_3b | s | F | Reason respondent does not try to prevent flooding in home |  |
| Flood Survey | fa1_4 | fa1_4 | n | F | Number of days flooding lasts in respondent's area |  |
| Flood Survey | fa1_5 | fa1_5 | n | F | Respondent removes flood water from home | 1=Yes  2=No |
| Flood Survey | fa1_5a | fa1_5a | n | F | How respondent removes flood water from home | 1=Scoop water out of home  2=Other |
| Flood Survey | fa1_5ao | fa1_5ao | s | F | Other way respondent removes flood water from home |  |
| Flood Survey | fa1_5b | fa1_5b | s | F | Reason respondent does not remove flood water from home |  |
| Flood Survey | fa1_6 | fa1_6 | n | F | Number of times per year house floods |  |
| Flood Survey | fa1_6notes | fa1_6notes | s | F | Notes for fa1_4 and fa1_5 |  |
| Flood Survey | fa1_7 | fa1_7 | n | F | Respondent stays in home after flooding | 1=Yes  2=No  3=Sometimes  4=No answer |
| Flood Survey | fa1_8 | fa1_8 | n | F | Children stay in home after flooding | 1=Yes  2=No  3=Sometimes  4=No answer  5=NA |
| Flood Survey | fa1_8notes | fa1_8notes | s | F | Notes for fa1_6-fa1_8 |  |
| Flood Survey | fa1_9 | fa1_9 | n | F | Frequency respondent sees feces dumped in drain | 1=Always (everyday)  2=Often (every other day)  3=Sometimes (once a week)  4=Never  5=NA |
| Flood Survey | fa1_10 | fa1_10 | n | F | Seasonal variation (rainy v. dry) in feces dumped in drain | 1=More often  2=Less often  3=Same  4=Never  5=NA |
| Flood Survey | fa1_notes | fa1_notes | s | F | Notes |  |
| Flood Survey | neighbor | neighbor | s | F | Neighborhood |  |
| Flood Survey | fb_3 | fb_3 | n | F | Reason drain overflows | 1=Clogged drain  2=Too much water  3=Other |
| Flood Survey | fb_4 | fb_4 | s | F | Other reason drain overflows |  |
| Food | fo_fly | fo_fly | n | F | Sample was taken with flies on food | 1=Yes  0=No |
| Food | fo_who | fo_who | n | R | Food server | 1=Caretaker  2=School feeding program  3=Vendor  4=Other |
| Food | fo_mobile | fo_mobil | n | R | Type of vendor | 1=Mobile  2=Non=mobile |
| Food | fo_ftype | fo_ftype | n | R | Type of food collected | 1=Lettuce  2=Tomato  3=Alasa  4=Orange  5=Apple  6=Mango  7=Chili pepper  8=Spring onion  9=Cabbage  10=Prepared food  11=Other |
| Food | fo_typ | fo_typ | s | F | Name of food if prepared or other |  |
| Food | fo_num | fo_num | n | F | Number collected |  |
| Food | fo_serv | fo_serv | n | R | How food placed in whirl-pak bag | 1=Pastic/paper  2=Hands  3=Utensil or dish  4=Other |
| Food | fo_o | fo_o | s | R | Other way food placed in whirl-pak bag |  |
| Food | fo_cov | fo_cov | n | F | Food covered/not covered before serving | 1=Covered  2=Not covered  3=Not observed |
| Food | fo_fec | fo_fec | n | F | Sample was taken within 3m of feces | 1=Yes  0=No |
| Food | fo_lat | fo_lat | n | F | Sample was taken within 30m of latrine or defecation area | 1=Yes  0=No |
| Food | fo_sw | fo_sw | n | F | Sample was taken within 3m of a sewage outfall or an open drain | 1=Yes  0=No |
| Food | fo_wgt | fo_wgt | n | F | Sample weight (g) if sample is prepared food |  |
| Food | fo_vol | fo_vol | n | F | Sample volume (mL) if sample is prepared food |  |
| Handrinse | hr_who | hr_who | n | R | Participant | 1=Vendor  2=Caregiver  3=Teacher  4=Other adult  5=Child 5-12 years  6=Child under 5 |
| Handrinse | hr_sex | hr_sex | n | R | Sex of participant | 1=Male  2=Female |
| Handrinse | hr_un | hr_un | n | F | Dirt is visible under the nails | 1=Yes  0=No |
| Handrinse | hr_fp | hr_fp | n | F | Dirt is visible on the finger pads | 1=Yes  0=No |
| Handrinse | hr_hdrt | hr_hdrt | n | F | Dirt is visible on the palms | 1=Yes  0=No |
| Helminth | hel_sdate | hel_sdat | s | F | Assay start date |  |
| Helminth | hel_cdate | hel_cdat | s | F | Assay end date |  |
| Helminth | hel_vdate | hel_vdat | s | F | Viability assay date |  |
| Helminth | hm_denom | hm_denom | n | C | Denominator, equivalent volume |  |
| Helminth | e_ascaris | e_ascari | n | F | Number of Ascaris eggs/mL |  |
| Helminth | l_ascaris | l_ascari | n | F | Number of Ascaris larvae/mL |  |
| Helminth | v_ascaris | v_ascari | n | F | Number of viable Ascaris/mL |  |
| Helmith | asc_pos | asc_pos | n | C | Sample positive for Ascaris (either eggs or larvae) | 1=Positive  2=Negative |
| Helminth | e_strong | e_strong | n | F | Number of Strongyloides eggs/mL |  |
| Helminth | l_strong | l_strong | n | F | Number of Strongyloides larvae/mL |  |
| Helminth | v_strong | v_strong | n | F | Number of viable Strongyloides/mL |  |
| Helmith | str_pos | str_pos | n | C | Sample positive for strongyloides (either eggs or larvae) | 1=Positive  2=Negative |
| Helminth | e_taenia | e_taenia | n | F | Number of Taenia eggs/mL |  |
| Helminth | l_taenia | l_taenia | n | F | Number of Taenia larvae/mL |  |
| Helminth | v_taenia | v_taenia | n | F | Number of viable Taenia/mL |  |
| Helmith | tea_pos | tea_pos | n | C | Sample positive for Taenia (either eggs or larvae) | 1=Positive  2=Negative |
| Helminth | e_schisto | e_schist | n | F | Number of Schistosoma eggs/mL |  |
| Helminth | l_schisto | l_schist | n | F | Number of Schistosoma larvae/mL |  |
| Helminth | v_schisto | v_schist | n | F | Number of viable Schistosoma/mL |  |
| Helmith | sch_pos | sch_pos | n | C | Sample positive for Schistosoma (either eggs or larvae) | 1=Positive  2=Negative |
| Helminth | e_hook | e_hook | n | F | Number of Hookworm eggs/mL |  |
| Helminth | l_hook | l_hook | n | F | Number of Hookworm larvae/mL |  |
| Helminth | v_hook | v_hook | n | F | Number of viable Hookworms/mL |  |
| Helmith | hook_pos | hook_pos | n | C | Sample positive for Hookworm (either eggs or larvae) | 1=Positive  2=Negative |
| Helminth | e_trich | e_trich | n | F | Number of Trichuris eggs/mL |  |
| Helminth | l_trich | l_trich | n | F | Number of Trichuris larvae/mL |  |
| Helminth | v_trich | v_trich | n | F | Number of viable Trichuris/mL |  |
| Helmith | tch_pos | tch_pos | n | C | Sample positive for Trichuris (either eggs or larvae) | 1=Positive  2=Negative |
| Helminth | other1name | other1na | s | F | Name of other 1 helminth |  |
| Helminth | e_other1 | e_other1 | n | F | Number of other 1 eggs/mL |  |
| Helminth | l_other1 | l_other1 | n | F | Number of other 1 larvae/mL |  |
| Helminth | v_other1 | v_other1 | n | F | Number of viable other 1/mL |  |
| Helminth | other2name | other2na | s | F | Name of other 2 helminth |  |
| Helminth | e_other2 | e_other2 | n | F | Number of other 2 eggs/mL |  |
| Helminth | l_other2 | l_other2 | n | F | Number of other 2 larvae/mL |  |
| Helminth | v_other2 | v_other2 | n | F | Number of viable other 2/mL |  |
| HH Conditions | hh_long | hh_long | n | C | Longitude (DD) |  |
| HH Conditions | hh_lat | hh_lat | n | C | Latitude (DD) |  |
| HH Conditions | hh_st | hh_st | s | F | Start time |  |
| HH Conditions | hh_et | hh_et | s | F | End time |  |
| HH Conditions | hh_date | hh_date | s | F | Date |  |
| HH Conditions | hh_notes | hh_notes | s | F | Notes |  |
| HH Conditions | hh_101 | hh_101 | n | F | Respondent status | 1=Female head of HH  2=Male head of HH  3=Female in HH  4=Other |
| HH Conditions | hh_102 | hh_102 | n | F | Tenancy status | 1=Rent  2=Own |
| HH Conditions | hh_103 | hh_103 | n | F | Level of education | 1=No formal education  2=Some primary  3=Completed primary  4=Completed secondary  5=More than secondary |
| HH Conditions | hh_104 | hh_104 | n | F | Compound or HH | 1=Compound  2=HH |
| HH Conditions | hh_105 | hh_105 | n | F | There is a business in the HH | 1=Yes  2=No  3=DK  4=No response |
| HH Conditions | hh_106 | hh_106 | n | F | Type of business | 1=Prepared food vending  2=Hairdressing  3=Other |
| HH Conditions | hh_107 | hh_107 | n | F | Religion | 1=Christian  2=Moslem  3=Traditional  4=None  5=Other |
| HH Conditions | hh_108 | hh_108 | n | F | Number of HHs in compound |  |
| HH Conditions | hh_109 | hh_109 | n | F | Number of people in the compound |  |
| HH Conditions | hh_110 | hh_110 | n | F | Number of people in the HH |  |
| HH Conditions | hh_111 | hh_111 | n | F | Number of male adults (18 and older) in the HH |  |
| HH Conditions | hh_112 | hh_112 | n | F | Number of female adults (18 and older) in the HH |  |
| HH Conditions | hh_113 | hh_113 | n | F | Number of children under 5 in the HH |  |
| HH Conditions | hh_114 | hh_114 | n | F | Number of children 5-12 years in the HH |  |
| HH Conditions | hh_115 | hh_115 | n | F | Number of people 13-17 years in the HH |  |
| HH Conditions | hh_116 | hh_116 | n | F | HH has electricity | 1=Yes  2=No  3=No response |
| HH Conditions | hh_117 | hh_117 | n | F | HH has a radio | 1=Yes  2=No  3=No response |
| HH Conditions | hh_118 | hh_118 | n | F | HH has a TV | 1=Yes  2=No  3=No response |
| HH Conditions | hh_119 | hh_119 | n | F | HH has a refrigerator | 1=Yes  2=No  3=No response |
| HH Conditions | hh_120 | hh_120 | n | F | HH has a bike | 1=Yes  2=No  3=No response |
| HH Conditions | hh_121 | hh_121 | n | F | HH has a motorcycle | 1=Yes  2=No  3=No response |
| HH Conditions | hh_122 | hh_122 | n | F | HH has a car | 1=Yes  2=No  3=No response |
| HH Conditions | hh_123 | hh_123 | n | F | HH employs a domestic worker not related to the head of the HH | 1=Yes  2=No  3=No response |
| HH Conditions | hh_201 | hh_201 | n | F | Primary source of drinking water | 1=Sachet  2=Tap from pipe  3=Tap from tank  4=Well  5=Water truck  6= Rainwater  7=Other  8=DK  9=No response |
| HH Conditions | hh_202 | hh_202 | n | F | Months a year with access to this water source |  |
| HH Conditions | hh_203 | hh_203 | n | F | Frequency the HH replenishes the water | 1=Everyday  2=A few times a week  3=Once a week or less |
| HH Conditions | hh_204 | hh_204 | n | F | Volume the child drinks everyday |  |
| HH Conditions | hh_204a | hh_204a | n | F | Unit for the volume the child drinks | 1=Cups  2=Sachets |
| HH Conditions | hh_205 | hh_205 | s | F | Water source for cooking and hygiene | 1=Sachet/water bottle  2=Tap from pipe network  3=Tap from polytank  4=Well  5=Water truck  6= Rainwater  7=Other  8=DK  9=No response |
| HH Conditions | hh_205a | hh_205a | n | C | Sachet, from hh_205 | 1=Yes  0=No |
| HH Conditions | hh_205b | hh_205b | n | C | Tap from pipe network, from hh_205 | 1=Yes  0=No |
| HH Conditions | hh_205c | hh_205c | n | C | Tap from polytank, from hh_205 | 1=Yes  0=No |
| HH Conditions | hh_205d | hh_205d | n | C | Well, from hh_205 | 1=Yes  0=No |
| HH Conditions | hh_205e | hh_205e | n | C | Water truck, from hh_205 | 1=Yes  0=No |
| HH Conditions | hh_205f | hh_205f | n | C | Rainwater, from hh_205 | 1=Yes  0=No |
| HH Conditions | hh_205g | hh_205g | n | C | Other, from hh_205 | 1=Yes  0=No |
| HH Conditions | hh_301 | hh_301 | n | F | Number of latrines |  |
| HH Conditions | hh_302 | hh_302 | n | F | Number of HHs sharing the latrine |  |
| HH Conditions | hh_303 | hh_303 | n | F | Where females in the HH ≥12 years typically defecate when at home | 1=Compound latrine  2=Public latrine  3=Bag/flying toilet  4=Chamber pot  5=Outside  6=Aqua privy  7=Other  8=DK  9=No response |
| HH Conditions | hh_304 | hh_304 | n | F | Same defecation site for other females in the compound ≥12 years | 1=Yes  2=No 3=DK  4=No response |
| HH Conditions | hh_305 | hh_305 | n | F | Where males in the HH ≥12 years typically defecate | 1=Compound latrine  2=Public latrine  3=Bag/flying toilet  4=Chamber pot  5=Outside  6=Aqua privy  7=Other  8=DK  9=No response |
| HH Conditions | hh_306 | hh_306 | n | F | Same defecation site for males in the compound ≥12 years | 1=Yes  2=No  3=DK  4=No response |
| HH Conditions | hh_307 | hh_307 | n | F | Where children in the HH 5-12 years old typically defecate | 1=Compound latrine  2=Public latrine  3=Bag/flying toilet  4=Chamber pot  5=Outside  6=Aqua privy  7=Other  8=DK  9=No response |
| HH Conditions | hh_308 | hh_308 | n | F | Same defecation site for children in the compound 5-12 years old | 1=Yes  2=No  3=DK  4=No response |
| HH Conditions | hh_309 | hh_309 | n | F | Where the youngest child last defecated | 1=Compound latrine  2=Public latrine  3=Potty  4=Diaper/nappy  5=Ground outside the compound  6=Ground inside the compound  7=Drain/gutter  8=Aqua privy  9=DK  10=No response |
| HH Conditions | hh_310 | hh_310 | n | F | Age of the youngest child |  |
| HH Conditions | hh_310a | hh_310a | n | F | Unit for age of the youngest child | 1=Years  2=Months |
| HH Conditions | hh_311 | hh_311 | n | F | How feces were disposed for the youngest child | 1=Rubbish  2=Drain/gutter  3=Public latrine  4=Compound latrine  5=Left on ground  6=DK  7=No response |
| HH Conditions | hh_312 | hh_312 | n | F | Mothers in the compound use potties | 1=Yes  2=No  3=DK  4=No response |
| HH Conditions | hh_313 | hh_313 | n | F | Where mothers in the compound dispose of child's feces | 1=Rubbish  2=Drain/gutter  3=Public latrine  4=Compound latrine  5=Left on ground  6=DK  7=No response |
| HH Conditions | hh_314 | hh_314 | n | F | Other mothers leave child feces on the compound ground | 1=Yes  2=No  3=DK  4=No response |
| HH Conditions | hh_315 | hh_315 | n | F | Amount of money spent on public latrines yesterday (not including showers) by HH members |  |
| HH Conditions | hh_315a | hh_315a | n | F | Unit for amount of money spent on public latrines yesterday by HH members | 1=Pesewas  2=Cedis |
| HH Conditions | hh_316 | hh_316 | n | F | Where respondent last bathed | 1=In compound  2=Public bath  3=Other  4=No response |
| HH Conditions | hh_317 | hh_317 | n | F | Amount of money spent on bathing facilities yesterday by HH members |  |
| HH Conditions | hh_317a | hh_317a | n | F | Unit for amount of money spent on bathing facilities yesterday by HH members | 1=Pesewas  2=Cedis |
| HH Conditions | hh_318 | hh_318 | n | F | Amount spent on drinking water for HH |  |
| HH Conditions | hh_318a | hh_318a | n | F | Unit for amount spent on drinking water for HH | 1=Pesewas 2=Cedis |
| HH Conditions | hh_319 | hh_319 | n | F | Amount spent on water for all purposes yesterday for HH |  |
| HH Conditions | hh_319a | hh_319a | n | F | Unit for amount spent on water for all purposes yesterday for HH | 1=Pesewas  2=Cedis |
| HH Conditions | hh_320 | hh_320 | n | F | Primary rubbish disposal method for HH | 1=Disposal pit in compound  2=Private collection service  3=Private local collection  4=Open site/drain/sea  5=Dumped  6=Burned  7=Other  8=DK  9=No response |
| HH Conditions | hh_321 | hh_321 | n | F | Amount spent on rubbish disposal yesterday |  |
| HH Conditions | hh_321a | hh_321a | n | F | Unit for amount spent on rubbish disposal yesterday | 1=Pesewas  2=Cedis |
| HH Conditions | hh_401 | hh_401 | n | F | Observed child had diarrhea in past two weeks | 1=Yes  2=No  3=DK  4=No response |
| HH Conditions | hh_402 | hh_402 | n | F | Diarrhea was bloody | 1=Yes  2=No  3=DK  4=No response |
| HH Conditions | hh_403 | hh_403 | n | F | Respondent has ever dewormed child | 1=Yes  2=No  3=DK  4=No response |
| HH Conditions | hh_404 | hh_404 | n | F | Last time respondent dewormed child |  |
| HH Conditions | hh_404a | hh_404a | n | F | Unit for last time respondent dewormed child | 1=Days  2=Months  3=Years |
| HH Conditions | hh_501 | hh_501 | n | F | Type of toilet facility on compound | 1=No facility/bush/field  2=Traditional pit latrine  3=KVIP (double)  4=VIP (single)  5=Bucket/pan  6=Pour flush  7=Flush toilet  8=Not allowed to see |
| HH Conditions | hh_502 | hh_502 | n | F | Slab material | 1=Wood/natural  2=Cement  3=Dirt/unfinished  4=Porcelain/tile |
| HH Conditions | hh_503 | hh_503 | n | F | Feces present | 1=None  2=Some  3=A lot |
| HH Conditions | hh_504 | hh_504 | n | F | Odor present | 1=None  2=Some  3=Terrible |
| HH Conditions | hh_505 | hh_505 | n | F | Flies present | 1=None  2=Some  3=Swarms |
| HH Conditions | hh_506 | hh_506 | n | F | Safety of latrine | 1=Secure stance  2=Slightly insecure  3=Threat of falling through |
| HH Conditions | hh_507 | hh_507 | n | F | Privacy of latrine | 1=Door closes/no holes  2=Door partly closes/peepholes  3=No door |
| HH Conditions | hh_508 | hh_508 | n | F | Anal cleansing materials present | 1=Water  2=Newspaper  3=Toilet tissue  4=Other  5=None |
| HH Conditions | hh_509 | hh_509 | n | F | HW station present | 1=Yes  2=No |
| HH Conditions | hh_510 | hh_510 | n | F | Water present at hw station | 1=Yes  2=No |
| HH Conditions | hh_511 | hh_511 | n | F | Soap present at hw station | 1=Yes  2=No |
| HH Conditions | hh_512 | hh_512 | n | F | Ash present at hw station | 1=Yes  2=No |
| HH Conditions | hh_513 | hh_513 | n | F | HW station is near latrine | 1=Yes  2=No |
| HH Conditions | hh_514 | hh_514 | n | F | Type of drinking water container | 1=Narrow mouth (≤6cm)  2=Wide mouth (>6cm)  3=Couldn't see |
| HH Conditions | hh_515 | hh_515 | n | F | Drinking water container covered | 1=Covered  2=Uncovered |
| HH Conditions | hh_516 | hh_516 | n | F | How water is prepared for child to drink | 1=Dip  2=Pour  3=Tap  4=Sachet  5=No water available |
| HH Conditions | hh_517 | hh_517 | n | F | Feces present in compound | 1=Yes  2=No |
| HH Conditions | hh_518 | hh_518 | n | F | Animals present in compound | 1=Yes  2=No |
| HH Conditions | hh_519 | hh_519 | s | F | Type of animal present | 1=Goats  2=Chickens  3=Pigs  4=Dogs  5=Cats  6=Other |
| HH Conditions | hh_519a | hh_519a | n | C | Goats, from hh_519 | 1=Yes  0=No |
| HH Conditions | hh_519b | hh_519b | n | C | Chickens, from hh_519 | 1=Yes  0=No |
| HH Conditions | hh_519c | hh_519c | n | C | Pigs, from hh_519 | 1=Yes  0=No |
| HH Conditions | hh_519d | hh_519d | n | C | Dogs, from hh_519 | 1=Yes  0=No |
| HH Conditions | hh_519e | hh_519e | n | C | Cats, from hh_519 | 1=Yes  0=No |
| HH Conditions | hh_519f | hh_519f | n | C | Other, from hh_519 | 1=Yes  0=No |
| HH Conditions | hh_520 | hh_520 | n | F | Food waste present on ground | 1=Yes  2=No |
| HH Survey | hhs_long | hhs_long | n | C | Longitude (DD) |  |
| HH Survey | hhs_lat | hhs_lat | n | C | Latitude (DD) |  |
| HH Survey | hhs_date | hhs_date | s | F | Date |  |
| HH Survey | hhs_notes | hhs_notes | s | F | Notes |  |
| HH Survey | hhs_101 | hhs_101 | n | F | Respondent status | 1=Female head of HH  2=Male head of HH  3=Female in HH  4=Male in HH |
| HH Survey | hhs_102 | hhs_102 | n | F | Tenancy status | 1=Renter  2=Owner |
| HH Survey | hhs_103 | hhs_103 | n | F | Length on time lived in HH |  |
| HH Survey | hhs_103a | hhs_103a | n | F | Unit for length lived in HH | 1=Months  2=Years |
| HH Survey | hhs_104 | hhs_104 | n | F | Level of education | 1=No formal education  2=Some primary  3=Completed primary  4=Some secondary  5=Completed secondary  6=Higher than secondary  7=No response |
| HH Survey | hhs_105 | hhs_105 | n | F | Religion | 1=Christian  2=Moslem  3=Traditional  4=No religion  5=Other  6=No response |
| HH Survey | hhs_106 | hhs_106 | n | F | Mother tongue | 1=Ga  2=Twi  3=Fante  4=Hausa  5=Other  6=No response |
| HH Survey | hhs_107 | hhs_107 | n | F | There is a business in the HH | 1=Yes  2=No  3=No response |
| HH Survey | hhs_108 | hhs_108 | n | F | Type of business | 1=Food vending  2=Other |
| HH Survey | hhs_109 | hhs_109 | n | F | Number of HHs in compound |  |
| HH Survey | hhs_110 | hhs_110 | n | F | Number of people in the compound |  |
| HH Survey | hhs_111 | hhs_111 | n | F | Number of people in the HH |  |
| HH Survey | hhs_112 | hhs_112 | n | F | Number of male adults (18 and older) in the HH |  |
| HH Survey | hhs_113 | hhs_113 | n | F | Number of female adults (18 and older) in the HH |  |
| HH Survey | hhs_114 | hhs_114 | n | F | Number of children under 5 in the HH |  |
| HH Survey | hhs_115 | hhs_115 | n | F | Number of children 5-12 years in the HH |  |
| HH Survey | hhs_116 | hhs_116 | n | F | Number of people 13-17 years in the HH |  |
| HH Survey | hhs_117 | hhs_117 | n | F | HH has electricity | 1=Yes  2=No  3=No response |
| HH Survey | hhs_118 | hhs_118 | n | F | HH has a radio | 1=Yes  2=No  3=No response |
| HH Survey | hhs_119 | hhs_119 | n | F | HH has a TV | 1=Yes  2=No  3=No response |
| HH Survey | hhs_120 | hhs_120 | n | F | HH has a refrigerator | 1=Yes  2=No  3=No response |
| HH Survey | hhs_121 | hhs_121 | n | F | HH has a bike | 1=Yes  2=No  3=No response |
| HH Survey | hhs_122 | hhs_122 | n | F | HH has a motorcycle | 1=Yes  2=No  3=No response |
| HH Survey | hhs_123 | hhs_123 | n | F | HH has a car | 1=Yes  2=No  3=No response |
| HH Survey | hhs_124 | hhs_124 | n | F | HH employs a domestic worker not related to the head of the HH | 1=Yes  2=No  3=No response |
| HH Survey | hh_201 | hh_201 | n | F | Primary source of drinking water | 1=Sachet  2=Tap from pipe  3=Tap from tank  4=Well  5=Water truck  6= Rainwater  7=Other  8=DK  9=No response |
| HH Survey | hh_201a | hh_201a | s | F | Other source of drinking water |  |
| HH Survey | hhs_202 | hhs_202 | n | F | Frequency the HH replenishes the water | 1=Everyday  2=A few times a week  3=Once a week or less |
| HH Survey | hhs_203 | hhs_203 | n | F | Volume the child drinks everyday |  |
| HH Survey | hhs_203a | hhs_203a | n | F | Unit for the volume the child drinks | 1=Cups  2=Sachets |
| HH Survey | hhs_204 | hhs_204 | n | F | Water source for cooking and hygiene | 1=Sachet  2=Tap from pipe  3=Tap from tank  4=Well  5=Water truck  6= Rainwater  7=Other  8=DK  9=No response |
| HH Survey | hhs_204a | hhs_204a | s | F | Other water source for cooking and hygiene |  |
| HH Survey | hhs_301 | hhs_301 | n | F | Number of latrines |  |
| HH Survey | hhs_302 | hhs_302 | n | F | Number of HHs sharing the latrine |  |
| HH Survey | hhs_303 | hhs_303 | n | F | Where children in the HH 5-12 years old typically defecate | 1=Compound latrine  2=Public latrine  3=Bag/flying toilet  4=Chamber pot  5=On ground outside compound  6=On ground inside compound  7=Beach  8=Other  9=DK  10=No response |
| HH Survey | hhs_303a | hhs_303a | s | F | Other place children in the HH 5-12 years old defecate |  |
| HH Survey | hhs_304 | hhs_304 | n | F | Same defecation site for children in the compound 5-12 years old | 1=Yes  2=No  3=DK  4=No response |
| HH Survey | hhs_305 | hhs_305 | n | F | Where the youngest child last defecated | 1=Compound latrine  2=Public latrine  3=Potty  4=Diaper/nappy  5=On ground outside compound  6=On ground inside compound  7=Drain/gutter  8=DK  9=No response  10=DK |
| HH Survey | hhs_306 | hhs_306 | n | F | Age of the youngest child |  |
| HH Survey | hhs_306a | hhs_306a | n | F | Unit for age of the youngest child | 1=Years  2=Months |
| HH Survey | hhs_307 | hhs_307 | n | F | How feces were disposed for the youngest child | 1=Rubbish  2=Drain/gutter  3=Public latrine  4=Compound latrine  5=Washed diaper/nappy  6=Left on ground  6=DK  7=No response |
| HH Survey | hhs_308 | hhs_308 | n | F | Mothers in the compound use potties | 1=Yes  2=No  3=DK  4=No response |
| HH Survey | hhs_309 | hhs_309 | n | F | Other mothers leave child feces on the compound ground | 1=Yes  2=No  3=DK  4=No response |
| HH Survey | hhs_310 | hhs_310 | n | F | Frequency children are seen defecating in the drain near HH | 1=Everyday  2=Sometimes  3=Never  4=No drain near HH  5=No response |
| HH Survey | hhs_311 | hhs_311 | n | F | Frequency adults are seen defecating in the drain near HH | 1=Everyday  2=Sometimes  3=Never  4=No drain near HH  5=No response |
| HH Survey | hhs_312 | hhs_312 | n | F | Amount of money spent on public latrines yesterday (not including showers) by HH members |  |
| HH Survey | hhs_312a | hhs_312a | n | F | Unit for amount of money spent on public latrines yesterday by HH members | 1=Pesewas  2=Cedis |
| HH Survey | hhs_313 | hhs_313 | n | F | Where respondent last bathed | 1=In compound  2=Public bath  3=Other  4=No response |
| HH Survey | hhs_313a | hhs_313a | s | F | Other place respondent last bathed |  |
| HH Survey | hh_314 | hh_314 | n | F | Amount of money spent on bathing facilities yesterday by HH members |  |
| HH Survey | hh_314a | hh_314a | n | F | Unit for amount of money spent on bathing facilities yesterday by HH members | 1=Pesewas  2=Cedis |
| HH Survey | hhs_315 | hhs_315 | n | F | Amount spent on drinking water for HH |  |
| HH Survey | hhs_315a | hhs_315a | n | F | Unit for amount spent on drinking water for HH | 1=Pesewas  2=Cedis |
| HH Survey | hhs_316 | hhs_316 | n | F | Amount spent on water for all purposes yesterday for HH |  |
| HH Survey | hhs_316a | hhs_316a | n | F | Unit for amount spent on water for all purposes yesterday for HH | 1=Pesewas  2=Cedis |
| HH Survey | hhs_317 | hhs_317 | n | F | Primary rubbish disposal method for HH | 1=Disposal pit in compound  2=Private collection service  3=Private local collection  4=Open site/drain/sea  5=Dumped  6=Burned  7=Other  8=DK  9=No response |
| HH Survey | hhs_317a | hhs_317a | s | F | Other rubbish disposal method for HH |  |
| HH Survey | hhs_318 | hhs_318 | n | F | Amount spent on rubbish disposal yesterday |  |
| HH Survey | hhs_318a | hhs_318a | n | F | Unit for amount spent on rubbish disposal yesterday | 1=Pesewas  2=Cedis |
| HH Survey | hhs_401 | hhs_401 | n | F | Observed child had diarrhea in past two weeks | 1=Yes  2=No  3=DK  4=No response |
| HH Survey | hhs_402 | hhs_402 | n | F | Diarrhea was bloody | 1=Yes  2=No  3=DK  4=No response |
| HH Survey | hhs_403 | hhs_403 | n | F | Respondent has ever dewormed child | 1=Yes  2=No  3=DK  4=No response |
| HH Survey | hhs_404 | hhs_404 | n | F | Last time respondent dewormed child |  |
| HH Survey | hhs_404a | hhs_404a | n | F | Unit for last time respondent dewormed child | 1=Days  2=Months  3=Years |
| HH Survey | hhs_501 | hhs_501 | n | F | Type of toilet facility on compound | 1=No facility/bush/field  2=Traditional pit latrine  3=KVIP (double)  4=VIP (single)  5=Bucket/pan  6=Pour flush  7=Flush toilet  8=Other  9=No response |
| HH Survey | hhs_502 | hhs_502 | n | F | Sink for handwashing after defecation | 1=Yes  2=No  3=No response |
| HH Survey | hhs_503 | hhs_503 | n | F | Own container for storing drinking water | 1=Yes  2=No  3=No response |
| HH Survey | hhs_504 | hhs_504 | n | F | Type of drinking water container | 1=Narrow mouth (≤6cm)  2=Wide mouth (>6cm)  3=Couldn't see |
| HH Survey | hhs_505 | hhs_505 | n | F | Drinking water container covered | 1=Covered  2=Uncovered |
| HH Survey | hhs_506 | hhs_506 | n | F | Feces present in compound | 1=Yes  2=No |
| HH Survey | hhs_507 | hhs_507 | n | F | Animals present in compound | 1=Yes  2=No |
| HH Survey | hhs_508 | hhs_508 | s | F | Type of animal present | 1=Goats  2=Chickens  3=Pigs  4=Dogs  5=Cats  6=Other |
| HH Survey | hhs_509 | hhs_509 | n | F | Food waste present on ground | 1=Yes  2=No |
| HH Survey | hhs_601 | hhs_601 | n | F | Frequency respondent used public latrine in last week | 1=Everyday  2=A few times a week  3=Once a week  4=Never  5=No response |
| HH Survey | hhs_602 | hhs_602 | n | F | Frequency respondent went to the market in last week | 1=Everyday  2=A few times a week  3=Once a week  4=Never  5=No response |
| HH Survey | hhs_603 | hhs_603 | n | F | Frequency respondent ate raw produce in last week | 1=Everyday  2=A few times a week  3=Once a week  4=Never  5=No response |
| HH Survey | hhs_604 | hhs_604 | n | F | Frequency respondent bought vendor food in last week | 1=Everyday  2=A few times a week  3=Once a week  4=Never  5=No response |
| HH Survey | hhs_701 | hhs_701 | n | F | Frequency respondent went to the beach in the last month | 1=Everyday  2=5-10 times  3=1-4 times  4=Never  5=No response |
| HH Survey | hhs_702 | hhs_702 | n | F | Frequency youngest child goes to beach in a regular week | 1=Everyday  2=Once a week  3=Twice a week  4=None  5=Other |
| HH Survey | hhs_703 | hhs_703 | n | F | Respondent's job puts them in contact with seawater | 1=Yes  2=No  3=DK  4=No response |
| HH Survey | hhs_704 | hhs_704 | n | F | Someone else in HH has job that puts them in contact with seawater | 1=Yes  2=No  3=DK  4=No response |
| HH Survey | hhs_705 | hhs_705 | n | F | Frequency respondent enters seawater when at beach | 1=Every time  2=Sometimes  3=Never  4=No response |
| HH Survey | hhs_801 | hhs_801 | n | F | Compound flooded in last year | 1=Yes  2=No  3=DK  4=No response |
| HH Survey | hhs_901 | hhs_901 | n | F | Children in HH attend nursery school | 1=Yes  2=No  3=DK  4=No response |
| HH Survey | hhs_902 | hhs_902 | n | F | Number of children who attend nursery school |  |
| HH Survey | hhs_903 | hhs_903 | n | F | Number of days/week youngest child attends nursery |  |
| HH Survey | hhs_904 | hhs_904 | n | F | Number of hours/day youngest child attends nursery |  |
| HH Survey | hhs_905 | hhs_905 | n | F | Frequency child buys food at school | 1=Every time  2=Sometimes  3=Never  4=No response |
| HH Survey | hhs_906 | hhs_906 | n | F | Reason child doesn't buy food at school | 1=Food carried from home  2=School feeding program |
| Large Volume Water | lw_type | lw_type | s | R | Type of water collected | 1=Drinking water  2=Ocean water |
| Large Volume Water | lo_where | lo_where | s | F | If ocean water, where it was collected |  |
| Large Volume Water | lo_ot | lo_ot | s | R | Other source if ocean water | 1=Open source  2=Inland  3=Other |
| Large Volume Water | ld_source | ld_sourc | s | R | If drinking water, where it was collected | 1=Public tap  2=Compound/private tap  3=Hand-dug well  4=Tube well/borehole  5=Tanker truck  6=Other |
| Large Volume Water | ld_hsot | ld_hsot | s | F | Other source, if drinking water |  |
| Large Volume Water | ld_pay | ld_pay | n | F | Pay-per-use | 1=Yes  0=No |
| Large Volume Water | ld_prc | ld_prc | n | F | Pay-per-use price (pesewas) |  |
| Large Volume Water | ld_vol | ld_vol | n | F | Pay-per-use volume (L) |  |
| Large Volume Water | lw_trs | lw_trs | n | F | Sample was taken within 3m of trash | 1=Yes  0=No |
| Large Volume Water | lw_fec | lw_fec | n | F | Sample was taken within 3m of feces | 1=Yes  0=No |
| Large Volume Water | lw_lat | lw_lat | n | F | Sample was taken within 30m of a latrine or defecation area | 1=Yes  0=No |
| Large Volume Water | ld_turb | ld_turb | n | F | Turbidity |  |
| Large Volume Water | ld_clf | ld_clf | n | F | Free chlorine residual |  |
| Large Volume Water | ld_clt | ld_clt | n | F | Total chlorine residual |  |
| Large Volume Water | lw_sal | lw_sal | n | F | Salinity |  |
| Large Volume Water | ld_temp | ld_temp | n | F | Temperature |  |
| Large Volume Water | ld_tvol | ld_tvol | n | F | Starting volume (L) |  |
| Large Volume Water | ld_ufvol | ld_ufvol | n | F | Post UF volume |  |
| Large Volume Water | ld_lnotes | ld_lnote | s | F | Lab notes |  |
| Latrine Conditions | la_long | la_long | n | C | Longitude (DD) |  |
| Latrine Conditions | la_lat | la_lat | n | C | Latitude (DD) |  |
| Latrine Conditions | la_st | la_st | s | F | Start time for observation |  |
| Latrine Conditions | la_et | la_et | s | F | End time for observation |  |
| Latrine Conditions | la_date | la_date | s | F | Date |  |
| Latrine Conditions | la_a | la_a | n | F | Latrine setting | 1=Nursery  2=Basic school  3=Public latrine in a market  4=Public latrine at the beach  5=Public latrine in the community |
| Latrine Conditions | la_b | la_b | n | F | Handwashing stations present | 1=Yes  2=No |
| Latrine Conditions | la_b1 | la_b1 | n | F | Number of HW stations |  |
| Latrine Conditions | la_b2 | la_b2 | n | F | Number of HW stations with soap |  |
| Latrine Conditions | la_b3 | la_b3 | n | F | Number of HW stations with water |  |
| Latrine Conditions | la_c | la_c | s | F | Cleansing materials provided | 1=Water  2=Newspaper  3=Toilet tissue  4=Other  5=None |
| Latrine Conditions | la_ca | la_ca | n | C | Water, from la_c | 1=Yes  0=No |
| Latrine Conditions | la_cc | la_cc | n | C | Newspaper, from la_c | 1=Yes  0=No |
| Latrine Conditions | la_cd | la_cd | n | C | Toilet tissue, from la_c | 1=Yes  0=No |
| Latrine Conditions | la_ce | la_ce | n | C | Other, from la_c | 1=Yes  0=No |
| Latrine Conditions | la_cf | la_cf | n | C | None, from la_c | 1=Yes  0=No |
| Latrine Conditions | la_d | la_d | n | F | Management type | 1=Municipal  2=Private  3=Community |
| Latrine Conditions | la_e | la_e | n | F | Number of years the latrine has been in operation |  |
| Latrine Conditions | la_f | la_f | n | F | Cost to use the latrine |  |
| Latrine Conditions | la_f1 | la_f1 | n | F | Units for cost to use the latrine | 1=Pesewas  2=Cedis |
| Latrine Conditions | la_g1 | la_g1 | n | F | Cleansing materials included | 1=Included  2=Extra |
| Latrine Conditions | la_g | la_g | n | F | Cost for cleansing materials |  |
| Latrine Conditions | la_g2 | la_g2 | n | F | Units for cost for cleansing materials | 1=Pesewas  2=Cedis |
| Latrine Conditions | la_h1 | la_h1 | n | F | Opening time |  |
| Latrine Conditions | la_h2 | la_h2 | n | F | Closing time |  |
| Latrine Conditions | la_h3 | la_h3 | n | F | Total hours open |  |
| Latrine Conditions | la_i | la_i | s | F | Days the latrine is open | 1=Sunday  2=Monday  3=Tuesday  4=Wednesday  5=Thursday  6=Friday  7=Saturday  8=All days |
| Latrine Conditions | la_ia | la_ia | n | C | Sunday, from la_i | 1=Yes  0=No |
| Latrine Conditions | la_ib | la_ib | n | C | Monday, from la_i | 1=Yes  0=No |
| Latrine Conditions | la_ic | la_ic | n | C | Tuesday, from la_i | 1=Yes  0=No |
| Latrine Conditions | la_id | la_id | n | C | Wednesday, from la_i | 1=Yes  0=No |
| Latrine Conditions | la_ie | la_ie | n | C | Thursday, from la_i | 1=Yes  0=No |
| Latrine Conditions | la_if | la_if | n | C | Friday, from la_i | 1=Yes  0=No |
| Latrine Conditions | la_ig | la_ig | n | C | Saturday, from la_i | 1=Yes  0=No |
| Latrine Conditions | la_j | la_j | n | F | Shower associated with the latrine | 1=Yes  2=No |
| Latrine Conditions | la_ja | la_ja | n | F | Frequency that feces are found in the shower | 1=Never  2=Less than once a week  3=At least once a week  4=Everyday |
| Latrine Conditions | la_jb | la_jb | n | F | Cost of shower |  |
| Latrine Conditions | la_jb1 | la_jb1 | n | F | Units for cost of shower | 1=Pesewas  2=Cedis |
| Latrine Conditions | la_mus | la_mus | n | F | Male- number of urinal stalls |  |
| Latrine Conditions | la_mls | la_mls | n | F | Male- number of latrines stalls |  |
| Latrine Conditions | la_ml1 | la_ml1 | n | F | Male- number of traditional pit latrines |  |
| Latrine Conditions | la_ml2 | la_ml2 | n | F | Male- number of KVIP (double) |  |
| Latrine Conditions | la_ml3 | la_ml3 | n | F | Male- number of VIP (single) |  |
| Latrine Conditions | la_ml4 | la_ml4 | n | F | Male- number of bucket/pan latrines |  |
| Latrine Conditions | la_ml5 | la_ml5 | n | F | Male- number of pour flush latrines |  |
| Latrine Conditions | la_ml6 | la_ml6 | n | F | Male- number of flush toilets |  |
| Latrine Conditions | la_ms1 | la_ms1 | n | F | Male- number with wood/natural slabs |  |
| Latrine Conditions | la_ms2 | la_ms2 | n | F | Male- number with cement slabs |  |
| Latrine Conditions | la_ms3 | la_ms3 | n | F | Male- number with dirt/unfinished slabs |  |
| Latrine Conditions | la_ms4 | la_ms4 | n | F | Male- number with porcelain/tile slabs |  |
| Latrine Conditions | la_mf1 | la_mf1 | n | F | Male- number with no feces present on slab or walls |  |
| Latrine Conditions | la_mf2 | la_mf2 | n | F | Male- number with some feces present on slab or walls |  |
| Latrine Conditions | la_mf3 | la_mf3 | n | F | Male- number with a lot of feces present on slab or walls |  |
| Latrine Conditions | la_ma1 | la_ma1 | n | F | Male- number with a secure stance |  |
| Latrine Conditions | la_ma2 | la_ma2 | n | F | Male- number with a slightly insecure stance |  |
| Latrine Conditions | la_ma3 | la_ma3 | n | F | Male- number with a stance so insecure there is a threat of falling through |  |
| Latrine Conditions | la_mp1 | la_mp1 | n | F | Male- number with a door that closes and have no holes |  |
| Latrine Conditions | la_mp2 | la_mp2 | n | F | Male- number with a door that doesn't close or have holes |  |
| Latrine Conditions | la_mp3 | la_mp3 | n | F | Male- number with no door |  |
| Latrine Conditions | la_mfly | la_mfly | n | F | Male- flies present | 1=None  2=Some  3=Swarms |
| Latrine Conditions | la_mod | la_mod | n | F | Male- odor present | 1=None  2=Some  3=Terrible |
| Latrine Conditions | la_fus | la_fus | n | F | Female- number of urinal stalls |  |
| Latrine Conditions | la_fls | la_fls | n | F | Female- number of latrines stalls |  |
| Latrine Conditions | la_fl1 | la_fl1 | n | F | Female- number of traditional pit latrines |  |
| Latrine Conditions | la_fl2 | la_fl2 | n | F | Female- number of KVIP (double) |  |
| Latrine Conditions | la_fl3 | la_fl3 | n | F | Female- number of VIP (single) |  |
| Latrine Conditions | la_fl4 | la_fl4 | n | F | Female- number of bucket/pan latrines |  |
| Latrine Conditions | la_fl5 | la_fl5 | n | F | Female- number of pour flush latrines |  |
| Latrine Conditions | la_fl6 | la_fl6 | n | F | Female- number of flush toilets |  |
| Latrine Conditions | la_fs1 | la_fs1 | n | F | Female- number with wood/natural slabs |  |
| Latrine Conditions | la_fs2 | la_fs2 | n | F | Female- number with cement slabs |  |
| Latrine Conditions | la_fs3 | la_fs3 | n | F | Female- number with dirt/unfinished slabs |  |
| Latrine Conditions | la_fs4 | la_fs4 | n | F | Female- number with porcelain/tile slabs |  |
| Latrine Conditions | la_ff1 | la_ff1 | n | F | Female- number with no feces present on slab or walls |  |
| Latrine Conditions | la_ff2 | la_ff2 | n | F | Female- number with some feces present on slab or walls |  |
| Latrine Conditions | la_ff3 | la_ff3 | n | F | Female- number with a lot of feces present on slab or walls |  |
| Latrine Conditions | la_fa1 | la_fa1 | n | F | Female- number with a secure stance |  |
| Latrine Conditions | la_fa2 | la_fa2 | n | F | Female- number with a slightly insecure stance |  |
| Latrine Conditions | la_fa3 | la_fa3 | n | F | Female- number with a stance so insecure there is a threat of falling through |  |
| Latrine Conditions | la_fp1 | la_fp1 | n | F | Female- number with a door that closes and have no holes |  |
| Latrine Conditions | la_fp2 | la_fp2 | n | F | Female- number with a door that doesn't close or have holes |  |
| Latrine Conditions | la_fp3 | la_fp3 | n | F | Female- number with no door |  |
| Latrine Conditions | la_ffly | la_ffly | n | F | Female- flies present | 1=None  2=Some  3=Swarms |
| Latrine Conditions | la_fod | la_fod | n | F | Female- odor present | 1=None  2=Some  3=Terrible |
| Latrine Conditions | la_uus | la_uus | n | F | Unisex- number of urinal stalls |  |
| Latrine Conditions | la_uls | la_uls | n | F | Unisex- number of latrines stalls |  |
| Latrine Conditions | la_ul1 | la_ul1 | n | F | Unisex- number of traditional pit latrines |  |
| Latrine Conditions | la_fl2 | la_fl2 | n | F | Unisex- number of KVIP (double) |  |
| Latrine Conditions | la_ul3 | la_ul3 | n | F | Unisex- number of VIP (single) |  |
| Latrine Conditions | la_ul4 | la_ul4 | n | F | Unisex- number of bucket/pan latrines |  |
| Latrine Conditions | la_ul5 | la_ul5 | n | F | Unisex- number of pour flush latrines |  |
| Latrine Conditions | la_ul6 | la_ul6 | n | F | Unisex- number of flush toilets |  |
| Latrine Conditions | la_us1 | la_us1 | n | F | Unisex- number with wood/natural slabs |  |
| Latrine Conditions | la_us2 | la_us2 | n | F | Unisex- number with cement slabs |  |
| Latrine Conditions | la_us3 | la_us3 | n | F | Unisex- number with dirt/unfinished slabs |  |
| Latrine Conditions | la_us4 | la_us4 | n | F | Unisex- number with porcelain/tile slabs |  |
| Latrine Conditions | la_uf1 | la_uf1 | n | F | Unisex- number with no feces present on slab or walls |  |
| Latrine Conditions | la_uf2 | la_uf2 | n | F | Unisex- number with some feces present on slab or walls |  |
| Latrine Conditions | la_uf3 | la_uf3 | n | F | Unisex- number with a lot of feces present on slab or walls |  |
| Latrine Conditions | la_ua1 | la_ua1 | n | F | Unisex- number with a secure stance |  |
| Latrine Conditions | la_ua2 | la_ua2 | n | F | Unisex- number with a slightly insecure stance |  |
| Latrine Conditions | la_ua3 | la_ua3 | n | F | Unisex- number with a stance so insecure there is a threat of falling through |  |
| Latrine Conditions | la_up1 | la_up1 | n | F | Unisex- number with a door that closes and have no holes |  |
| Latrine Conditions | la_up2 | la_up2 | n | F | Unisex- number with a door that doesn't close or have holes |  |
| Latrine Conditions | la_up3 | la_up3 | n | F | Unisex- number with no door |  |
| Latrine Conditions | la_ufly | la_ufly | n | F | Unisex- flies present | 1=None  2=Some  3=Swarms |
| Latrine Conditions | la_uod | la_uod | n | F | Unisex- odor present | 1=None  2=Some  3=Terrible |
| Latrine Conditions | la_af | la_af | n | F | Number of adult females |  |
| Latrine Conditions | la_afsh | la_afsh | n | F | Number of adult females wearing shoes |  |
| Latrine Conditions | la_afhw | la_afhw | n | F | Number of adult females who hw |  |
| Latrine Conditions | la_afso | la_afso | n | F | Number of adult females who hw with soap |  |
| Latrine Conditions | la_afcf | la_afcf | n | F | Number of adult females who bring child feces to dispose of |  |
| Latrine Conditions | la_am | la_am | n | F | Number of adult males |  |
| Latrine Conditions | la_amsh | la_amsh | n | F | Number of adult males wearing shoes |  |
| Latrine Conditions | la_amhw | la_amhw | n | F | Number of adult males who hw |  |
| Latrine Conditions | la_amso | la_amso | n | F | Number of adult males who hw with soap |  |
| Latrine Conditions | la_amcf | la_amcf | n | F | Number of adult males who bring child feces to dispose of |  |
| Latrine Conditions | la_yg | la_yg | n | F | Number of females 13-18 years |  |
| Latrine Conditions | la_ygsh | la_ygsh | n | F | Number of females 13-18 years wearing shoes |  |
| Latrine Conditions | la_yghw | la_yghw | n | F | Number of females 13-18 years who hw |  |
| Latrine Conditions | la_ygso | la_ygso | n | F | Number of females 13-18 years who hw with soap |  |
| Latrine Conditions | la_yb | la_yb | n | F | Number of males 13-18 years |  |
| Latrine Conditions | la_ybsh | la_ybsh | n | F | Number of males 13-18 years wearing shoes |  |
| Latrine Conditions | la_ybhw | la_ybhw | n | F | Number of males 13-18 years who hw |  |
| Latrine Conditions | la_ybso | la_ybso | n | F | Number of males 13-18 years who hw with soap |  |
| Latrine Conditions | la_cg | la_cg | n | F | Number of females 5-12 years |  |
| Latrine Conditions | la_cgsh | la_cgsh | n | F | Number of females 5-12 years wearing shoes |  |
| Latrine Conditions | la_cghw | la_cghw | n | F | Number of females 5-12 years who hw |  |
| Latrine Conditions | la_cgso | la_cgso | n | F | Number of females 5-12 years who hw with soap |  |
| Latrine Conditions | la_cb | la_cb | n | F | Number of males 5-12 years |  |
| Latrine Conditions | la_cbsh | la_cbsh | n | F | Number of males 5-12 years wearing shoes |  |
| Latrine Conditions | la_cbhw | la_cbhw | n | F | Number of males 5-12 years who hw |  |
| Latrine Conditions | la_cbso | la_cbso | n | F | Number of males 5-12 years who hw with soap |  |
| Latrine Exit | le_long | le_long | n | C | Longitude (DD) |  |
| Latrine Exit | le_lat | le_lat | n | C | Latitude (DD) |  |
| Latrine Exit | le_st | le_st | s | F | Start time |  |
| Latrine Exit | le_date | le_date | s | F | Date |  |
| Latrine Exit | le_notes | le_notes | n | F | Notes |  |
| Latrine Exit | le_a | le_a | n | F | Gender of respondent | 1=Male  2=Female |
| Latrine Exit | le_b | le_b | n | F | Age |  |
| Latrine Exit | le_c | le_c | n | F | Occupation | 1=Unemployed  2=Unskilled laborer  3=Professional worker  4=Skilled laborer  5=Student |
| Latrine Exit | le_co | le_co | s | F | Occupation other |  |
| Latrine Exit | le_d | le_d | n | F | The respondent is the head of the HH | 1=Yes  2=No  3=DK  4=No response |
| Latrine Exit | le_da | le_da | n | F | Occupation of the head of the HH |  |
| Latrine Exit | le_dao | le_dao | n | F | Other occupation for the head of the HH |  |
| Latrine Exit | le_e | le_e | n | F | The respondent's home is in this neighborhood | 1=Yes  2=No  3=DK  4=No response |
| Latrine Exit | le_f | le_f | n | F | Length of time living at current residence |  |
| Latrine Exit | le_fa | le_fa | n | F | Unit for length of time living at current residence |  |
| Latrine Exit | le_g | le_g | n | F | Tenancy status | 1=Rent  2=Own |
| Latrine Exit | le_h | le_h | n | F | Number of people in the HH |  |
| Latrine Exit | le_ha | le_ha | n | F | Number of adults (age 18 and older) in the HH |  |
| Latrine Exit | le_hb | le_hb | n | F | Number of school age children (5-17 years) in the HH |  |
| Latrine Exit | le_hc | le_hc | n | F | Number of children under 5 in the HH |  |
| Latrine Exit | le_i | le_i | n | F | Latrine in HH or compound | 1=Yes  2=No |
| Latrine Exit | le_ia | le_ia | n | F | Respondent is able to use the HH latrine | 1=Yes  2=No |
| Latrine Exit | le_ib | le_ib | n | F | Why the respondent chooses to use a public latrine |  |
| Latrine Exit | le_ic | le_ic | n | F | Why there is no latrine at the respondent's HH or compound | 1=Don't want one  2=Can't afford/cost  3=No space  4=Concerned about smell  5=No need/alternatives are available  6=Not yet constructed  7=Planning to move |
| Latrine Exit | le_ico | le_ico | n | F | Other reason for no latrine at the respondent's HH or compound |  |
| Latrine Exit | le_j | le_j | n | F | Location immediately before coming to latrine | 1=Place of residence  2=Place of work  3=Respondent’s school  4=Public transport |
| Latrine Exit | le_jo | le_jo | n | F | Other location immediately before coming to latrine |  |
| Latrine Exit | le_k | le_k | n | F | Length of time to reach latrine from this location |  |
| Latrine Exit | le_ka | le_ka | n | F | Units for length of time to reach latrine | 1=Minutes  2=Hours |
| Latrine Exit | le_l | le_l | n | F | Respondent stood in a queue before using the latrine | 1=Yes  2=No |
| Latrine Exit | le_la | le_la | n | F | Length of time in queue before using the latrine |  |
| Latrine Exit | le_laa | le_laa | n | F | Unit for length of time in queue before using latrine | 1=Minutes  2=Hours |
| Latrine Exit | le_m | le_m | n | F | Usual defecation place | 1=In bag/flying toilet  2=Latrine/toilet at home  3=Chamber pot at home  4=Open area  5=Public latrine  6=Latrine/toilet at work  7=Latrine/toilet at school |
| Latrine Exit | le_n | le_n | n | F | Number of visits to a public latrine last week |  |
| Latrine Exit | le_o | le_o | n | F | Respondent mostly used this latrine last week | 1=Yes  2=No |
| Latrine Exit | le_p | le_p | n | F | Time of day respondent visited latrine | 1=Morning  2=Noon  3=Afternoon  4=Evening  5=Midnight |
| Latrine Exit | le_q | le_q | n | F | What someone could do if they could not use a public latrine | 1=An open defecation site  2=A latrine/toilet in a public building  3=A latrine/toilet belonging to a friend  4=Use a bag/flying toilet  5=A latrine/toilet in my own home  6=DK |
| Latrine Exit | le_qa | le_qa | s | F | Other response if someone could not use a public latrine |  |
| Latrine Exit | le_r | le_r | n | F | Likelihood the respondent will have a latrine in their HH in one year | 1=Unlikely to be at this house in one year  2=Unlikely  3=Possible  4=Very likely |
| Non-Flood Prone | fn_date | fn_date | s | F | Date |  |
| Non-Flood Prone | fn_time | fn_time | s | F | Time |  |
| Non-Flood Prone | fn_lat | fn_lat | n | C | Latitude (DD) |  |
| Non-Flood Prone | fn_long | fn_long | n | C | Longitude (DD) |  |
| Non-Flood Prone | fn_notes | fn_notes | s | F | Notes |  |
| Norovirus 2 | n2_dateq | n2_dateq | s | F | Date QuantiFast was run |  |
| Norovirus 2 | n2_qfam | n2_qfam | n | F | QuantiFast FAM |  |
| Norovirus 2 | n2_qfamd | n2_qfamd | n | F | QuantiFast FAM duplicate run |  |
| Norovirus 2 | n2_qvic | n2_qvic | n | F | QuantiFast VIC |  |
| Norovirus 2 | n2_qvicd | n2_qvicd | n | F | QuantiFast VIC duplicate run |  |
| Norovirus 2 | n2_qintr | n2_qintr | n | C | Interpretation |  |
| Norovirus 2 | n2_dateo | n2_dateo | s | F | Date OneStep was run |  |
| Norovirus 2 | n2_ofam5 | n2_ofam3 | n | F | OneStep FAM(5uL) |  |
| Norovirus 2 | n2_ofam5d | n2_ofam4 | n | F | OneStep FAM(5uL) duplicate run |  |
| Norovirus 2 | n2_o5qty | n2_o5qty | n | F | OneStep FAM(5uL) quantity (GEC) |  |
| Norovirus 2 | n2_o5qtyd | n2_o5qt2 | n | F | OneStep FAM(5uL) quantity (GEC) duplicate run |  |
| Norovirus 2 | n2_o5log | n2_o5log | n | C | OneStep FAM(5uL) log quantity |  |
| Norovirus 2 | n2_o5logd | n2_o5lo2 | n | C | OneStep FAM(5uL) log quantity duplicate run |  |
| Norovirus 2 | n2_ofam1 | n2_ofam1 | n | F | OneStep FAM(1:5) |  |
| Norovirus 2 | n2_ofam1d | n2_ofam2 | n | F | OneStep FAM(1:5) duplicate run |  |
| Norovirus 2 | n2_o1qty | n2_o1qty | n | F | OneStep FAM(1:5) quantity (GEC) |  |
| Norovirus 2 | n2_o1qtyd | n2_o1qt2 | n | F | OneStep FAM(1:5) quantity (GEC) duplicate run |  |
| Norovirus 2 | n2_o1log | n2_o1log | n | C | OneStep FAM(1:5) log quantity |  |
| Norovirus 2 | n2_o1logd | n2_o1lo2 | n | C | OneStep FAM(1:5) log quantity duplicate run |  |
| Norovirus 2 | n2_fintr | n2_fintr | n | C | One Step interpretation |  |
| Norovirus 2 | n2_fintrd | n2_fint2 | n | C | One Step interpretation duplicate run |  |
| Norovirus 2 | n2_oqty | n2_oqty | n | C | One Step quantity |  |
| Norovirus 2 | n2_oqtyd | n2_oqtyd | n | C | One Step quantity duplicate run |  |
| Norovirus 2 | n2_ofam | n2_ofam | n | C | One Step FAM |  |
| Norovirus 2 | n2_ofamd | n2_ofamd | n | C | One Step FAM duplicate run |  |
| Norovirus 2 | n2_pos | n2_pos | n | C | Sample positive for GII | 1=Positive  2=Negative  3=Inhibition Positive  4=Inhibition Negative |
| Norovirus 2 | n2_denom | n2_denom | n | C | Denominator, equivalent volume |  |
| Norovirus 2 | n2_score | n2_score | s | C | reliability score (not ranked) | A1= Ct1>=41, Ct2=no signal  A2= Ct1=no signal, Ct2>=41  B= Ct1<41 and Ct2=no signal OR Ct1=no signal and Ct2<41  C= Ct difference<=4  D= Ct difference>4  E= Ct1=no signal, Ct2=no signal |
| Norovirus 2 | n2_conc | n2_conc | n | C | Concentration |  |
| Norovirus 2 | n2_logconc | n2_logco | n | C | Log 10 Concentration |  |
| Norovirus I | n1_dateq | n1_dateq | s | F | Date QuantiFast was run |  |
| Norovirus I | n1_qfam | n1_qfam | n | F | QuantiFast FAM |  |
| Norovirus I | n1_qfamd | n1_qfamd | n | F | QuantiFast FAM duplicate run |  |
| Norovirus I | n1_qvic | n1_qvic | n | F | QuantiFast VIC |  |
| Norovirus I | n1_qvicd | n1_qvicd | n | F | QuantiFast VIC duplicate run |  |
| Norovirus I | n1_qintr | n1_qintr | n | C | Interpretation |  |
| Norovirus I | n1_dateo | n1_dateo | s | F | Date OneStep was run |  |
| Norovirus I | n1_ofam5 | n1_ofam3 | n | F | OneStep FAM(5uL) |  |
| Norovirus I | n1_ofam5d | n1_ofam4 | n | F | OneStep FAM(5uL) duplicate run |  |
| Norovirus I | n1_o5qty | n1_o5qty | n | F | OneStep FAM(5uL) quantity (GEC) |  |
| Norovirus I | n1_o5qtyd | n1_o5qt2 | n | F | OneStep FAM(5uL) quantity (GEC) duplicate run |  |
| Norovirus I | n1_o5log | n1_o5log | n | C | OneStep FAM(5uL) log quantity |  |
| Norovirus I | n1_o5logd | n1_o5lo2 | n | C | OneStep FAM(5uL) log quantity duplicate run |  |
| Norovirus I | n1_ofam1 | n1_ofam1 | n | F | OneStep FAM(1:5) |  |
| Norovirus I | n1_ofam1d | n1_ofam2 | n | F | OneStep FAM(1:5) duplicate run |  |
| Norovirus I | n1_o1qty | n1_o1qty | n | F | OneStep FAM(1:5) quantity (GEC) |  |
| Norovirus I | n1_o1qtyd | n1_o1qt2 | n | F | OneStep FAM(1:5) quantity (GEC) duplicate run |  |
| Norovirus I | n1_o1log | n1_o1log | n | C | OneStep FAM(1:5) log quantity |  |
| Norovirus I | n1_o1logd | n1_o1lo2 | n | C | OneStep FAM(1:5) log quantity duplicate run |  |
| Norovirus I | n1_fintr | n1_fintr | n | C | One Step interpretation |  |
| Norovirus I | n1_fintrd | n1_fint2 | n | C | One Step interpretation duplicate run |  |
| Norovirus I | n1_oqty | n1_oqty | n | C | One Step quantity |  |
| Norovirus I | n1_oqtyd | n1_oqtyd | n | C | One Step quantity duplicate run |  |
| Norovirus I | n1_ofam | n1_ofam | n | C | One Step FAM |  |
| Norovirus I | n1_ofamd | n1_ofamd | n | C | One Step FAM duplicate run |  |
| Norovirus I | n1_pos | n1_pos | n | C | Sample positive for GI | 1=Positive  2=Negative  3=Inhibition Positive  4=Inhibition Negative |
| Norovirus I | n1_denom | n1_denom | n | C | Denominator, equivalent volume |  |
| Norovirus I | n1_score | n1_score | s | C | reliability score (not ranked) | A1= Ct1>=41, Ct2=no signal  A2= Ct1=no signal, Ct2>=41  B= Ct1<41 and Ct2=no signal OR Ct1=no signal and Ct2<41  C= Ct difference<=4  D= Ct difference>4  E= Ct1=no signal, Ct2=no signal |
| Norovirus I | n1_conc | n1_conc | n | C | Concentration |  |
| Norovirus I | n1_logconc | n1_logco | n | C | Log 10 Concentration |  |
| Nursery Conditions | ns_long | ns_long | n | C | Longitude (DD) |  |
| Nursery Conditions | ns_lat | ns_lat | n | C | Latitude (DD) |  |
| Nursery Conditions | ns_st | ns_st | s | F | Start time |  |
| Nursery Conditions | ne_et | ne_et | s | F | End time |  |
| Nursery Conditions | ns_date | ns_date | s | F | Date |  |
| Nursery Conditions | ns_notes | ns_notes | s | F | Notes |  |
| Nursery Conditions | ns_a | ns_a | n | F | Management type | 1=Public  2=Private |
| Nursery Conditions | ns_b | ns_b | n | F | Number of years in operation |  |
| Nursery Conditions | ns_c | ns_c | n | F | Cost of attendance |  |
| Nursery Conditions | ns_c1 | ns_c1 | n | F | Money units for cost of attendance | 1=Pesewas  2=Cedis |
| Nursery Conditions | ns_c2 | ns_c2 | n | F | Time units for cost of attendance | 1=Day  2=Term |
| Nursery Conditions | ns_dam | ns_dam | s | F | Opening time |  |
| Nursery Conditions | ns_dpm | ns_dpm | s | F | Closing time |  |
| Nursery Conditions | ns_d | ns_d | n | F | Total hours open |  |
| Nursery Conditions | ns_e | ns_e | n | F | Nursery has a latrine | 1=Yes  2=No |
| Nursery Conditions | ns_f | ns_f | n | F | Days the latrine is open | 1=Sunday  2=Monday  3=Tuesday  4=Wednesday  5=Thursday  6=Friday  7=Saturday  8=All days |
| Nursery Conditions | ns_f1 | ns_f1 | n | F | Total number of days the latrine is open |  |
| Nursery Conditions | ns_g1 | ns_g1 | n | F | Number of male children present today |  |
| Nursery Conditions | ns_g2 | ns_g2 | n | F | Number of female children present today |  |
| Nursery Conditions | ns_g3a | ns_g3a | n | F | Youngest child present today |  |
| Nursery Conditions | ns_g3a1 | ns_g3a1 | n | F | Unit for youngest child present today | 1=Months  2=Years |
| Nursery Conditions | ns_g3b | ns_g3b | n | F | Oldest child present today |  |
| Nursery Conditions | ns_g3b1 | ns_g3b1 | n | F | Unit for oldest child present today | 1=Months  2=Years |
| Nursery Conditions | ns_h1 | ns_h1 | n | F | Number of male children enrolled |  |
| Nursery Conditions | ns_h2 | ns_h2 | n | F | Number of female children enrolled |  |
| Nursery Conditions | ns_h3a | ns_h3a | n | F | Youngest child enrolled |  |
| Nursery Conditions | ns_h3a1 | ns_h3a1 | n | F | Unit for youngest child enrolled | 1=Months  2=Years |
| Nursery Conditions | ns_h3b | ns_h3b | n | F | Oldest child enrolled |  |
| Nursery Conditions | ns_h3b1 | ns_h3b1 | n | F | Unit for oldest child enrolled | 1=Months  2=Years |
| Nursery Conditions | ns_i1 | ns_i1 | n | F | Number of male teachers |  |
| Nursery Conditions | ns_i2 | ns_i2 | n | F | Number of female teachers |  |
| Nursery Conditions | ns_j | ns_j | s | F | Children's food sources | 1=Brought from home  2=Purchased from vendors  3=Provided by nursery |
| Nursery Conditions | ns_ja | ns_ja | n | C | Brought form home, from ns_j | 1=Yes  0=No |
| Nursery Conditions | ns_jb | ns_jb | n | C | Purchased from vendors, from ns_j | 1=Yes  0=No |
| Nursery Conditions | ns_jc | ns_jc | n | C | Provided by nursery, from ns_j | 1=Yes  0=No |
| Nursery Conditions | ns_k | ns_k | n | F | Children's primary drinking water source | 1=Sachets/water bottle  2=Tap from pipe network  3=Tap from polytank/bucket  4=Well  5=Water trucks  6=Rainwater  7=Other  8=DK  9=No response |
| Nursery Conditions | ns_l | ns_l | n | F | There is a storage container for water in the classroom | 1=Yes  2=No |
| Nursery Conditions | ns_l1 | ns_l1 | n | F | Type of water container | 1=Narrow mouth (≤6cm)  2=Wide mouth (>6cm)  3=Couldn't see |
| Nursery Conditions | ns_l2 | ns_l2 | n | F | Container is covered | 1=Covered  2=Uncovered |
| Nursery Conditions | ns_l3 | ns_l3 | n | F | How drinking water is taken from container | 1=Dip  2=Pour  3=Tap  4=Sachet  5=No water available |
| Nursery Conditions | ns_m | ns_m | n | F | Latrine type | 1=Traditional pit latrine  2=KVIP (double)  3=VIP (single)  4=Bucket/pan  5=Pour flush toilet  6=Flush toilet |
| Nursery Conditions | ns_n1 | ns_n1 | n | F | Size of room under observation (m2) |  |
| Nursery Conditions | ns_n2 | ns_n2 | n | F | Floor type | 1=Cement  2=Linoleum  3=Terrazo  4=Carpeted  5=Ceramic tiles  6=Dirt/unfinished |
| Nursery Conditions | ns_n3 | ns_n3 | n | F | Number of windows in room |  |
| Nursery Conditions | ns_o | ns_o | n | F | List of objects children can touch |  |
| Particulate | pa_lit | pa_lit | n | F | Sample was exposed to sunlight | 1=Yes  0=No |
| Particulate | pa_fec | pa_fec | n | F | Sample was taken within 3m of feces | 1=Yes  0=No |
| Particulate | pa_lat | pa_lat | n | F | Sample was taken within 30m of a latrine or defecation area | 1=Yes  0=No |
| Particulate | pa_type | pa_type | s | R | Type of particulate sample | 1=Sediment  2=Soil  3=Sand |
| Particulate | pa_where | pa_where | s | R | Location where sample was taken, if sand | 1=Near open water  2=Inland  3=Other |
| Particulate | pa_sot | pa_sot | s | F | Other location where sample was taken, if sand |  |
| Particulate | pa_areaent | pa_areae | n | F | Part of composite sample taken from area entrance | 1=Yes  0=No |
| Particulate | pa_strucent | pa_struc | n | F | Part of composite sample taken from structure entrance | 1=Yes  0=No |
| Particulate | pa_wc | pa_wc | n | F | Part of composite sample taken from WC/latrine | 1=Yes  0=No |
| Particulate | pa_cook | pa_cook | n | F | Part of composite sample taken from cooking area | 1=Yes  0=No |
| Particulate | pa_watera | pa_water | n | F | Part of composite sample taken from water area | 1=Yes  0=No |
| Particulate | pa_vend | pa_vend | n | F | Part of composite sample taken from vendor area | 1=Yes  0=No |
| Particulate | pa_cor1 | pa_cor1 | n | F | Part of composite sample taken from corner 1 | 1=Yes  0=No |
| Particulate | pa_cor2 | pa_cor2 | n | F | Part of composite sample taken from corner 2 | 1=Yes  0=No |
| Particulate | pa_cor3 | pa_cor3 | n | F | Part of composite sample taken from corner 3 | 1=Yes  0=No |
| Particulate | pa_cor4 | pa_cor4 | n | F | Part of composite sample taken from corner 4 | 1=Yes  0=No |
| Particulate | pa_cente | pa_cente | n | F | Part of composite sample taken from center | 1=Yes  0=No |
| Particulate | pa_play1 | pa_play1 | n | F | Part of composite sample taken from play area 1 | 1=Yes  0=No |
| Particulate | pa_play2 | pa_play2 | n | F | Part of composite sample taken from play area 2 | 1=Yes  0=No |
| Particulate | pa_play3 | pa_play3 | n | F | Part of composite sample taken from play area 3 | 1=Yes  0=No |
| Particulate | pa_wgt | pa_wgt | n | F | Sample weight (g) |  |
| School Conditions | sc_long | sc_long | n | C | Longitude (DD) |  |
| School Conditions | sc_lat | sc_lat | n | C | Latitude (DD) |  |
| School Conditions | sc_notes | sc_notes | s | F | Notes |  |
| School Conditions | sc_date | sc_date | s | F | Date |  |
| School Conditions | sc_101 | sc_101 | n | F | Respondent status | 1=Headmaster  2=Health coordinator  3=Teacher |
| School Conditions | sc_102 | sc_102 | n | F | School status | 1=Public  2=Private |
| School Conditions | sc_103 | sc_103 | s | F | Drinking water sources for students | 1=Sachets  2=Tap from pipe network  3=Tap from polytank  4=Well  5=Water trucks  6=Rainwater  7=DK  8=No response |
| School Conditions | sc_103 | sc_103 | n | C | Sachets, from sc_103 | 1=Yes  0=No |
| School Conditions | sc_103 | sc_103 | n | C | Tap from pipe network, from sc_103 | 1=Yes  0=No |
| School Conditions | sc_103 | sc_103 | n | C | Tap from polytank, from sc_103 | 1=Yes  0=No |
| School Conditions | sc_103 | sc_103 | n | C | Well, from sc_103 | 1=Yes  0=No |
| School Conditions | sc_103 | sc_103 | n | C | Water trucks, from sc_103 | 1=Yes  0=No |
| School Conditions | sc_103 | sc_103 | n | C | Rainwater, from sc_103 | 1=Yes  0=No |
| School Conditions | sc_103 | sc_103 | n | C | Don’t know, from sc_103 | 1=Yes  0=No |
| School Conditions | sc_103 | sc_103 | n | C | No response, from sc_103 | 1=Yes  0=No |
| School Conditions | sc_104 | sc_104 | n | F | Drinking water is provided for students | 1=Yes  2=No |
| School Conditions | sc_105 | sc_105 | n | F | Enumerator allowed to see how drinking water is stored | 1=Yes  2=No |
| School Conditions | sc_106 | sc_106 | n | F | Type of container | 1=Narrow mouth (≤6cm)  2=Wide mouth (>6cm)  3=Couldn't see |
| School Conditions | sc_107 | sc_107 | n | F | Container is covered | 1=Covered  2=Uncovered |
| School Conditions | sc_108 | sc_108 | n | F | Primary food source for students | 1=Brought from home  2=Purchased from vendors on school compound  3=Provided by school  4=Purchased from vendors away from school compound  5=DK  6=No response |
| School Conditions | sc_109 | sc_109 | n | F | Vendor food is sold at school | 1=Yes  2=No |
| School Conditions | sc_110 | sc_110 | n | F | Number of food vendors at school today |  |
| School Conditions | sc_111 | sc_111 | n | F | Percentage of students who buy food from these vendors |  |
| School Conditions | sc_111a | sc_111a | n | F | Don't know percentage of students who buy food from these vendors | 1=DK |
| School Conditions | sc_112 | sc_112 | n | F | School has a feeding program | 1=Yes  2=No |
| School Conditions | sc_113 | sc_113 | n | F | Percentage of students who get food from feeding program |  |
| School Conditions | sc_113a | sc_113a | n | F | Don't know percentage of students who get food from feeding program | 1=DK |
| School Conditions | sc_114 | sc_114 | n | F | Primary means of waste disposal for school | 1=Collection service  2=Disposal in compound  3=Disposal outside compound  4=Dump in a river  5=Burn  6=Other  7=DK  8=No response |
| School Conditions | sc_115 | sc_115 | n | F | There is a budget specifically for WASH at the school | 1=Yes  2=No  3=DK  4=No response |
| School Conditions | sc_116 | sc_116 | n | F | Number of latrines at school |  |
| School Conditions | sc_117 | sc_117 | n | F | Number of urinals at school |  |
| School Conditions | sc_118 | sc_118 | n | F | Someone is paid to clean latrines | 1=Yes  2=No  3=DK  4=No response |
| School Conditions | sc_119 | sc_119 | n | F | There is a HW station | 1=Yes  2=No |
| School Conditions | sc_120 | sc_120 | n | F | There is a budget for soap for HW | 1=Yes  2=No  3=DK  4=No response |
| School Conditions | sc_g1 | sc_g1 | n | F | Number of girls enrolled in class 1 | 999=Class does not exist at this school |
| School Conditions | sc_g2 | sc_g2 | n | F | Number of girls enrolled in class 2 | 999=Class does not exist at this school |
| School Conditions | sc_g3 | sc_g3 | n | F | Number of girls enrolled in class 3 | 999=Class does not exist at this school |
| School Conditions | sc_g4 | sc_g4 | n | F | Number of girls enrolled in class 4 | 999=Class does not exist at this school |
| School Conditions | sc_g5 | sc_g5 | n | F | Number of girls enrolled in class 5 | 999=Class does not exist at this school |
| School Conditions | sc_g6 | sc_g6 | n | F | Number of girls enrolled in class 6 | 999=Class does not exist at this school |
| School Conditions | sc_g7 | sc_g7 | n | F | Number of girls enrolled in class 7 | 999=Class does not exist at this school |
| School Conditions | sc_g8 | sc_g8 | n | F | Number of girls enrolled in class 8 | 999=Class does not exist at this school |
| School Conditions | sc_gt | sc_gt | n | F | Total number of girls enrolled | 999=Class does not exist at this school |
| School Conditions | sc_b1 | sc_b1 | n | F | Number of boys enrolled in class 1 | 999=Class does not exist at this school |
| School Conditions | sc_b2 | sc_b2 | n | F | Number of boys enrolled in class 2 | 999=Class does not exist at this school |
| School Conditions | sc_b3 | sc_b3 | n | F | Number of boys enrolled in class 3 | 999=Class does not exist at this school |
| School Conditions | sc_b4 | sc_b4 | n | F | Number of boys enrolled in class 4 | 999=Class does not exist at this school |
| School Conditions | sc_b5 | sc_b5 | n | F | Number of boys enrolled in class 5 | 999=Class does not exist at this school |
| School Conditions | sc_b6 | sc_b6 | n | F | Number of boys enrolled in class 6 | 999=Class does not exist at this school |
| School Conditions | sc_b7 | sc_b7 | n | F | Number of boys enrolled in class 7 | 999=Class does not exist at this school |
| School Conditions | sc_b8 | sc_b8 | n | F | Number of boys enrolled in class 8 | 999=Class does not exist at this school |
| School Conditions | sc_bt | sc_bt | n | F | Total number of boys enrolled | 999=Class does not exist at this school |
| Septage | sp_type | sp_type | n | R | Septage source | 1=Tanker truck  2=Sewage outfall  3=HH latrine  4=Public latrine  5=School/nursery latrine  6=Other |
| Septage | sp_ot | sp_ot | s | F | Other septage source |  |
| Septage | sp_wgt | sp_wgt | n | F | Sample weight (g) |  |
| Small Volume Drinking Water | sd_primw | sd_primw | n | R | Primary or secondary water source | 1=Primary water source  2=Secondary water source |
| Small Volume Drinking Water | sd_s | sd_s | n | F | Sachet water | 1=Yes  0=No |
| Small Volume Drinking Water | sd_sach | sd_sach | n | R | Sachet type | 1=Manufactured  2=Hand-tied |
| Small Volume Drinking Water | sd_smn | sd_smn | s | F | Sachet brand |  |
| Small Volume Drinking Water | sd_h | sd_h | n | F | Stored water other than sachet | 1=Yes  0=No |
| Small Volume Drinking Water | sd_source | sd_sourc | n | R | Stored water source | 1=Public tap  2=Compound/private tap  3=Hand-dug well  4=Tube well/borehole  5=Tanker truck  6=Other |
| Small Volume Drinking Water | sd_primo | sd_primo | s | F | Other store water source |  |
| Small Volume Drinking Water | sd_htx | sd_htx | n | F | Self-reported treated | 1=Yes  0=No |
| Small Volume Drinking Water | sd_htx1 | sd_htx1 | n | F | Self-reported treated with cloth | 1=Yes  0=No |
| Small Volume Drinking Water | sd_htx2 | sd_htx2 | n | F | Self-reported treated with a ceramic filter | 1=Yes  0=No |
| Small Volume Drinking Water | sd_htx3 | sd_htx3 | n | F | Self-reported treated by boiling | 1=Yes  0=No |
| Small Volume Drinking Water | sd_htx4 | sd_htx4 | n | F | Self-reported treated by solar | 1=Yes  0=No |
| Small Volume Drinking Water | sd_htx5 | sd_htx5 | n | F | Self-reported treated with chlorine | 1=Yes  0=No |
| Small Volume Drinking Water | sd_htx6 | sd_htx6 | n | F | Other self-reported treated | 1=Yes  0=No |
| Small Volume Drinking Water | sd_wtreat | sd_wtrea | n | R | When water was last treated | 1=Today  2=Yesterday  3=Two days ago  4=More than two days ago  5=DK  6=No response |
| Small Volume Drinking Water | sd_hpo | sd_hpo | s | F | Other type of treatment |  |
| Small Volume Drinking Water | sd_made | sd_made | n | R | Type of water storage container | 1=Earthen  2=Tile or concrete  3=Metal  4=Plastic  5=Other |
| Small Volume Drinking Water | sd_hcpp | sd_hcpp | n | F | Polytank if a plastic storage container | 1=Yes  0=No |
| Small Volume Drinking Water | sd_madeo | sd_madeo | s | F | Other type of water storage container |  |
| Small Volume Drinking Water | sd_hc | sd_hc | n | F | Container is covered | 1=Yes  0=No |
| Small Volume Drinking Water | sd_rem | sd_rem | n | R | How water is removed from container | 1=Cup used to remove water  2=Ladle used to remove water  3=Water is poured  4=Tap  5=Other |
| Small Volume Drinking Water | sd_ap | sd_ap | n | R | Container aperture | 1=Narrow mouth (≤6cm)  2=Wide mouth (>6cm) |
| Small Volume Drinking Water | sd_hp1 | sd_hp1 | n | F | Water used for drinking | 1=Yes  0=No |
| Small Volume Drinking Water | sd_hp2 | sd_hp2 | n | F | Water used for adult bathing | 1=Yes  0=No |
| Small Volume Drinking Water | sd_hp3 | sd_hp3 | n | F | Water used for laundry | 1=Yes  0=No |
| Small Volume Drinking Water | sd_hp4 | sd_hp4 | n | F | Water used for cooking | 1=Yes  0=No |
| Small Volume Drinking Water | sd_hp5 | sd_hp5 | n | F | Water used for child bathing | 1=Yes  0=No |
| Small Volume Drinking Water | sd_hp6 | sd_hp6 | n | F | Water used for HW | 1=Yes  0=No |
| Small Volume Drinking Water | sd_hp8 | sd_hp8 | n | F | Water used for rinsing food | 1=Yes  0=No |
| Small Volume Drinking Water | sd_hp9 | sd_hp9 | n | F | Water used for other | 1=Yes  0=No |
| Small Volume Drinking Water | sd_hpo | sd_hpo | s | F | Water used for other, specify | 1=Yes  0=No |
| Small Volume Drinking Water | sd_hp10 | sd_hp10 | n | F | Water used for irrigation | 1=Yes  0=No |
| Small Volume Drinking Water | sd_turb | sd_turb | n | F | Turbidity |  |
| Small Volume Drinking Water | sd_clf | sd_clf | n | F | Free chlorine residual |  |
| Small Volume Drinking Water | sd_clt | sd_clt | n | F | Total chlorine residual |  |
| Small Volume Drinking Water | sd_sal | sd_sal | n | F | Salinity |  |
| Small Volume Environmental Water | se_sr | se_sr | n | R | Water source | 1=Hand-washing buckets  2=Produce washing water  3=Other  4=Open drain |
| Small Volume Environmental Water | se_po | se_po | s | F | Other water source |  |
| Small Volume Environmental Water | se_sz | se_sz | n | R | Open drain size | 1=Small drain  2=Medium drain  3=Large drain |
| Small Volume Environmental Water | se_dhh | se_dhh | n | F | Drain is within 3m of HH | 1=Yes  0=No |
| Small Volume Environmental Water | se_lit | se_lit | n | F | Sample was exposed to sunlight | 1=Yes  0=No |
| Small Volume Environmental Water | se_f | se_f | n | F | Sample was taken near food | 1=Yes  0=No |
| Small Volume Environmental Water | se_fec | se_fec | n | F | Sample was taken within 3m of feces | 1=Yes  0=No |
| Small Volume Environmental Water | se_lat | se_lat | n | F | Sample was taken within 30m of a latrine or defecation area | 1=Yes  0=No |
| Small Volume Environmental Water | se_turb | se_turb | n | F | Turbidity |  |
| Small Volume Environmental Water | se_temp | se_temp | n | F | Temperature |  |
| Somatic Coliphage | cm_stim | cm_stim | n | R | Start time in incubator |  |
| Somatic Coliphage | cm_etim | cm_etim | n | R | End time in incubator |  |
| Somatic Coliphage | cm_stoim | cm_stoim | n | R | Start time for overnight incubator |  |
| Somatic Coliphage | cm_etoim | cm_etoim | n | R | End time for overnight incubator |  |
| Somatic Coliphage | cm_sdoid | cm_sdoid | n | R | Start date for overnight incubator |  |
| Somatic Coliphage | cm_edoid | cm_edoid | n | R | End data for overnight incubator |  |
| Somatic Coliphage | cm_dind | cm_dind | n | R | Date culture flask was inoculated |  |
| Somatic Coliphage | cm_dend | cm_dend | n | R | End date for e coli in incubator |  |
| Somatic Coliphage | cm_dil1 | cm_dil1 | n | F | Dilution 1 |  |
| Somatic Coliphage | dm_dil2 | dm_dil2 | n | F | Dilution 2 |  |
| Somatic Coliphage | cm_dil3 | cm_dil3 | n | F | Dilution 3 |  |
| Somatic Coliphage | cm_ccnt1 | cm_ccnt1 | n | F | Count for dilution 1 |  |
| Somatic Coliphage | cm_ccnt2 | cm_ccnt2 | n | F | Count for dilution 2 |  |
| Somatic Coliphage | cm_ccnt3 | cm_ccnt3 | n | F | Count for dilution 3 |  |
| Somatic Coliphage | cm_pdil1 | cm_pdil1 | n | F | Positive control, dilution 1 |  |
| Somatic Coliphage | cm_pdil2 | cm_pdil2 | n | F | Positive control, dilution 2 |  |
| Somatic Coliphage | cm_pdil3 | cm_pdil3 | n | F | Positive control, dilution 3 |  |
| Somatic Coliphage | cm_pcnt1 | cm_pcnt1 | n | F | Positive control count, dilution 1 |  |
| Somatic Coliphage | cm_pcnt2 | cm_pcnt2 | n | F | Positive control count, dilution 2 |  |
| Somatic Coliphage | cm_pcnt3 | cm_pcnt3 | n | F | Positive control count, dilution 3 |  |
| Somatic Coliphage | cm_cntb | cm_cntb | n | F | Count for blank |  |
| Somatic Coliphage | cm_denom | cm_denom | n | C | Denominator, equivalent volume |  |
| Somatic Coliphage | cm_score | cm_score | s | C | reliability score (not ranked) | B= All 3 plates with counts equal to 0  C= All 3 plates with counts over 200  D= Plate 1<20, Plate 2<20 or TNTC/TDTC, Plate 3<20 or TNTC/TDTC  A= all other combinations, where there was coliphage data |
| Somatic Coliphage | cm_select | cm_selec | n | C | determination of which plates to use for calculating concentration | 1= plate 1  2= plate 2  3= plate 3  12= plates 1 and 2  23= plates 2 and 3  123= plates 1, 2, and 3 |
| Somatic Coliphage | cm_conc | cm_conc | n | C | Concentration |  |
| Somatic Coliphage | cm_lnconc | cm_lncon | n | C | Log 10 Concentration |  |
| Student Tally | st_st | st_st | s | F | Start time at school |  |
| Student Tally | st_et | st_et | s | F | End time at school |  |
| Student Tally | st_date | st_date | s | F | Date |  |
| Student Tally | st_long | st_long | n | R | Longitude (DD) |  |
| Student Tally | st_lat | st_lat | n | R | Latitude (DD) |  |
| Student Tally | st_101 | st_101 | n | F | Class size |  |
| Student Tally | st_102a | st_102a | n | F | Youngest child in class |  |
| Student Tally | st_102b | st_102b | n | F | Oldest child in class |  |
| Student Tally | st_103 | st_103 | n | F | Start time |  |
| Student Tally | st_w1 | st_w1 | n | F | Number who like football |  |
| Student Tally | st_w2 | st_w2 | n | F | Number who are boys |  |
| Student Tally | st_201 | st_201 | n | F | Number who drank from a sachet today |  |
| Student Tally | st_202 | st_202 | n | F | Number who drank from a tippy tap today |  |
| Student Tally | st_203 | st_203 | n | F | Number who bought food from a vendor inside the school grounds today |  |
| Student Tally | st_204 | st_204 | n | F | Number who bought food from a vendor outside the school grounds today |  |
| Student Tally | st_205 | st_205 | n | F | Number who ate with their hands today |  |
| Student Tally | st_206 | st_206 | n | F | Number who ate kenke today |  |
| Student Tally | st_207 | st_207 | n | F | Number who ate ground nuts today |  |
| Student Tally | st_208 | st_208 | n | F | Number who ate an orange today |  |
| Student Tally | st_209 | st_209 | n | F | Number who ate waakye today |  |
| Student Tally | st_210 | st_210 | n | F | Number who ate with utensils today |  |
| Student Tally | st_211 | st_211 | n | F | Number who used the latrine today |  |
| Student Tally | st_212 | st_212 | n | F | Number who used the urinal today |  |
| Swab | sw_type | sw_type | n | R | Type of area swabbed | 1=Latrine wall  2=Other wall  3=Soap  4=Door handle  5=Children's toy  6=Food preparation surface  7=Anal cleansing container  8=Plate/cup/eating utensil  9=Other |
| Swab | sw_to | sw_to | s | F | Other type of area swabbed |  |
| Swab | sw_wet | sw_wet | n | R | Area is wet or dry | 1=Dry  2=Wet |
| Swab | sw_a1 | sw_a1 | n | F | Area height (cm) |  |
| Swab | sw_a2 | sw_a2 | n | F | Area width (cm) |  |
| Swab | sw_av | sw_av | n | F | Irregular area | 1=Yes  0=No |
| Swab | sw_child | sw_child | n | R | Child touched the area during observation period | 1=Yes  2=No  3=DK |
